# An investigation on 3-acetyl-7-methoxy-coumarin Schiff bases and their Ru(ii) metallates with potent antiproliferative activity and enhanced LDH and NO release†

**DOI:** 10.1039/c7ra12104k

**Published:** 2018-01-04

**Authors:** G. Kalaiarasi, S. Rex Jeya Rajkumar, S. Dharani, J. G. Małecki, R. Prabhakaran

**Affiliations:** Department of Chemistry, Bharathiar University Coimbatore 641 046 India rpnchemist@gmail.com +91-422-2422387 +91-422-2428319; Department of Biosciences and Technology, Karunya University Coimbatore 641 114 India; Department of Crystallography, Silsian University Szkolna 9 40-006 Katowice Poland

## Abstract

New cyclometallated ruthenium(ii) complexes of 3-acetyl-7-methoxycoumarin-4*N*-substituted thiosemicarbazones were synthesized and characterized by analytical and spectral techniques. The crystal structures of the ligands H_2_L^1–3^ and complexes (1, 2 and 4) were confirmed by X-ray crystallography. The analysis showed that the ligands have undergone C–H activation at the C(4) carbon of the pyrone ring and acted in a tridentate fashion by binding through C, N and S atoms. CT-DNA and protein (BSA/HSA) binding studies were carried out to analyze their interaction with biomolecules. Good binding affinity with DNA was observed with intercalative binding mode, which was further confirmed by EB displacement and viscosity measurement studies. The quenching mechanism with BSA/HSA was found to be static. Three dimensional (3D) fluorescence measurements were carried out to validate the micro environmental changes in the serum albumins. Their antioxidant propensity and antimicrobial study insisted that the compounds displayed good spectrum of activity. Evaluation of their anticancer potential against MCF-7 (human breast cancer) and A549 (human lung carcinoma) cell lines revealed that the complexes exhibited better activity than the ligands and cisplatin. Further, the results of LDH and NO release assays supported the cytotoxic nature of the compounds. The non-toxic nature of the compounds was established by testing against the non-cancerous cell line HaCaT (human normal keratinocyte).

## Abstract 2

New cyclometallated ruthenium(ii) complexes of 3-acetyl-7-methoxycoumarin-4*N*-substituted thiosemicarbazones were synthesized and characterized by analytical and spectral techniques.

## Graphical abstract

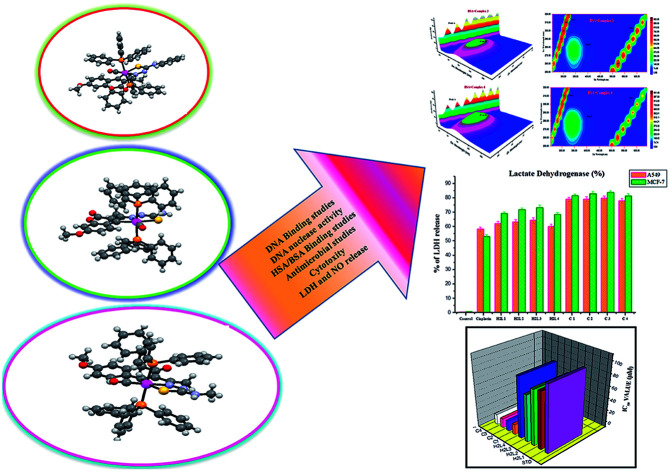

## Introduction

Thiosemicarbazides are a versatile class of compounds with indispensable properties such as antitumor, antifungal, antibacterial, antiviral, antiparasitic, *etc.*^1–6^ They are N, S donor ligands whose activity can be greatly enhanced by the presence of additional donor sites and a variety of coordination modes can be shown by these systems.^7–9^ Potential ligands are formed by attaching thiosemicarbazides with carbonyl compounds having a heterocyclic moiety. A good deal of research can be possible on such ligands and their metal complexes.^10^ It is already reported that the cytotoxicity of thiosemicarbazones is mostly related with their parent aldehyde or ketone, metal chelation efficacy and terminal amino substitution.^11,12^ Coumarin is one among the natural products found extensively in plants, which exhibits various pharmacological activities.^13,14^ Coumarin derived antibiotics such as novobiocin, clorobiocin and coumermycin A_1_ are commercialized.^15,16^ Their ability to inhibit human immunodeficiency virus integrase made them to be analysed in the treatment of HIV.^17^ Reports are available for testing coumarin derivatives against various tumor^18^ and neuronal cell lines.^19^ Generation of satisfactory clinical compounds is one of the promising approaches to attain effective and less toxic chemodrugs. The present scenario of inorganic research faces difficulties in raising highly active metal based drugs, the main criteria of which being less toxic and target specific. Among others, cancer stands as the most threatening disease and consistent attempts are made to develop appropriate chemotherapeutic drugs. Successful application of cisplatin as an anticancer agent provoked the chemists to search for other active metal complexes.^20,21^ Recently, ruthenium complexes have come into the lime light since two such complexes namely NAMI-A^22^ and KP1019 ^23^ entered into clinical trials, attesting the efficiency of ruthenium in the treatment of cancer. Moreover, exhibition of diverse coordination modes, stable oxidation states (from −2 to 8) and mimicking iron in binding biomolecules makes ruthenium a better alternative for platinum.^24^ Sufficient number of articles are available in the literature reporting the anticancer efficiency of ruthenium complexes including those by Garza-Ortiz *et al.*,^25^ and Juinn Chow *et al.*^26^

Considering the foregoing facts and our continuous investigation on thiosemicarbazone complexes of various transition metals, herein we report the synthesis, spectral characterization, X-ray diffraction analysis, DNA/protein binding, antioxidant, antimicrobial and anticancer studies of 3-acetyl-7-methoxycoumarin-4*N*-substituted thiosemicarbazones and their cyclometallated ruthenium(ii) complexes.

## Results and discussion

### Synthesis and characterization

**Scheme 1:**
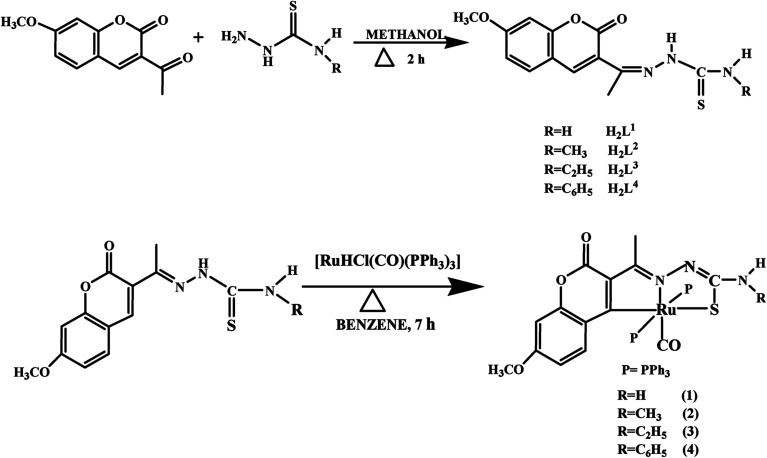
Synthesis of the ligands H_2_L^1–4^ and their new ruthenium(ii) complexes.

The ligands H_2_L^1–4^ were synthesized by the reaction of 3-acetyl-7-methoxy-2*H*-chromene-2-one with 4(*N*)-substituted thiosemicarbazides in methanol, which was precipitated as yellow solid from the reaction mixture. Reacting an equimolar mixture of these Schiff base ligands with [RuHClCO(PPh_3_)_3_] in benzene under reflux for 7 h afforded a reddish orange solution (Scheme 1). Characterization of the ligands and complexes were done by using elemental analyses, infrared spectroscopy, UV-Vis and NMR spectroscopy. The structures of the ligands H_2_L^1–3^ and complexes (1, 2 and 4) were confirmed by X-ray crystallographic study. X-ray crystal structural determination clearly showed that the ligands have undergone C–H activation at the *ortho* position of H_3_C–C=N and acted in a tridentate, bianionic manner binding through CNS atoms. Both the ligands and complexes are air stable and soluble in ethanol, chloroform, dichloromethane, toluene, methanol, DMSO and DMF. By using UV-visible spectroscopic techniques, the stability of the compounds in aqueous solutions was confirmed (Fig. S1 in the ESI†). The spectra recorded immediately and after 24 h did not show any appreciable change in the intensity and the position of the bands.

Assignments of selected characteristic IR band positions provided significant indication for the formation of 3-acetyl-7-methoxycoumarin-4(*N*)-substituted thiosemicarbazone ligands and their ruthenium complexes (Fig. S2–S10†). A band in the region 1600–1644 cm^−1^ is assigned to the *ν*(C=N),^27^ whereas the shifting of this band to 1606–1607 cm^−1^ revealed the coordination of azomethine nitrogen atom to the metal ion.^28,29^ A band appeared at 826–835 cm^−1^ in the ligands due to vibration of the C=S group, which disappeared in the spectra of the complexes and a new band corresponding to the C–S group appeared at 744–745 cm^−1^ indicating that the other coordination is through thiolate sulphur after enolization followed by deprotonation.^30^ In all the complexes, terminally coordinated carbonyl group appeared as a strong band in the region 1918–1925 cm^−1^.^29,30^ The stretching frequencies of triphenylphosphine group were observed around 1404–1434, 1087–1090, 694–696 cm^−1^.^30,31^

The electronic spectra of the ligands H_2_L^1–4^ and complexes 1–4 were recorded in DMSO. In the spectra of the free ligands, the higher energy absorption bands appeared around 275–282 nm have been assigned to (π → π*) transitions and the bands observed at 352–356 nm have been assigned to (n → π*) transitions.^32^ In the complexes 1–4, the high energy absorption band observed in the region 268–337 nm has been assigned to intra ligand transitions, the lowest energy band in the region 357–380 nm has been attributed to the ligand to metal charge transfer (LMCT) transitions.^7^

The ^1^H NMR spectra of 3-acetyl-7-methoxy-coumarin, 3-acetyl-7-methoxy-coumarin-4(*N*)-substituted thiosemicarbazone ligands H_2_L^1–4^ and their Ru(ii) complexes (1–4) showed the signals in the expected regions (Fig. S11–S19†). The singlets that appeared at *δ* 10.26–10.77 ppm were assigned to the hydrazinic proton of the free ligands, these signals were absent in the complexes (1–4), supporting the enolization and deprotonation of the NH–C=S group upon coordination of the thiolate sulfur to the Ru(ii) ion.^7,30^ The singlet due to the N=C–CH_3_ proton appeared at *δ* 2.24–2.33 ppm in the spectra of free ligand, which underwent an upfield shift in the spectra of the complexes to *δ* 1.88–2.09 ppm, suggesting that the coordination occurred *via* the nitrogen atom of N=C–CH_3_ group.^29,33,34^ The spectra of the ligands H_2_L^1–4^ displayed a singlet at *δ* 7.98–8.45 ppm, which is corresponding to the hydrogen atom at C(4) carbon, however, for complexes 1–4 no resonance could be attributed to C4(H), which indicated that the C(4) carbon atom of the pyrone ring is coordinated to the metal after deprotonation. In addition, the spectra of all the ligands and complexes exhibited a series of signals for aromatic protons at *δ* 6.29–7.74 ppm ^7,30^ and a singlet for –OCH_3_ protons around *δ* 3.74–3.93 ppm.^35^

### X-ray crystallography

#### Crystal structure of the ligands H_2_L^1–3^

**Fig. 1:**
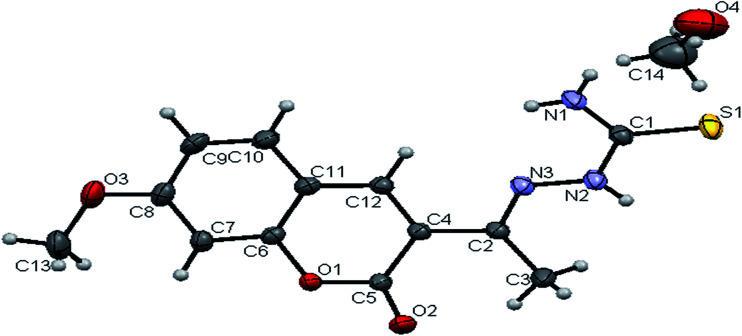
ORTEP diagram of [H_2_-7MAC-tsc] (H_2_L^1^).

**Fig. 2:**
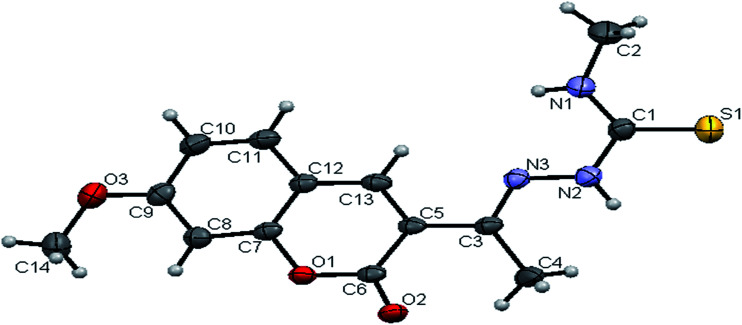
ORTEP diagram of [H_2_-7MAC-mtsc] (H_2_L^2^).

**Fig. 3:**
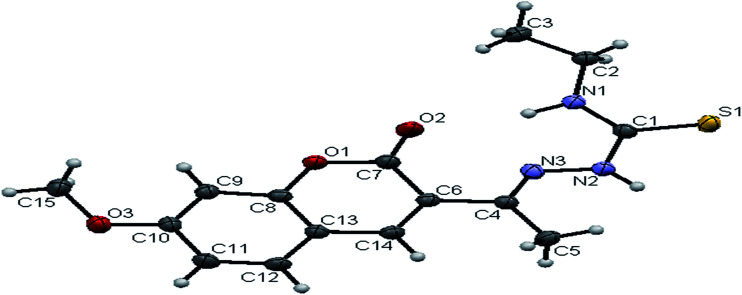
ORTEP diagram of [H_2_-7MAC-etsc] (H_2_L^3^).

The molecular structures of the ligands H_2_L^1–3^ have been determined by single-crystal X-ray diffraction studies. A summary of the structure refinement of the ligands is listed in Table 1 and selected inter atomic distances and bond angles are summarized in Table S1.† The structure of the ligand H_2_L^1^ together with the atom labelling scheme is shown in Fig. 1. The ligand H_2_L^1^ crystallized in the triclinic system with *P*1̄ space group. The monomeric units of the ligand H_2_L^1^ were arranged in a dimeric manner by the weaker N(1)–H(1)⋯O(1) hydrogen bonds (Fig. S20, Table S2†). The ligand H_2_L^2^ crystallized in the trigonal system with *P*3̄ space group. Six crystallographically independent molecules were present in the unit cell. In ligand H_2_L^2^, we found the donor–acceptor distance as 3.013 Å corresponding to the N(1)–O(1) and O(1)–N(1) bond between the two molecules (Fig. 2). This interaction gave a pseudo bimolecular appearance to the ligand H_2_L^2^ (Fig. S21†). The X-ray single crystal structure of the ligand H_2_L^3^ is shown in Fig. 3. From the symmetry of the reflections and solution of the structures, it is clear that the crystals belong to the triclinic *P*1̄ space group. H-Bonding in this structure is distinctly different from that observed in H_2_L^1^ and H_2_L^2^. The ligand H_2_L^3^ involved an intramolecular dipolar interaction between coumarin oxygen (O2) and azomethine nitrogen (N3) atoms as shown in Fig. S22 (Table S2†).

**Table 1:** Crystallographic data of ligands H_2_L^1^, H_2_L^2^ and H_2_L^3^

Identification code	[H_2_-7MAC-tsc]	[H_2_-7MAC-mtsc]	[H_2_-7MAC-etsc]
Empirical formula	C_13_H_13_N_3_O_3_S·CH_3_OH	C_14_H_15_N_3_O_3_S	C_15_H_17_N_3_O_3_S
Formula weight	323.36	305.35	319.38
Temperature	295(2) K	295(2) K	295(2) K
Wavelength	0.7107 Å	0.7107 Å	0.7107 Å
Crystal system	Triclinic	Trigonal	Triclinic
Space group	*P*1̄	*P*3̄	*P*1̄
**Unit cell dimensions**			
*a*	8.3702 (6) Å	17.5595 (9) Å	7.5260 (6) Å
*B*	8.8979(8) Å	17.5595 (9) Å	8.0281 (6) Å
*C*	12.4669 (9) Å	8.6657 (7) Å	13.5383 (10) Å
*α*	97.411 (7)°	90°	89.670 (6)°
*β*	106.008 (6)°	90°	87.437 (6)°
*γ*	113.670 (8)°	120°	67.782 (7)°
Volume	786.53 (12) Å^3^	2314.0 (3) Å^3^	756.43 (11) Å^3^
*Z*	2	6	2
Density	1.365 mg m^−3^	1.315 mg m^−3^	1.402 mg m^−3^
Absorption coefficient	0.227 mm^−1^	0.223 mm^−1^	0.230 mm^−1^
*F*(000)	340	960	336
Crystal size	0.02 × 0.08 × 0.34 mm	0.06 × 0.08 × 0.31 mm	0.13 × 0.14 × 0.38 mm
Crystal shape	Needle	Needle	Prism
*θ* range for data collection	4.223 to 27.424°	4.003 to 25.505°	4.100 to 28.715°
Limiting indices	−11 ≤ *h* ≤ 11, −12 ≤ *k* ≤ 12, −16 ≤ *l* ≤ 17	−22 ≤ *h* ≤ 24, −24 ≤ *k* ≤ 23, −7 ≤ *l* ≤ 11	−10 ≤ *h* ≤ 10, −11 ≤ *k* ≤ 10, −17 ≤ *l* ≤ 17
Reflections collected	2480	2007	2619
Independent reflections	3726 (*R*(int) = 0.0307)	3576 (*R*(int) = 0.0321)	3626 (*R*(int) = 0.0427)
Completeness to *θ*	26.32°	26.32°	26.32°
Absorption correction	Multi-scan	Multi-scan	Multi-scan
Refinement method	Full-matrix least-squares on *F*^2^	Full-matrix least-squares on *F*^2^	Full-matrix least-squares on *F*^2^
Data/restraints/parameters	3726/0/215	3576/0/201	3626/0/203
Goodness-of-fit on *F*^2^	1.042	1.007	1.055
Final *R* indices [*I* > 2*σ*(*I*)]	*R*_1_ = 0.0841, w*R*_2_ = 0.1760	*R*_1_ = 0.0553, w*R*_2_ = 0.1094	*R*_1_ = 0.0889, w*R*_2_ = 0.2249
*R* indices (all data)	*R*_1_ = 0.0984, w*R*_2_ = 0.2032	*R*_1_ = 0.1141, w*R*_2_ = 0.1298	*R*_1_ = 0.1151, w*R*_2_ = 0.2422

The existence of thione form in the ligands is confirmed by the C=S bond lengths, which are of 1.696 (3) Å, 1.677 (3) Å and 1.671 (4) Å for H_2_L^1^, H_2_L^2^ and H_2_L^3^ respectively and the bond length of C=N group (C(2)–N(3) = 1.289(3) Å for H_2_L^1^, C(3)–N(3) = 1.285(3) Å for H_2_L^2^, C(4)–N(3) = 1.273(5) Å for H_2_L^3^) is in agreement with a formal C=N bond length.^36^ In the ligands H_2_L^1–3^, the thione sulfur atom S(1) is *trans* to the N(3) nitrogen atom of C=N group about C(1)–N(2) bond, this structural arrangement corresponds to *E*-isomer, which is confirmed by a torsion angle of S(1)–C(1)–N(2)–N(3) bond, 179.6(2) for H_2_L^1^, 178.1(2) for H_2_L^2^ and 176.6(3) for H_2_L^3^. The bond distances in the 3-acetyl-7-methoxy-4(*N*)-thiosemicarbazones H_2_L^1–3^ agree well with the values observed for other thiosemicarbazones.^27,37,38^

#### Crystal structure description of new Ru(ii) complexes

**Fig. 4:**
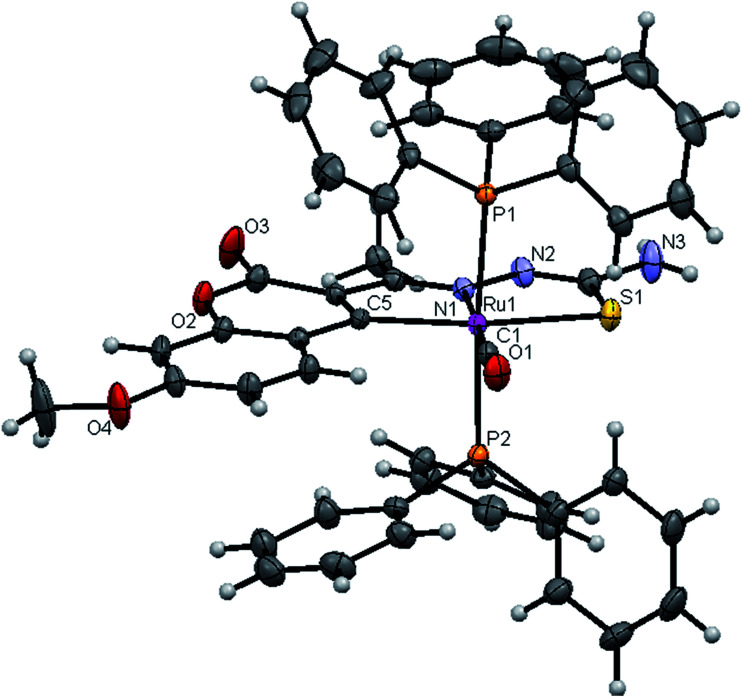
ORTEP diagram of [Ru(7MAC-tsc)CO(PPh_3_)_2_] (1).

**Fig. 5:**
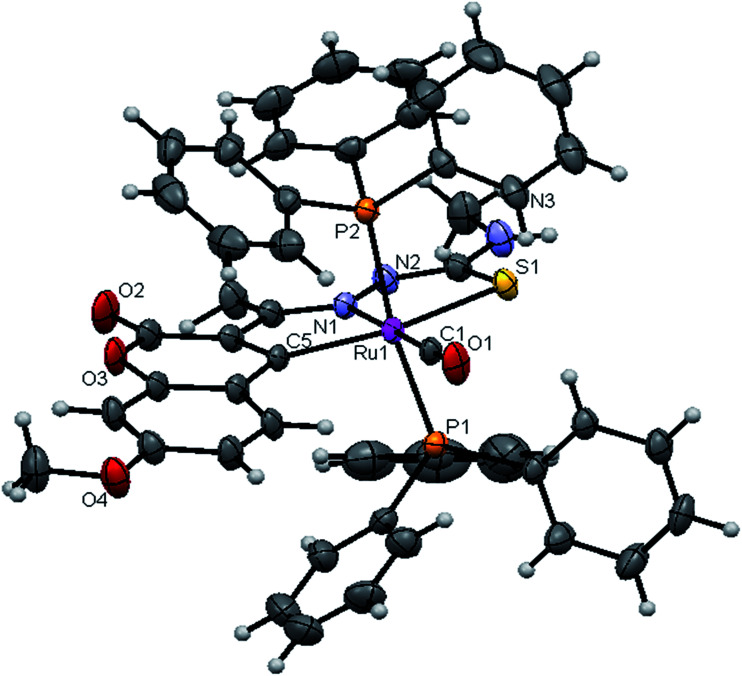
ORTEP diagram of [Ru(7MAC-mtsc)CO(PPh_3_)_2_] (2).

**Fig. 6:**
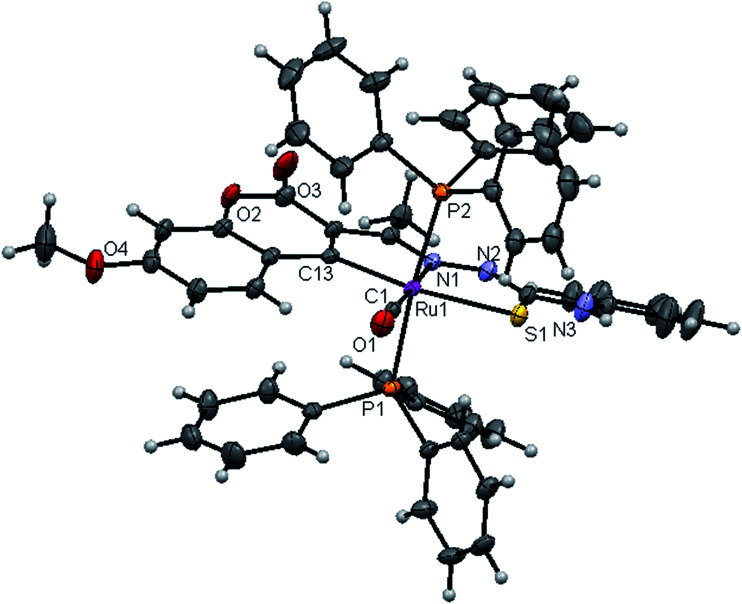
ORTEP diagram of [Ru(7MAC-ptsc)CO(PPh_3_)_2_] (4).

The molecular structures of the complexes (1, 2 and 4) have been determined by single crystal X-ray crystallographic studies to confirm the coordination modes of the 3-acetyl-7-methoxy-2*H*-chromene-2-one 4(*N*)-substituted thiosemicarbazones in the complexes. The summary of the data collection and the refinement parameters have been given in Table 2 whereas selected bond lengths and bond angles are given in Table S3.† The ORTEP view along with the atomic numbering scheme of complexes 1, 2 and 4 are given in Fig. 4–6. The X-ray crystal structures revealed that the complexes 1, 2 and 4 crystallized in the triclinic *P*1̄ space group. In the complexes, the ligands coordinated to the ruthenium ion through the N(1) nitrogen, pyrone carbon C(3) and thiolate sulphur atoms, forming two five member chelate rings with a bite angle N(1)–Ru(1)–S(1) of 78.39(6)° for complex 1, 78.90(1)° for complex 2, 79.38(6)° for complex 4, and a bite angle of C(5)–Ru(1)–N(1) of 78.58(8)° for complex 1, 78.10(2)° for complex 2, C(13)–Ru(1)–N(1) of 78.18(9)° for complex 4. The fourth site is occupied by the carbon atom of the carbonyl group to form a CNSC square-plane. The carbonyl group occupied the site *trans* to the N1 nitrogen, which is confirmed from the bond angle N(1)–Ru(1)–C(1) of 179.0(1)° for complex 1, 177.5(2)° for complex 2, and 178.10(2)° for complex 4 and bond length of Ru(1)–C(1) distances of 1.846(2) Å for complex 1, 1.853(4) Å for complex 2 and 1.848(2) Å for complex 4 found similar to the reported complexes.^29^ The remaining axial coordination sites are filled up by phosphorous atoms of two triphenylphosphine ligands, which are mutually *trans* to each other with Ru(1)–P(1) and Ru(1)–P(2) distances of 2.379(8) Å and 2.376(8) Å for complex 1, 2.365(1) Å and 2.383(1) Å for complex 2 and 2.376(8) Å and 2.376(8) Å for complex 4 and are slightly bent towards the carbonyl group due to the steric requirements of somewhat bulky chelating ligand, causing a slight deviation from a linear *trans* arrangement, which is evident from the bond angle of P(1)–Ru(1)–C(1) 89.41(8)° for complex 1, 88.3(1)° for complex 2, 90.17(8)° for complex 4, are smaller than bond angle of P(1)–Ru(1)–N(1) = 90.25(6)° for complex 1, P(1)–Ru(1)–N(1) = 93.3(1)° for complex 2, P(1)–Ru(1)–N(1) = 90.94(6)° for complex 4, and P(2)–Ru(1)–N(1) = 93.40(6)° for complex 1, P(2)–Ru(1)–N(1) = 89.2(1)° for complex 2, P(2)–Ru(1)–N(1) = 89.94(6)° for complex 4. The observed bond distances of Ru–P are comparable with those found in other reported ruthenium complexes containing triphenylphosphine.^7,29,30,39–41^ The bond distances of Ru(1)–P(1) and Ru(1)–P(2) are comparatively longer than those observed for basal planar bonds, such as Ru(1)–N(1) [2.077–2.097 Å], Ru(1)–C_pyrone_ [2.062–2.077 Å] and Ru(1)–C(1) [1.851–1.87 Å]. Due to the variation in the bond length and bond angles, ruthenium(ii) ion sitting in a CNOSP2 coordination environment and adopted a distorted octahedral geometry. The selected bond distances of complexes (1, 2 and 4) such as Ru–P, Ru–O, Ru–S, Ru–N and Ru–C and bond angles agree very well with the similar reported ruthenium(ii) complexes.^7,29–31,37,39–41^

**Table 2:** Crystallographic data of complexes 1, 2 and 4

Identification code	[Ru(7-MAC-tsc)(CO)(PPh_3_)_2_] (1)	[Ru(7-MAC-mtsc)(CO)(PPh_3_)_2_] (2)	[Ru(7-MAC-ptsc)(CO)(PPh_3_)_2_] (4)
Empirical formula	C_50_H_41_N_3_O_4_P_2_RuS·CH_3_OH	C_51_H_43_N_3_O_4_P_2_RuS	C_56_H_45_N_3_O_4_P_2_RuS
Formula weight	974.97	956.95	1019.02
Temperature	295(2) K	295(2) K	295(2) K
Wavelength	0.7107 Å	0.7107 Å	0.7107 Å
Crystal system	Triclinic	Triclinic	Triclinic
Space group	*P*1̄	*P*1̄	*P*1̄
**Unit cell dimensions**			
*a*	13.0569 (5) Å	12.2016 (7) Å	13.3038 (5) Å
*B*	13.7232 (5) Å	13.4519 (7) Å	13.4870 (5) Å
*C*	13.8479 (5) Å	15.1541 (9) Å	14.3074 (5) Å
*α*	89.094 (3)°	82.857 (4)°	85.881 (5)°
*β*	75.301 (3)°	79.550 (5)°	85.582 (5)°
*γ*	70.576 (3)°	65.934 (5)°	69.949 (3)°
Volume	2257.10 (15) Å^3^	2229.9 (2) Å^3^	2401.56 (16) Å^3^
*Z*	2	2	2
Density	1.435 mg m^−3^	1.425 mg m^−3^	1.409 mg m^−3^
Absorption coefficient	0.516 mm^−1^	0.520 mm^−1^	0.487 mm^−1^
*F*(000)	1004	984	1048
Crystal size	0.02 × 0.15 × 0.24 mm	0.08 × 0.22 × 0.26 mm	0.01 × 0.13 × 0.31 mm
Crystal shape	Plate	Plate	Plate
*θ* range for data collection	3.793 to 28.999°	3.605 to 28.757°	3.857 to 29.163°
Limiting indices	−15 ≤ *h* ≤ 17, −17 ≤ *k* ≤ 18, −14 ≤ *l* ≤ 18	−15 ≤ *h* ≤ 16, −17 ≤ *k* ≤ 18, −18 ≤ *l* ≤ 19	−17 ≤ *h* ≤ 17, −18 ≤ *k* ≤ 18, −19 ≤ *l* ≤ 15
Reflections collected	8354	7740	8210
Independent reflections	10 636 (*R*(int) = 0.0334)	10 577 (*R*(int) = 0.067)	11 129 (*R*(int) = 0.0376)
Completeness to *θ*	26.32°	26.32°	26.32°
Absorption correction	Multi-scan	Multi-scan	Multi-scan
Refinement method	Full-matrix least-squares on *F*^2^	Full-matrix least-squares on *F*^2^	Full-matrix least-squares on *F*^2^
Data/restraints/parameters	10 636/0/580	10 577/0/562	11 129/0/610
Goodness-of-fit on *F*^2^	1.034	1.092	1.016
Final *R* indices [*I* > 2*σ*(*I*)]	*R*_1_ = 0.0390, w*R*_2_ = 0.0833	*R*_1_ = 0.0839, w*R*_2_ = 0.2024	*R*_1_ = 0.0425, w*R*_2_ = 0.0824
*R* indices (all data)	*R*_1_ = 0.0582, w*R*_2_ = 0.0924	*R*_1_ = 0.1085, w*R*_2_ = 0.2330	*R*_1_ = 0.0689, w*R*_2_ = 0.0916

While dealing with the hydrogen-bonding interactions, in the complexes 2 and 4, we found the donor–acceptor distance (2.954 for (2), 3.054 Å for (4)) corresponding to the O(4)–O(4) bond between the carbonyl oxygen atom of the first molecule and with the second molecule. This interaction gave a pseudo binuclear structural appearance to the complex (2) and (4) (Fig. S23–S24; Table S2†).

### DNA binding studies

#### UV-Vis absorption spectral titrations

DNA-binding studies are important for the rational design and construction of new and more efficient drugs targeted to DNA.^42^ The binding affinity of the ligands and their organoruthenium(ii) complexes with CT-DNA can be measured by using UV-Vis spectroscopy. The UV-Vis absorption spectra of the free ligands and their complexes in the absence and presence of CT DNA are given in Fig. S25† and 7. In the presence of DNA, the absorption bands at about 345–349 nm for the ligands H_2_L^1–4^ exhibited hypochromism of about 41.46–48.41% accompanied by a small red shift. When the concentration of DNA is increased, all the new organoruthenium(ii) complexes (1–4) showed a decrease in absorbance in the charge transfer band at 330–348 nm to the extent of about 48.91–53.07% with a red-shift (bathochromic shift) of 2–8 nm. The decrease in absorbance with increase in concentration of CT-DNA may be due to the decrease in transition probabilities as a result of partial transfer of electrons from the π orbital of the DNA base pairs to the coupled π* orbital of the coordinated Schiff base to metal due to overlapping.^43^ The extent of hypochromism in the charge transfer band is an indication of the strength of intercalative interaction.^43^ The spectral characteristics obviously indicated that both the ligands and metal complexes interacted with DNA most likely through an intercalation mode involving a stacking interaction between the aromatic chromophore and base pairs of DNA.

**Fig. 7:**
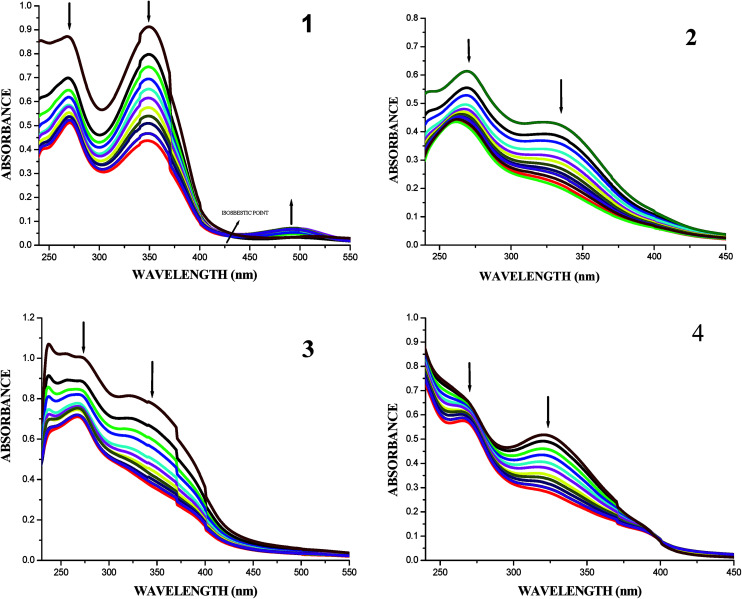
Absorption titration spectra of complexes (1–4) with increasing concentrations (2.5–25 μM) of CT-DNA (Tris–HCl buffer, pH 7.2).

**Fig. 8:**
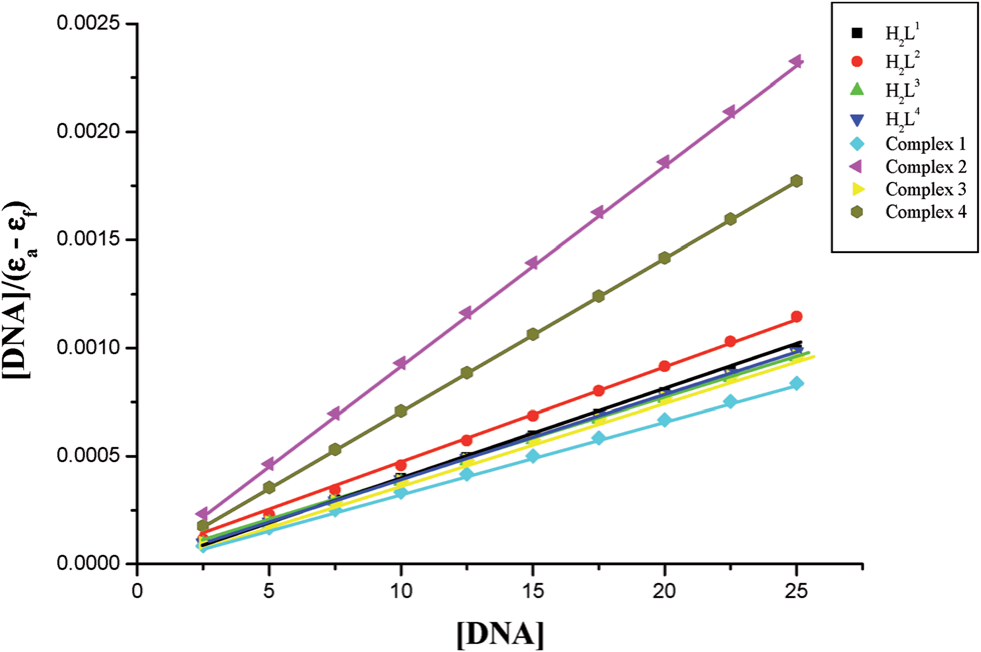
Binding isotherms of the ligands H_2_L^1–4^ and complexes 1–4 with CT-DNA.

In order to compare the DNA-binding affinities of the ligands and their metal complexes quantitatively, their intrinsic binding constants *K*_bin_ were obtained according to eqn (S1).† The binding constant value was calculated from the plot of [DNA]/(*ε*_a_ − *ε*_f_) *versus* [DNA] and the data were given in Table 3 (Fig. 8). From the results, the intrinsic DNA-binding constants *K*_bin_ were found to be in the order Complex 3 > Complex 2 > Complex 1 > Complex 4 > H_2_L^3^ > H_2_L^2^ > H_2_L^1^ > H_2_L^4^. This may be due to the presence of different substituents in the terminal nitrogen atom of the ligands. These results are comparable with earlier reports describing the intercalative mode of various ruthenium intercalators.^30,31,44^

**Table 3:** The binding constant (*K*_bin_) and quenching constant (*K*_SV_) values for the interaction of the ligands H_2_L^1–4^ and complexes (1–4) with CT-DNA

Compounds	Binding constant *K*_bin_ (M^−1^)	Quenching constant *K*_SV_ (M^−1^)
H_2_L^1^	2.1246 ± 0.308 × 10^5^	2.79 ± 0.005 × 10^3^
H_2_L^2^	3.4346 ± 0.306 × 10^5^	3.14 ± 0.002 × 10^3^
H_2_L^3^	7.1852 ± 0.304 × 10^5^	3.95 ± 0.009 × 10^3^
H_2_L^4^	2.1058 ± 0.288 × 10^5^	2.91 ± 0.005 × 10^3^
Complex 1	1.2544 ± 0.304 × 10^6^	4.16 ± 0.002 × 10^3^
Complex 2	1.3958 ± 0.320 × 10^6^	5.52 ± 0.009 × 10^3^
Complex 3	1.4428 ± 0.279 × 10^6^	5.62 ± 0.003 × 10^3^
Complex 4	1.0636 ± 0.334 × 10^6^	4.00 ± 0.002 × 10^3^

#### EB-DNA quenching studies

The competitive binding experiments were carried out on the EB–CT-DNA system by varying the concentrations of the ligands and complexes to get further information about the binding mode of compounds with DNA. In our studies, it was noted that upon concomitant addition of the compounds to the EB-DNA system, emission intensity decreased progressively, indicative of competition between EB and compounds towards CT-DNA in binding/chelation (Fig. S26†). The reduction of the fluorescence emission intensity gives criteria to investigate the DNA binding propensity of the compounds and stacking interaction (intercalation) between the adjacent DNA base pairs.^45^ As shown in Fig. S26,† the fluorescence intensity of EB-DNA gradually reduced with increasing concentrations of compounds indicating that the metal complexes bound to DNA by competing with EB. As the concentration of the compounds increased from 10–100 μM, the emission band of DNA-bound EB exhibited quenching upto 19.98, 24.07, 28.36, 23.38, 27.83, 36.67, 36.99 and 28.92% of the initial fluorescence intensity together with a red shift of 2–4 nm for H_2_L^1^, H_2_L^2^, H_2_L^3^, H_2_L^4^, complexes 1, 2, 3 and 4 respectively. This provides a direct evidence for the intercalative binding mode of the compounds with DNA.

**Fig. 9:**
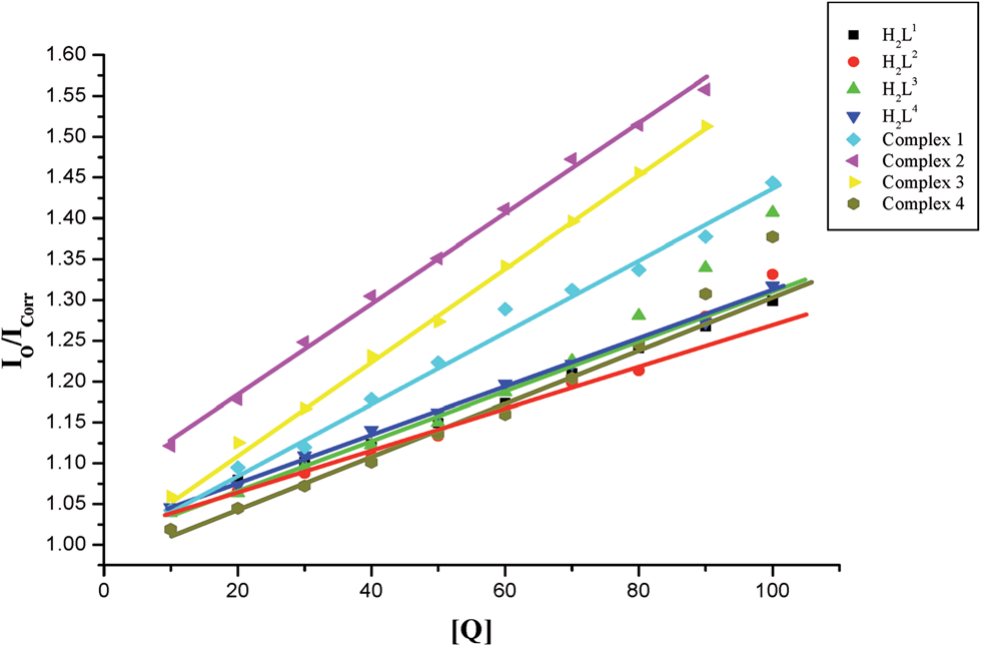
Stern–Volmer plot of the fluorescence titration of the ligands H_2_L^1–4^ and complexes (1–4) (10–100 μM) with DNA-EB (10 μM).

Further quantitative measurement of the magnitude of interaction was ascertained by the classical Stern–Volmer equation. Quenching constant *K*_SV_ is used to evaluate the quenching efficiency and is obtained from the slope of *I*_o_/*I versus* [Q] (Fig. 9) and given in Table 3. The experimental results showed that all the Ru(ii) complexes bind to DNA more strongly than their free ligands and the quenching constant value increased in the order 3 > 2 > 1 > 4 which also validated the electronic absorption spectral results. Further, the calculated *K*_SV_ values of the compounds are significant when compared to the reported values.^30,31^

#### Viscosity measurements

**Fig. 10:**
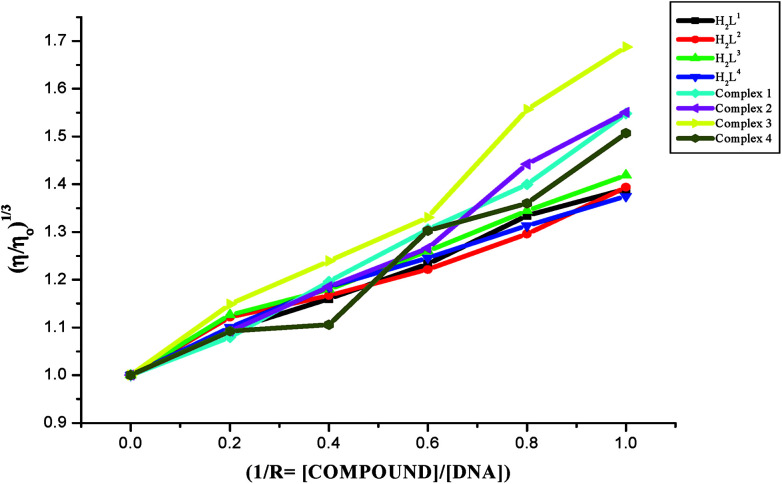
Effect of the ligands H_2_L^1–4^ and complexes (1–4) on the viscosity of CT-DNA.

Viscosity measurements are sensitive to changes of DNA length and are regarded as one of the most effective test for the binding mode of the compounds with DNA.^46,47^ In order to confirm the binding modes of the free ligands and their Ru(ii) complexes with CT-DNA, viscosity measurement study was carried out. The effects of the ligands and metal complexes on the relative viscosity of CT DNA are given in Fig. 10. From Fig. 10, it is obvious that the specific viscosity of the DNA sample increases with the addition of the compounds. Viscosity of DNA will increase while the complex intercalates between adjacent DNA base pairs, which leads to an increase in the separation of base pairs at the intercalation site, resulting an increase in the overall DNA length^48,49^ and the above results concluded that compounds interacted with CT-DNA through an intercalative mode. The results showed that the increasing rate of viscosity was different for ligands and complexes and the complexes exhibited a higher increasing rate due to the chelation of the ligands with Ru(ii) ion. The increased degree of viscosity may be depending upon the substitution on N-terminal nitrogen of the ligands and the increasing order of viscosity of CT-DNA by the compounds is complex 3 > complex 2 > complex 1 > complex 4 > H_2_L^3^ > H_2_L^2^ > H_2_L^1^ > H_2_L^4^, which is consistent with the above experimental results.

On the basis of above spectroscopic studies along with the viscosity measurements, it is revealed that the ligands and their organoruthenium(ii) complexes can bind to CT DNA *via* an intercalative mode and the new Ru(ii) complexes bind to CT DNA strongly than their free ligands alone.

#### DNA cleavage activity

**Fig. 11:**
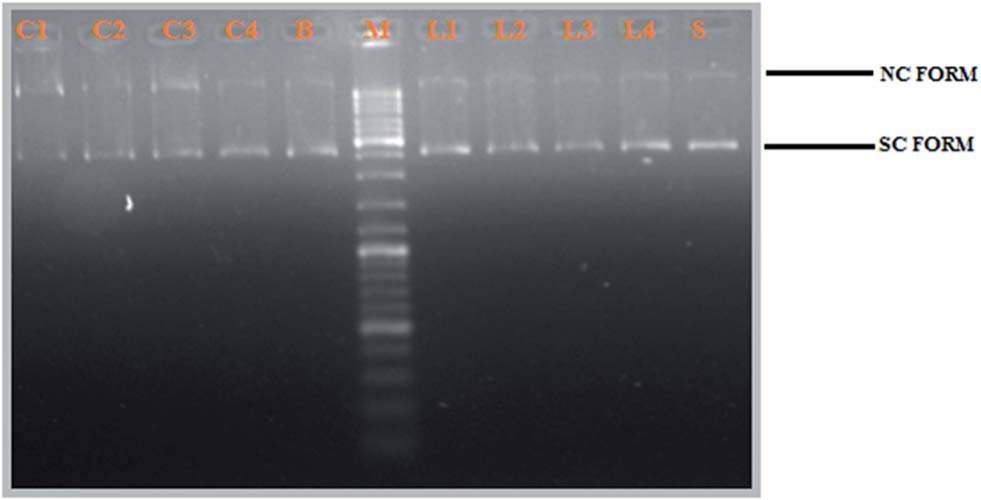
Gel electrophoresis diagram showing the cleavage of supercoiled pBR322 DNA by ligands H_2_L^1–4^ and complexes 1–4 in 5% DMSO and 95% 5 mM Tris–HCl/50 mM NaCl buffer at pH 7.2 and 37 °C with an incubation time of 2 h. Lanes M: marker; Lane L1: ligand 1 (50 μM). Lane L2: ligand 2 (50 μM); Lane L3: ligand 3 (50 μM); Lane L4: ligand 4 (50 μM); Lane 1: complex 1 (50 μM). Lane 2: complex 2 (50 μM); Lane 3: complex 3 (50 μM); Lane 4: complex 4 (50 μM); Lane S: metal precursor (50 μM); forms SC, NC, and LC are supercoiled, nicked circular, and linear circular DNA, respectively.

The newly synthesized Schiff base ligands H_2_L^1–4^ and their cyclometallated ruthenium(ii) complexes (1–4) were studied for their DNA cleavage activity by the method of agarose gel electrophoresis against supercoiled pBR322 DNA as the substrate, in the absence of external additives in a medium of 5 mM Tris–HCl/50 mM NaCl buffer (pH 7.2). The change in the DNA structure from supercoiled form to nicked or linear form produces change in the extent of migration in the gel. Moreover, one strand cleavage occurring in SC form will reduce to produce a nicked circular form (NC), which is a slower-moving form. If both strands are cleaved, linear circular (LC) form will be generated which migrates between SC and NC forms.^50^ For comparison purposes, plasmid DNA was incubated in presence of the representative ligands (H_2_L^1–4^) and their corresponding complexes (1−4) for 3 h at 37 °C. All the compounds efficiently cleaved the supercoiled pBR322 DNA to nicked form and linear circular form (Fig. 11). Obviously, the DNA cleaving efficacy of the ruthenium complexes are higher than that of the ligands, which correlates quite well with their DNA binding affinity. From Fig. 11, we knew that the complexes showed potent nuclease activity without any external reagent and complex 3 with more electron donating ethyl substitution on terminal nitrogen atom causes stronger distortion on DNA strand leading to more efficient DNA cleavage followed by complex 2, complex 1 and complex 4. This resulted pattern is consistent with the DNA binding studies results.

#### Protein binding studies

**Fig. 12:**
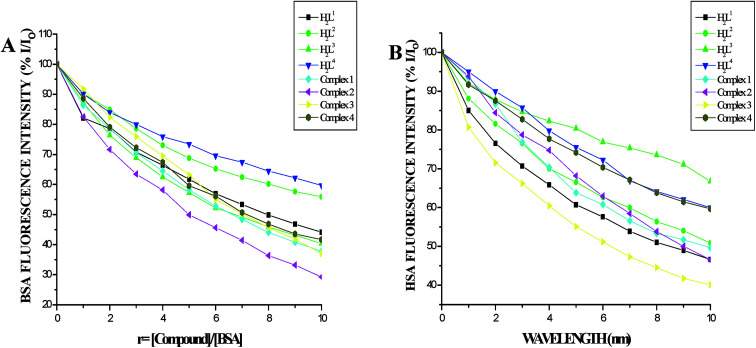
(A) Plot of % relative fluorescence intensity (% *I*/*I*_o_) *vs. r* (*r* = [compound]/[BSA]) (B) plot of % relative fluorescence intensity (% *I*/*I*_o_) *vs. r* (*r* = [compound]/[HSA]).

**Fig. 13:**
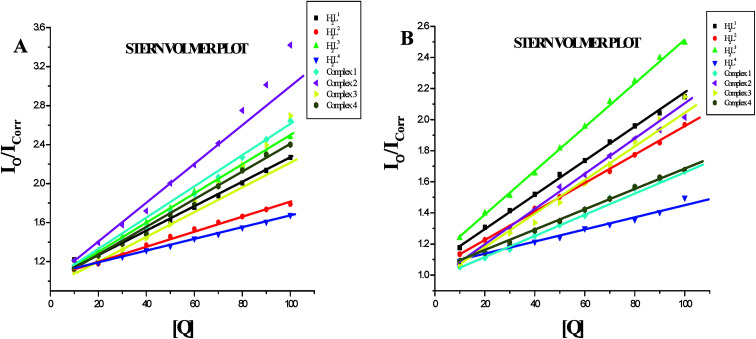
(A) Stern–Volmer plot of the fluorescence titration of the ligands H_2_L^1–4^ and complexes (1–4) (10–100 μM) with BSA (10 μM). (B) Stern–Volmer plot of the fluorescence titration of the ligands H_2_L^1–4^ and complexes (1–4) (10–100 μM) with HSA (10 μM).

In order to investigate the binding of BSA and its homologue HSA with the ligands and new Ru(ii) complexes, the quenching of its fluorescence emission spectra upon addition of compounds has been studied (Fig. S27 and S28†), since the albumin solution exhibits an intense emission band (*λ*_ex_ = 290 nm) at *λ*_em,max_ = 345 nm (for HSA) and 346 nm (for BSA) which is assigned to the existence of tryptophans.^51,52^ Addition of the compounds to BSA resulted in the quenching of its fluorescence intensity at 346 nm upto 55.22%, 43.73%, 43.92%, 39.87%, 62.61%, 71.15%, 63.19% and 58.68% for H_2_L^1^, H_2_L^2^, H_2_L^3^, H_2_L^4^, complex 1, complex 2, complex 3 and complex 4 respectively with a 2–4 nm of hypsochromic shift (Fig. 12A). The fluorescence intensities of HSA decreased upto 53.86%, 46.41%, 51.00%, 28.61%, 41.01%, 50.56%, 53.45% and 39.80% for H_2_L^1^, H_2_L^2^, H_2_L^3^, H_2_L^4^, complex 1, complex 2, complex 3 and complex 4 respectively with an increase in the concentration of the compounds, accompanied by a blue shift of 2–5 nm (Fig. 12B). The obtained results confirmed the interaction of the ligands and complexes with serum albumins. The absorption spectra of the serum albumins in the absence and presence of ligands H_2_L^1–4^ and the complexes 1–4 are given in Fig. S29 in the ESI.† On adding ligands and complexes 1–4 to albumins, the absorbance intensity of serum albumins was decreased with a red shift of 2 nm. The observed changes indicated a static quenching mechanism of serum albumins by the ligands and complexes (1–4). To obtain a quantitative insight into the quenching progression, the Stern–Volmer quenching constant (*K*_SV_) and the quenching constant (*K*_q_) were calculated from the Stern–Volmer equation using the *I*_o_/*I*_Corr_*versus* [Q] plot (Table 4).^53^ The observed linearity in the plots (Fig. 13) indicated the ability of the compounds to quench the emission intensity of serum albumins and the order of quenching constant of the compounds is complex 3 > complex 2 > complex 1 > complex 4 > H_2_L^3^ > H_2_L^2^ > H_2_L^1^ > H_2_L^4^. The observed *K*_SV_ values are comparable to those reported for other ruthenium complexes and this result is consistent with the pattern in DNA binding studies.^30,44^ The quenching constant values for the quenching of serum albumins by the compounds (*k*_q_ ≈ 10^12^ M^−1^ s^−1^) suggested a good binding affinity through static quenching mechanism.^53^

**Table 4:** Stern–Volmer quenching constant (*K*_SV_), quenching constant (*k*_q_), binding constant (*K*_bin_) and number of binding sites (*n*) for the interactions of ligands and complexes (1–4) with BSA/HSA

Compounds	Stern–Volmer *K*_SV_/M^−1^	Quenching constant *k*_q_/M^−1^ s^−1^	Binding constant *K*_bin_/M^−1^	*n*
BSA				
(H_2_L^1^)	1.193 ± 0.024 × 10^4^	1.193 ± 0.024 × 10^12^	1.971 ± 0.042 × 10^3^	0.8075 ± 0.029
(H_2_L^2^)	7.730 ± 0.009 × 10^3^	0.773 ± 0.009 × 10^12^	3.003 ± 0.010 × 10^3^	0.8915 ± 0.011
(H_2_L^3^)	1.464 ± 0.018 × 10^4^	1.464 ± 0.018 × 10^12^	14.11 ± 0.030 × 10^3^	0.9947 ± 0.010
(H_2_L^4^)	6.660 ± 0.012 × 10^3^	0.666 ± 0.012 × 10^12^	1.111 ± 0.018 × 10^3^	0.8039 ± 0.021
Complex 1	1.674 ± 0.040 × 10^4^	1.674 ± 0.040 × 10^12^	2.251 ± 0.029 × 10^4^	1.0408 ± 0.030
Complex 2	2.382 ± 0.011 × 10^4^	2.382 ± 0.011 × 10^12^	3.323 ± 0.027 × 10^4^	1.0478 ± 0.031
Complex 3	1.756 ± 0.080 × 10^4^	1.756 ± 0.080 × 10^12^	17.49 ± 0.030 × 10^4^	1.266 ± 0.0290
Complex 4	1.454 ± 0.030 × 10^4^	1.454 ± 0.030 × 10^12^	2.178 ± 0.019 × 10^4^	1.0486 ± 0.019

HSA				
(H_2_L^1^)	1.073 ± 0.014 × 10^4^	1.073 ± 0.014 × 10^12^	2.095 ± 0.036 × 10^3^	0.8166 ± 0.010
(H_2_L^2^)	0.909 ± 0.010 × 10^4^	0.909 ± 0.010 × 10^12^	2.549 ± 0.016 × 10^3^	0.8599 ± 0.017
(H_2_L^3^)	1.424 ± 0.016 × 10^4^	1.424 ± 0.016 × 10^12^	2.615 ± 0.019 × 10^3^	0.8130 ± 0.021
(H_2_L^4^)	0.377 ± 0.010 × 10^4^	0.377 ± 0.010 × 10^12^	1.165 ± 0.018 × 10^3^	0.8702 ± 0.018
Complex 1	0.741 ± 0.010 × 10^4^	0.741 ± 0.010 × 10^12^	2.273 ± 0.017 × 10^4^	1.1329 ± 0.018
Complex 2	1.088 ± 0.030 × 10^4^	1.088 ± 0.030 × 10^12^	6.341 ± 0.039 × 10^4^	1.1859 ± 0.004
Complex 3	1.189 ± 0.030 × 10^4^	1.189 ± 0.030 × 10^12^	7.194 ± 0.041 × 10^4^	1.2027 ± 0.030
Complex 4	0.675 ± 0.008 × 10^4^	0.675 ± 0.008 × 10^12^	0.297 ± 0.024 × 10^4^	0.9118 ± 0.025

**Fig. 14:**
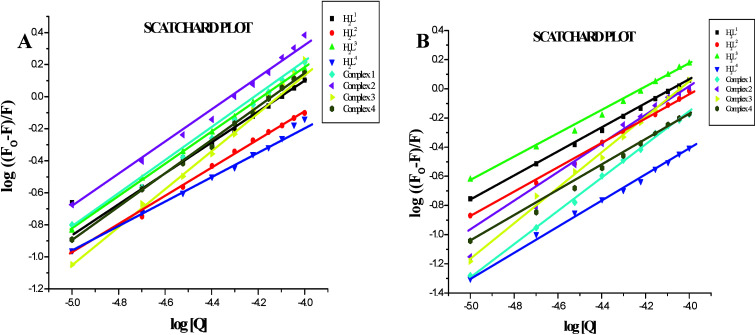
(A) Scatchard plot of the fluorescence titration of the ligands H_2_L^1–4^ and complexes (1–4) (10–100 μM) with BSA (10 μM). (B) Scatchard plot of the fluorescence titration of the ligands H_2_L^1–4^ and complexes (1–4) (10–100 μM) with HSA (10 μM).

Furthermore, the equilibrium binding constant and number of binding sites were evaluated by using the Scatchard equation. The *K*_bin_ values were derived from the graph between log[(*F*_o_ − *F*)/*F*] and log[Q] (Fig. 14) and are given in Table 4. From the results, we confirmed that the Ru(ii) complexes having a large hydrophobic area can interact more efficiently than the ligands with serum albumins *via* a static pathway. The higher binding affinity of the complexes over the ligands may be due to the efficient binding of protein moiety with the complexed metal ions. Here, the binding affinities of the complexes with serum albumins followed the same order as those with DNA binding studies, complex 3 > complex 2 > complex 1 > complex 4 > H_2_L^3^ > H_2_L^2^ > H_2_L^1^ > H_2_L^4^. Variation in the binding affinity of the compounds with serum albumins depends on the electron-donating ability of the ligand, *i.e.* the substitution on the N-terminal nitrogen atom. The binding capability of the compounds to serum albumins increased with the increase in electron-donating ability of the substituent on the terminal nitrogen of the coordinated thiosemicarbazone ligand. The obtained quenching constant and binding constant values of these new cyclometallated ruthenium(ii) complexes agree well with those reported for other ruthenium(ii) complexes.^31,44,54–56^

#### Conformational investigation

The conformational changes of the protein molecular environment in the vicinity of the fluorophore functional groups have been investigated by synchronous fluorescence spectroscopy. Synchronous fluorescence spectra show Trp residues of serum albumins only at the wavelength interval (Δ*λ*) of 60 nm and Tyr residues only at Δ*λ* of 15 nm. For both Δ*λ* = 15 and 60 nm, fluorescence intensities have been decreased with an increasing amount of compounds (Fig. S30–S33†). However, the magnitude of quenching and shift of wavelength are greater at Δ*λ* = 60 nm. This result showed that compounds interacted with both Trp and Tyr residues but in greater magnitude with Trp residues.

#### Three-dimensional fluorescence spectra analysis

**Fig. 15:**
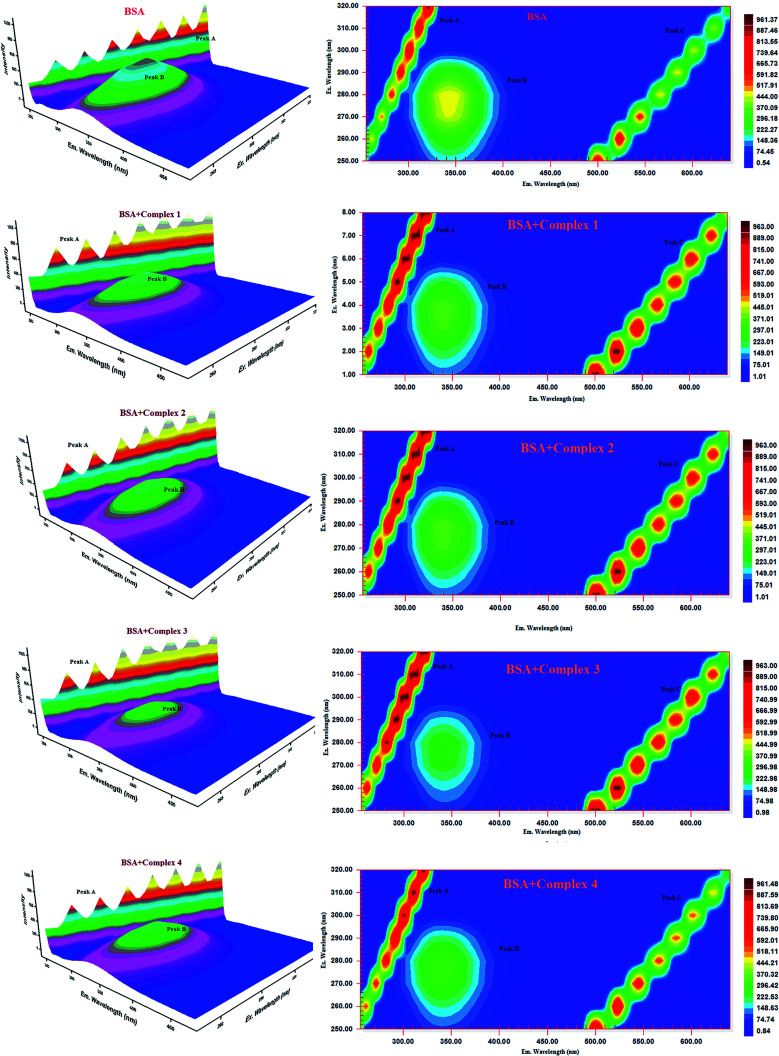
Three-dimensional fluorescence spectra of BSA in the absence and presence of ruthenium(ii) complexes 1–4 (pH 7.4, 298 K, [BSA] = 10 μM, [complex] = 10 μM).

**Fig. 16:**
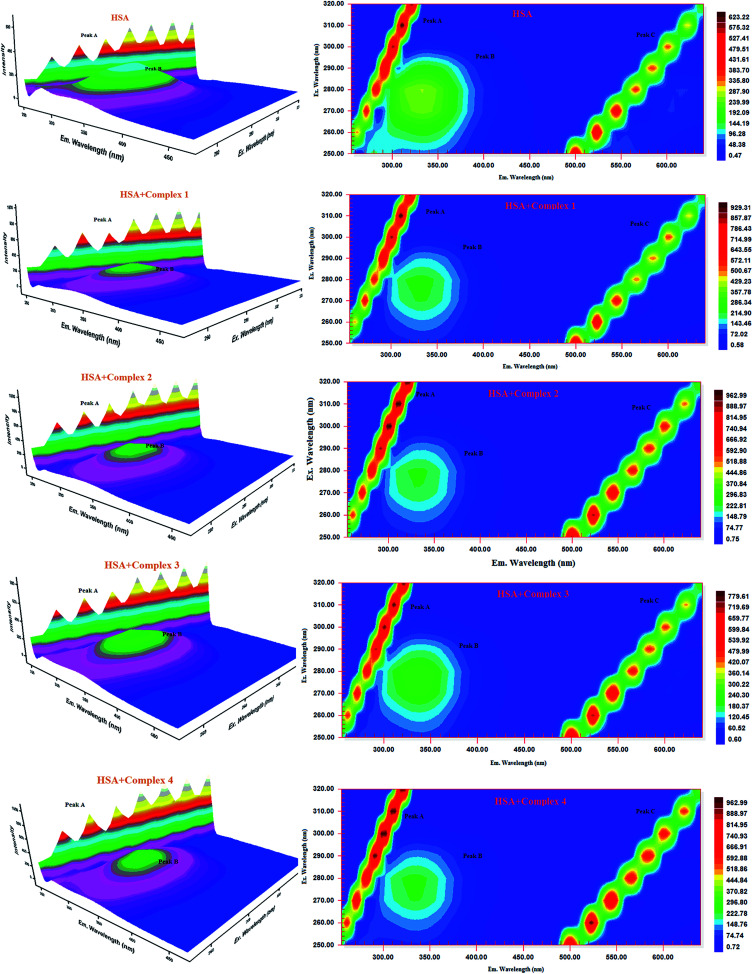
Three-dimensional fluorescence spectra of HSA in the absence and presence of ruthenium(ii) complexes 1–4 (pH 7.4, 298 K, [HSA] = 10 μM, [complex] = 10 μM).

To investigate the micro environmental changes in BSA/HSA during interaction with the compounds, three dimensional fluorescence spectroscopic studies have been performed. The changes observed in 3D emission spectra and contour lines of serum albumins in the absence and presence of ligands and complexes are given in Fig. 15, 16, S34 and S35 in the ESI† and their corresponding characteristic parameters are provided in Table 5. The emission spectra of serum albumins such as BSA and HSA have shown three characteristic peaks – peaks A and C corresponds to first and second order Rayleigh scattering and peak B corresponds to the spectral characteristics of Trp and Tyr residues of proteins.^57^ The emission intensity of Rayleigh first order scattering peak increased upon adding the compounds to serum albumins. This is due to the fluorophore-quencher complex formation of serum albumins with our ligands/ruthenium(ii) complexes leading to an increase in the diameter of the macromolecule which in turn resulted in the enhancement of scattering effect.^58^ The fluorescence intensity of peak ‘B’ corresponding to the tryptophan and tyrosine residues decreased with slight blue shift. From the results, it is inferred that the molecular microenvironment and conformational changes of protein occurred after interaction with the complexes and ligands.

**Table 5:** Three-dimensional fluorescence spectral characteristics of BSA/HSA and BSA/HSA-complexes systems

Compounds	Rayleigh scattering peaks			Fluorescence peaks		
	Peak position *λ*_ex_/*λ*_em_ (nm nm^−1^)	Stokes Δ*λ* (nm)	Intensity (*F*)	Peak position *λ*_ex_/*λ*_em_ (nm nm^−1^)	Stokes Δ*λ* (nm)	Intensity (*F*)
BSA						
BSA	280/280	0	637.73	280/343	63	514.37
BSA + H_2_L^1^	280/280	0	681.98	280/340	60	436.40
BSA + H_2_L^2^	280/280	0	704.90	280/342	62	437.11
BSA + H_2_L^3^	280/280	0	764.34	280/342	62	415.28
BSA + H_2_L^4^	280/280	0	751.37	280/342	62	486.41
BSA + complex 1	280/280	0	867.32	280/338	58	395.56
BSA + complex 2	280/280	0	837.83	280/339	59	372.76
BSA + complex 3	280/280	0	958.23	280/339	59	304.43
BSA + complex 4	280/280	0	741.59	280/340	60	368.76

HSA						
HSA	280/280	0	480.02	280/333	53	289.67
HSA + H_2_L^1^	280/280	0	563.09	280/335	55	281.73
HSA + H_2_L^2^	280/280	0	647.85	280/332	52	278.16
HSA + H_2_L^3^	280/280	0	582.94	280/336	56	275.92
HSA + H_2_L^4^	280/280	0	659.33	280/334	54	281.01
HSA + complex 1	280/280	0	652.04	280/335	55	268.14
HSA + complex 2	280/280	0	790.59	280/331	51	269.53
HSA + complex 3	280/280	0	612.58	280/337	57	266.21
HSA + complex 4	280/280	0	830.46	280/332	52	276.12

#### Antioxidant studies

**Fig. 17:**
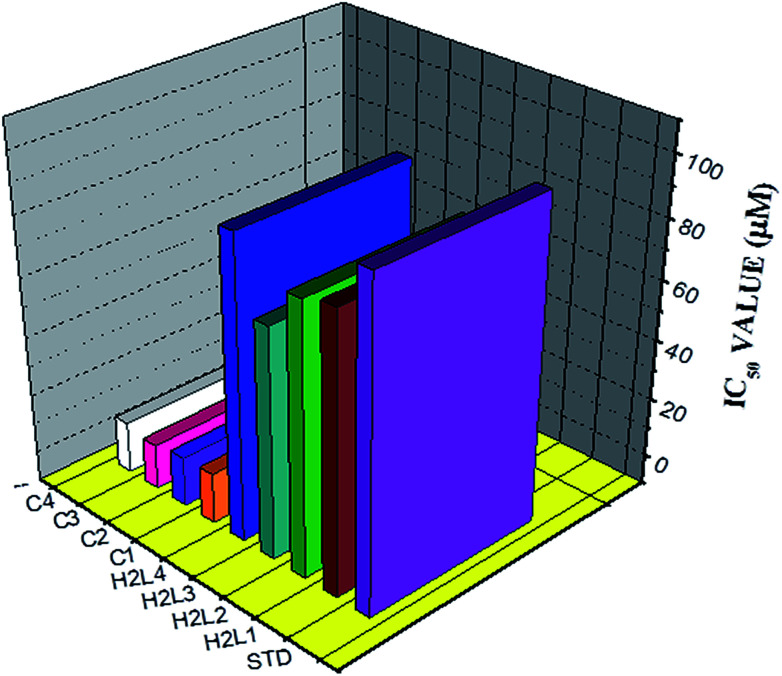
The DPPH radical scavenging activity of the ligands, [RuHClCO(PPh_3_)_3_] and new Ru(ii) complexes.

Since the synthesized ligands and Ru(ii) complexes showed good DNA binding affinity, it is considered worthwhile to investigate their antioxidant activity. It has been reported that free radical species such as reactive oxygen species (ROS), are involved in the pathogenesis of various diseases through effects on DNA directly and by acting as a tumour promoter.^59,60^ The DPPH free radical scavenging activity of the ligands and complexes were studied and quantitatively antioxidant properties were determined by using phosphomolybdate method. We knew that the inhibitory effects of the tested compounds on DPPH radical are concentration dependent and the suppression ratio increases with increasing sample concentrations (Fig. S36†). As seen from Fig. 17, the IC_50_ values of the ligands H_2_L^1^, H_2_L^2^, H_2_L^3^, H_2_L^4^, complex 1, complex 2, complex 3, complex 4 and vitamin C (standard) are 83.17 ± 1.50, 80.75 ± 1.34, 67.28 ± 1.44, 91.21 ± 1.54, 7.13 ± 0.23, 6.75 ± 0.18, 5.28 ± 0.24, 7.39 ± 0.14 and 98.72 ± 1.50 respectively. From the results, it is revealed that the complexes exhibited good radical scavenging activity over the standard ascorbic acid and ligands. In the phosphomolybdenum assay, the antioxidant activity is expressed as the number of equivalents of ascorbic acid (Table 6). The total antioxidant activity of the compounds is in the following order complex 3 > complex 2 > complex 1 > complex 4 > H_2_L^3^ > H_2_L^2^ > H_2_L^1^ > H_2_L^4^ > ascorbic acid > [RuHClCO(PPh_3_)_3_]. The results concluded that the metal complexes are better antioxidants than their parent ligands which may be due to the chelation of the ligands to metal ion.^35^ The radical scavenging ability of the new complexes is greater than that of few other reported ruthenium complexes, containing Schiff base ligands.^9^

**Table 6:** Estimation of Total antioxidant capacity of ligands, [RuHClCO(PPh_3_)_3_] and new Ru(ii) complexes (1–4)

Compounds	μg ascorbic acid equivalents/ml
H_2_L^1^	33.98 ± 0.29
H_2_L^2^	37.10 ± 0.43
H_2_L^3^	40.01 ± 0.27
H_2_L^4^	32.90 ± 0.57
[RuHClCO(PPh_3_)_3_]	07.02 ± 0.08
Complex 1	54.13 ± 0.28
Complex 2	58.27 ± 0.46
Complex 3	62.71 ± 0.37
Complex 4	54.99 ± 0.65

#### Antimicrobial studies

**Fig. 18:**
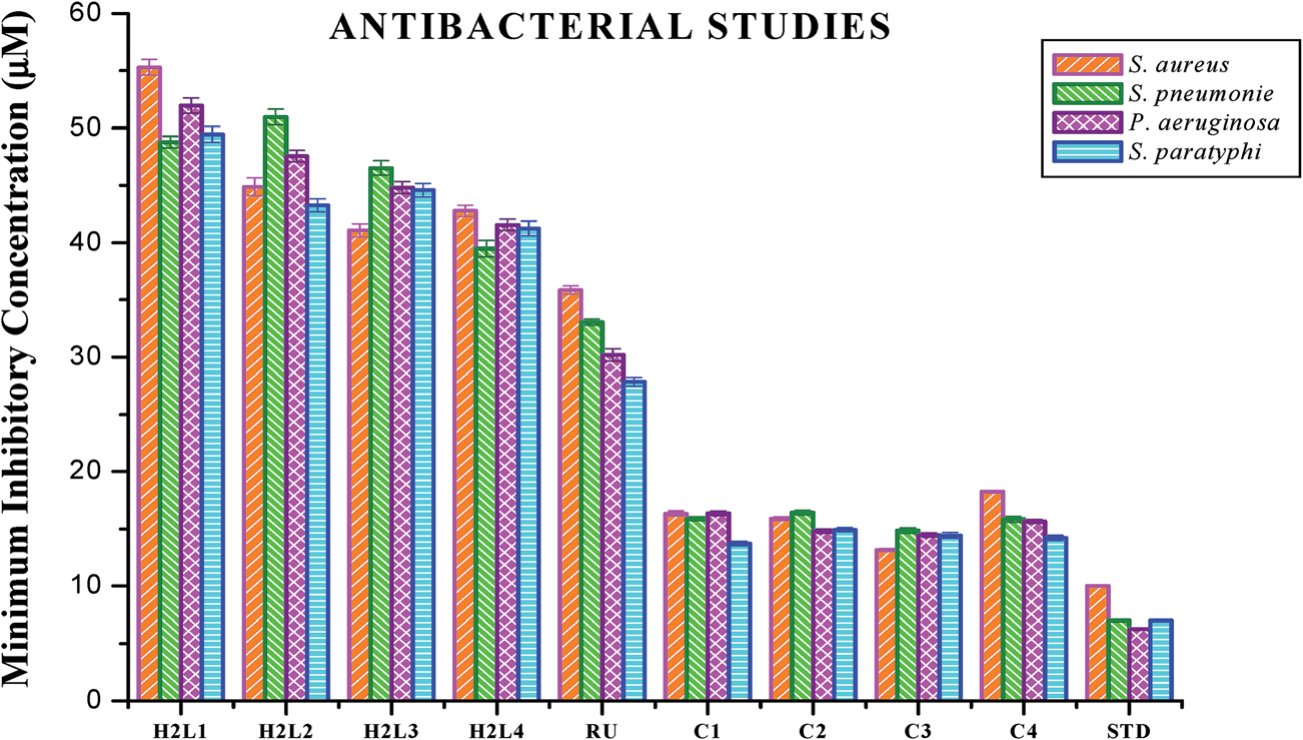
Antibacterial activity of ligands H_2_L^1–4^, [RuHClCO(PPh_3_)_3_] and new Ru(ii) complexes (1–4). Error bars represent the standard deviation of the mean (*n* = 3).

**Fig. 19:**
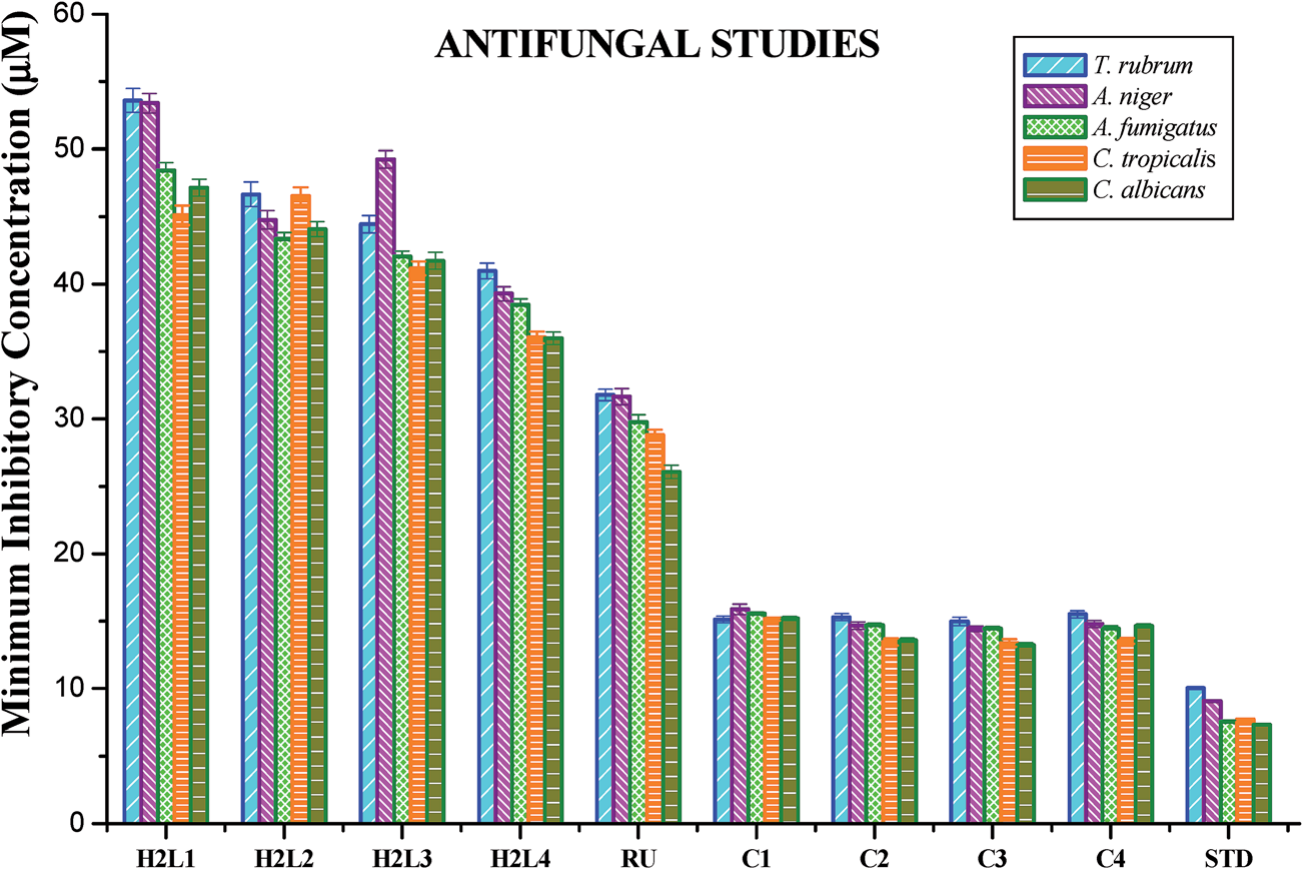
Antifungal activity of ligands H_2_L^1–4^, [RuHClCO(PPh_3_)_3_] and new Ru(ii) complexes (1–4). Error bars represent the standard deviation of the mean (*n* = 3).

The free ligands and their cyclometallated ruthenium(ii) carbonyl complexes were screened for their *in vitro* antimicrobial activity against certain pathogenic bacterial and fungal species at three different concentrations using disc diffusion method. The test solutions were prepared in 10% aqueous DMSO and the results of the antimicrobial activities are expressed as the zone of inhibition and minimum inhibitory concentration (MIC) and are given in Tables S4–S7 in the ESI†, Fig. 18 and 19. From the results, we concluded that the ligands and complexes exhibited significant activity, but they did not reach the effectiveness of the conventional bacteriocide gentamicin and fungicide ketoconazole. Tested complexes had better antimicrobial activity than the ligands against all pathogens. This may be explained by Tweedy's chelation theory.^61^ Coordination of ligands reduce the polarity of the metal ion essentially by partial sharing of its positive charge with the donor groups within the chelate ring system formed during the coordination and leading to the increase in lipophilic nature of the central metal atom, which favours the effective permeation through the lipid layer of microorganism.^61,62^ On comparing the antifungal activity of the complexes, complex 3 was more active on four fungi namely *Aspergillus niger*, *Aspergillus fumigatus*, *Candida tropicalis* and *Candida albicans* followed by complex 2, complex 4 and complex 1. When tested against *T. rubrum*, the activity of the complexes in the order of 3 > 2 > 1 > 4. In antibacterial studies, the complex 3 was effective against *S. aureus* followed by complex 2, 1 and 4 and in the case of *S. pneumonie* the activity of the complexes follows the order of 3 > 1 > 4 > 2. On comparing the activity of the complexes, complexes 2 and 3 were more active on bacteria namely *P. aeruginosa*. When tested against *S. paratyphi* complex 1 stood out as good followed by the complexes 4, 3 and 2 respectively. The compounds showed different degrees of antimicrobial activity due to the structural variations of themselves and variation on the group of microorganisms.^63^ In addition, antimicrobial activity of the complexes was compared with already reported ruthenium complexes, showing that the new Ru(ii) complexes exhibited better activity.^64,65^

#### Anticancer studies

**Fig. 20:**
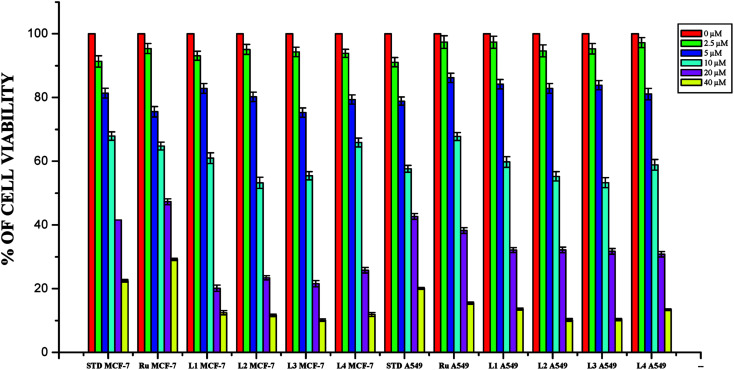
The newly synthesized ligands, [RuHClCO(PPh_3_)_3_] and cisplatin inhibit MCF-7 and A549 cells proliferation in a dose dependent manner. MCF-7 and A549 cells were treated with different concentrations of ligands for 48 h, the cell viability was determined and the results were expressed as percentage cell viability with control. Results shown are mean, which are three separate experiments performed in triplicate.

**Fig. 21:**
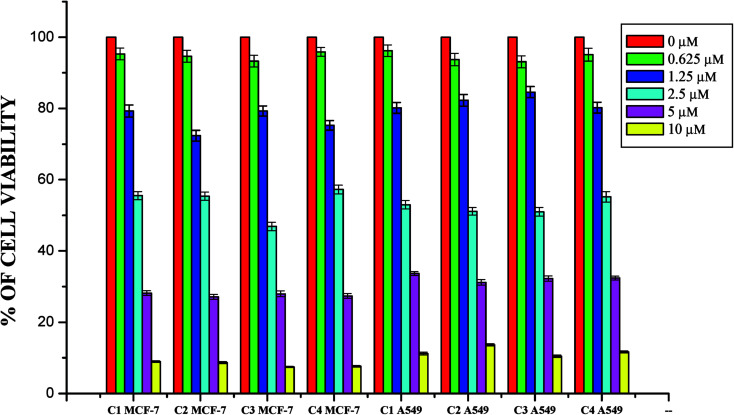
The newly synthesized ruthenium(ii) complexes (1–4) inhibit MCF-7 and A549 cells proliferation in a dose dependent manner. MCF-7 and A549 cells were treated with different concentrations of complexes for 48 h, the cell viability was determined and the results were expressed as percentage cell viability with control. Results shown are mean, which are three separate experiments performed in triplicate.

**Fig. 22:**
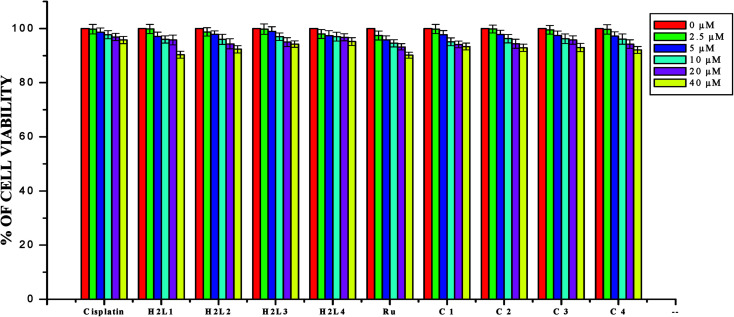
The newly synthesized ligands H_2_L^1–4^, Ru(ii) complexes, [RuHClCO(PPh_3_)_3_] and cisplatin inhibit HaCaT cells proliferation in a dose dependent manner. HaCaT cells were treated with different concentrations of compounds for 48 h, the cell viability was determined and the results were expressed as percentage cell viability with control. Results shown are mean, which are three separate experiments performed in triplicate.

The DNA/protein binding studies, antioxidant and antimicrobial studies had shown that the ligands and ruthenium(ii) complexes studied here have therapeutic potentials and were subjected to study their anticancer activity. A number of coumarin derivatives have shown considerable anticancer activity against a number of cell lines. All the 3-acetyl-7-methoxy-coumarin-4(*N*)-substituted thiosemicarbazone ligands, metal precursor [RuHClCO(PPh_3_)_3_] and their new cyclometallated Ru(ii) complexes were assessed for their cytotoxicity with two human derived cell lines namely human lung carcinoma (A549) and human breast cancer cells (MCF-7) by using MTT assay. For comparison purpose, cisplatin was used as a positive control under identical conditions. The dose–response curves are given in Fig. 20–22 and the results are shown in Table 7. The IC_50_ values for the ligands and their complexes for the MCF-7 and A549 showed that the ligands and their Ru(ii) complexes were cytotoxic to these cells.

**Table 7:** The IC_50_ values for the human breast cancer cell line MCF-7, human lung carcinoma cancer cell line A549 and human normal keratinocyte cells (HaCaT) with the ligands H_2_L^1–4^, [RuHClCO(PPh_3_)_3_] and new organometallic Ru(ii) complexes for 48 h

Compounds	IC_50_ values (μM)		
	MCF-7	A549	HaCaT
Cisplatin	16.79 ± 0.08	15.10 ± 0.05	>40
H_2_L^1^	13.06 ± 0.29	12.64 ± 0.17	>40
H_2_L^2^	12.12 ± 0.32	12.12 ± 0.16	>40
H_2_L^3^	11.27 ± 0.21	11.63 ± 0.15	>40
H_2_L^4^	13.11 ± 0.25	13.83 ± 0.18	>40
[RuHClCO(PPh_3_)_3_]	20.10 ± 0.18	15.96 ± 0.21	>40
Complex 1	2.86 ± 0.17	2.96 ± 0.07	>40
Complex 2	2.62 ± 0.07	2.93 ± 0.07	>40
Complex 3	2.53 ± 0.10	2.37 ± 0.04	>40
Complex 4	3.02 ± 0.05	3.05 ± 0.12	>40

Whilst most of the Schiff bases did not display good anticancer activity, the coumarin appended thiosemicarbazones H_2_L^1–4^ against MCF-7 cell line exhibited IC_50_ values of 13.06 ± 0.29 μM, 12.12 ± 0.32 μM, 11.27 ± 0.21 μM and 13.11 ± 0.25 μM respectively, which were lower than that of cisplatin, indicating ligands showed good activity over cisplatin. Against the human breast cancer cell line MCF-7, the antiproliferative activity of Ru(ii) complexes 1–4 was higher than that of their parent ligands and cisplatin with lower IC_50_ values of 2.86 ± 0.17 for complex 1, 2.62 ± 0.07 for complex 2, 2.53 ± 0.10 for complex 3 and 3.02 ± 0.05 for complex 4.

The ligands H_2_L^1–4^ and complexes exhibited high cytotoxic effects on lung cancer cells with low IC_50_ values indicating their efficiency in killing cancer cells even at low concentrations. In A549, the anticancer activity of the compounds follows the order ruthenium precursor (15.96 ± 0.21) < cisplatin (15.10 ± 0.05) < H_2_L^4^ (13.83 ± 0.18) < H_2_L^1^ (12.64 ± 0.24) < H_2_L^2^ (12.12 ± 0.16) < H_2_L^3^ (11.63 ± 0.15) < complex 4 (3.05 ± 0.12) < complex 1 (2.96 ± 0.07) < complex 2 (2.93 ± 0.07) < complex 3 (2.37 ± 0.04).

From this study, we concluded that the coumarin-appended Schiff base derivatives had potent activity than cisplatin used to treat human breast cancer and human lung cancer. In addition, the coordination of the ligands to the Ru(ii) ion increases the anticancer activity of the complexes to six times greater than the ligands and eight times greater than the cisplatin against both the cell lines. Thus, the presence of the substituent at N-terminal nitrogen seems to be important for varying the order of activity of the compounds. In both MCF-7 and A549 cell lines, ruthenium(ii) complex 3 containing more electron donating ethyl group at N-terminal nitrogen exhibited high activity followed by complex 2 (NH–Me), complex 1 (NH–H) and complex 4, which has electron withdrawing phenyl group at terminal nitrogen atom of the ligand. On the basis of the results, the antiproliferative activity of these compounds has been arranged in the order 3 > 2 > 1 > 4 > H_2_L^3^ > H_2_L^2^ > H_2_L^1^ > H_2_L^4^. Interestingly, this observation is in agreement with their previous biological studies, suggesting that the anticancer activities of the tested compounds against cancer cell lines may be related to their ability to intercalate the base pairs of the DNA and/or their free radical scavenging activity.

In order to investigate the selectivity of the compounds for cancer cells rather than normal cell lines, the compounds were also screened for their anticancer activity on the human normal keratinocyte cells (HaCaT). In the noncancerous cell line, all the compounds showed their nontoxic nature. Furthermore, the IC_50_ values exhibited by the complexes showed a higher cytotoxic effect when compared to the other reported Ru(ii) complexes.^24,26,30,31,44,66,67^

#### Lactate dehydrogenase release

**Fig. 23:**
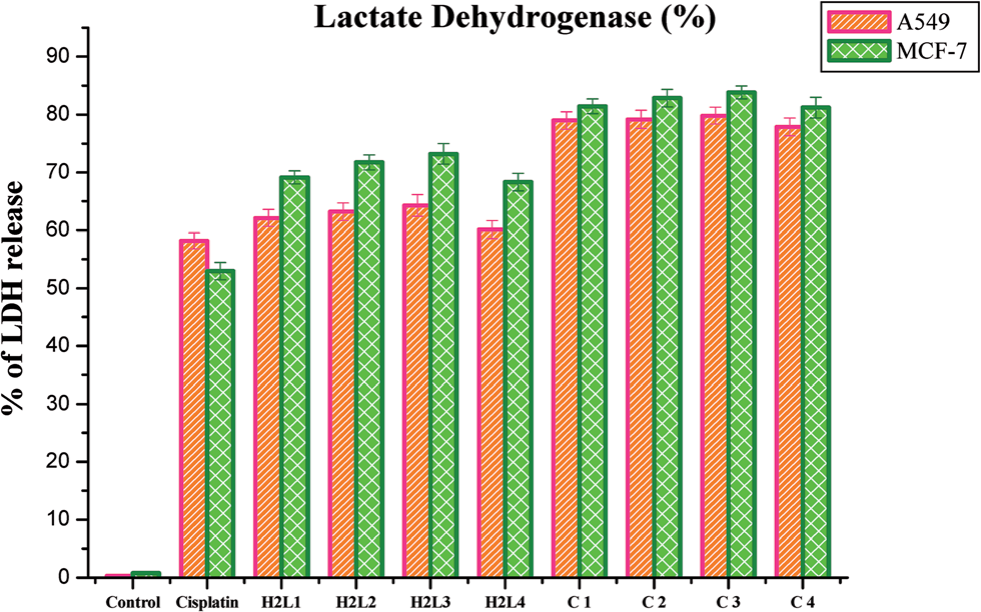
Percentage of lactate dehydrogenase released by the human cancer cell lines A549 and MCF-7 after an incubation period of 48 h with ligands H_2_L^1–4^ and complexes 1–4. Error bars represent the standard mean error (*n* = 6).

LDH is a stable cytoplasmic enzyme that is released into the culture medium following loss of membrane integrity and serves as a general mean to assess cytotoxicity resulting from chemical compounds or environmental toxic factors.^68^ In the present study, LDH leakage into the culture medium of the compounds treated A549 and MCF-7 cells was analyzed. It was observed that the new ligands and their Ru(ii) complexes could potently induce the release of LDH into the culture medium of A549 and MCF-7 cells when they are treated with their respective IC_50_ concentrations for 48 h, indicating that the compounds could rupture the plasma membrane (Fig. 23). The results confirmed the cytotoxic effect of the ligands and complexes on lung and breast cancer cell lines. The compounds could induce LDH leakage as high as that of cisplatin. The induction of LDH release was found to be higher for complexes than their parent ligands when comparing among them and with the control. Among the compounds examined, complex 3 was found to be more potent in inducing LDH leakage into the culture than the rest. These results are comparable with the earlier reports.^30^

#### Nitric oxide release

**Fig. 24:**
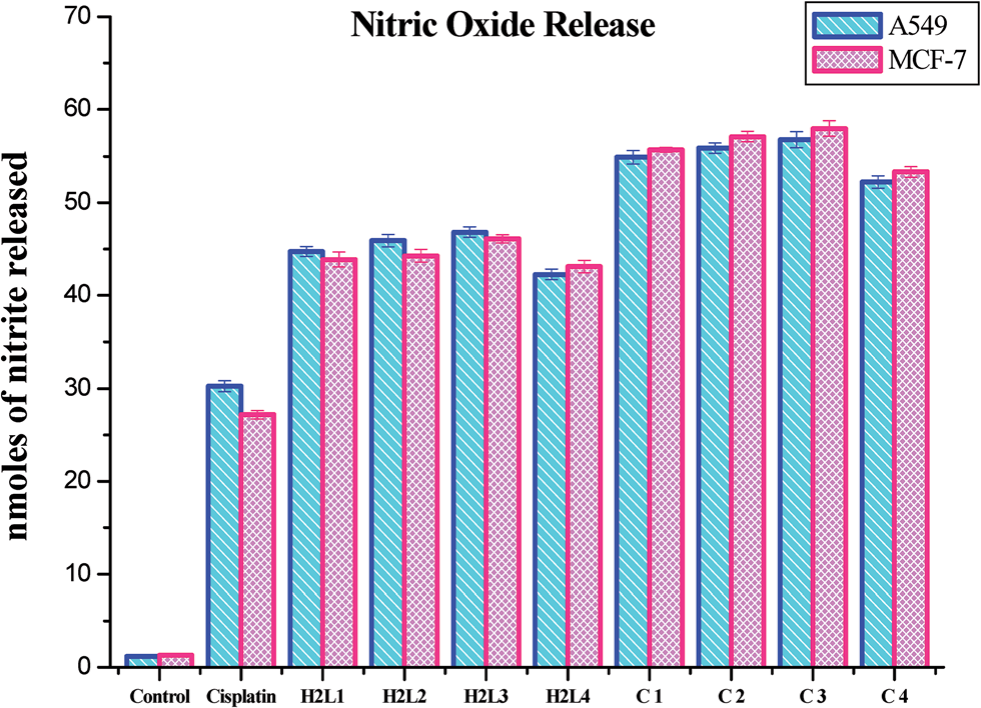
Nitrite released (nmoles) by the human cancer cell lines A549 and MCF-7 after an incubation period of 48 h with ligands H_2_L^1–4^ and complexes 1–4. Error bars represent the standard mean error (*n* = 6).

Nitrite is the stable product of the nitric oxide released in response to oxidative stress. The amount of nitrite in the culture medium corresponds to the level of nitric oxide. Hence the level of nitrite is estimated to measure the NO produced after complex treatment. The level of nitrite was found to increase significantly in the ligands and Ru(ii) complexes treated A549 and MCF-7 cells compared to the control. The increased level of nitrite in the cell culture medium further confirms the cytotoxic effects of the presently studied compounds. The induction of cytotoxicity in terms of NO release in A549 and MCF-7 cells follows the order of 3 > 2 > 1 > 4 > H_2_L^3^ > H_2_L^2^ > H_2_L^1^ > H_2_L^4^ > cisplatin. These results authenticated the results obtained by MTT and LDH leakage assays indicating that complex 3 is more effective than the remaining three complexes (Fig. 24). The nitric oxide release by the compounds is higher than that of cisplatin, and it is better than those reported for other ruthenium(ii) complexes containing triphenylphosphines.^30^ The results of the nitric oxide assay support the concept that the complex-induced cell death is mediated by reactive oxygen species generation.

## Conclusion

A series of ligands 3-acetyl-7-methoxycoumarin-4*N*-substituted thiosemicarbazones were prepared and consecutively made to undergo complexation with ruthenium precursor [RuHCl(CO)(PPh_3_)_3_]. The reactions ended up in cyclometallated organometallic Ru(ii) complexes. Analytical and spectral studies accounted for the formation of the complexes. The ligands acted in a tridendate manner by bonding through C, N and S atoms. A systematic study on their DNA/protein binding properties and antioxidant activities was carried out. Experimental results suggest that the ligands and complexes can bind to DNA *via* an intercalation mode and a static quenching with proteins. Evaluation of their inhibitory potency against bacterial and fungal pathogens revealed that the compounds possess a good spectrum of antimicrobial activity. The *in vitro* cytotoxic activity of the complexes was ascertained *via* MTT assay and the IC_50_ values were found in the range of 2.53 ± 0.10–3.02 ± 0.05 μM and 2.37 ± 0.04–3.05 ± 0.12 μM for MCF-7 and A549 cancerous cell lines, respectively. Moreover, LDH and NO release assays confirm the anticancer potential of the tested compounds. The results validated that complex 3 is a potent chemotherapeutic drug among others. This may be attributed to the more electron donating ability of the N-terminal ethyl group. In addition, the present investigation lights up the potent antiproliferative effect of ruthenium complexes by inhibiting the viability of A549 and MCF-7 cells. Hence, further studies on animal models to elucidate the clear mechanism of action of the complexes are highly warranted to unveil the ruthenium complexes as anticancer drugs.

## Experimental section

### Materials and methods

All the reagents used were of analytically or chemically pure grade. Solvents were purified and dried according to standard procedures.^69^ The metal precursor [RuHCl(CO)(PPh_3_)_3_]^70^ and 3-acetyl-7-methoxy-chromene-2-one (3-acetyl-7-methoxy coumarin)^71^ were prepared according to the literature procedure. Doubly distilled water was used to prepare buffers. Ethidium bromide (EB), serum albumins (BSA/HSA), calf thymus DNA (CT-DNA) and 3-(4,5-dimethyl thiazol-2-yl)-2,5-diphenyltetrazolium bromide (MTT) were purchased from HiMedia (Mumbai, India) and used as received. Human lung cancer cell lines A549, human breast cancer cell lines MCF-7 and human normal keratinocyte cells (HaCaT) were obtained from the National Center for Cell Science (NCCS), Pune, India. Melting points were measured in a Lab India apparatus. Infrared spectra were measured as KBr pellets on a JASCO FT-IR 4100 instrument between 400–4000 cm^−1^. Elemental analysis of carbon, hydrogen, nitrogen and sulfur was determined by using Vario EL III CHNS at the Department of Chemistry, Bharathiar University, Coimbatore, India. The electronic spectra of the compounds were recorded with a JASCO V-630 spectrophotometer using DMSO as the solvent in 800–200 nm range. Emission spectra were recorded by using JASCO FP 6600 Spectrofluorimeter. ^1^H NMR spectra were recorded in DMSO at room temperature with a Bruker 400 MHz instrument, chemical shift relative to tetramethylsilane. The stability of the compounds was performed in 1% aqueous DMSO and phosphate buffer–DMSO (99 : 1). The stability was analyzed by monitoring the electronic spectra over 24 h at room temperature on a JASCO 4100 spectrophotometer.

### X-ray crystallography

Suitable single crystals for the ligands H_2_L^1–3^ and complexes (1, 2 and 4) were obtained from methanol and dichloromethane/methanol medium respectively. Single crystal data collections and corrections for the ligands (H_2_L^1–3^) and new Ru(ii) complexes (1, 2 and 4) were carried out with a Gemini Xcaliber Atlas four circle diffractometer using graphite monochromated Mo Kα (*λ* = 0.71073 Å) radiation at 295 K. All the calculations were done by using SHELXS-200, SHELXL-2015/7 and Olex-2 programs.^72^

### Preparation of 3-acetyl-7-methoxy-2*H*-chromen-2-one^71^

An ethanolic solution (10 cm^3^) of 4-methoxysalicylaldehyde (1.22 g, 1 mmol) was taken along with the catalytic amount of piperidine and ethylacetoacetate (1.95 g, mmol) and was refluxed for 5 h with continuous stirring. The reaction mixture was then cooled to room temperature, which afforded yellow precipitate. The crude product was filtered, washed with ethanol (3 × 10 cm^3^) and recrystallized from ethanol to yield an yellow crystalline product. Yield = 89%. Mp 118–120 °C; anal. calcd for C_12_H_10_O_4_: C, 66.11; H, 4.63; found: C, 66.08; H, 4.61; UV-Vis (DMSO), *λ*_max_ (*ε*): 354 (26 868) nm (dm^3^ mol^−1^ cm^−1^); IR (*ν*, cm^−1^): *ν*(C=O lactone) 1731, *ν*(C=O acetyl group) 1681. ^1^H NMR (400 MHz, DMSO-d_6_, *δ* ppm, *J* Hz): *δ* 8.628 (s, 1H, C4–H), *δ* 7.857–7.877 (d, *J* = 8, 1H, C8–H), *δ* 6.996–7.057 (m, 2H, C5–H and C6–H), *δ* 3.894 (s, 3H, −OCH_3_), *δ* 2.573 (s, 3H, −CH_3_).

### Synthesis of ((1*E*)-1-(1-(7-methoxy-2-oxo-2*H*-chromen-3-yl)ethylidene) thiosemicarbazone) [H_2_-7MAC-tsc] (H_2_L^1^)^71^

Thiosemicarbazide (0.417 g, 4.58 mmol) was dissolved in 30 cm^3^ of methanol with continuous stirring and gently heated for a period of 30 min. This was added to a methanolic solution (20 cm^3^) of 3-acetyl-7-methoxy-2*H*-chromen-2-one (1 g, 4.58 mmol). To this, few drops of glacial acetic acid were added and the mixture was refluxed for 2 h with continuous stirring. The mixture was then cooled to room temperature whereby a yellow crystalline compound precipitated. This was collected by filtration, washed well with cold methanol and dried under vacuum. The compound was recrystallized from DMF–methanol (1 : 9 v/v). Yellow colored fine single crystals suitable for X-ray analysis were collected. Yield: 72%. Mp: 213 °C. Anal. calcd for C_13_H_13_N_3_O_3_S: C, 53.58; H, 4.50; N, 14.42; S, 11.00. Found: C, 53.56; H, 4.47; N, 14.41; S, 11.00%. FT-IR (*ν*, cm^−1^) in KBr: *ν*(C=O lactone) 1728, *ν*(C=N) 1644, *ν*(–NH_2_) 3279, *ν*(–NH) 3136, *ν*(C=S) 831. UV-Vis (DMSO), *λ*_max_ (*ε*): 276 (20 943) nm (dm^3^ mol^−1^ cm^−1^); 354 (30 488) nm (dm^3^ mol^−1^ cm^−1^). ^1^H NMR (400 MHz, DMSO-d_6_, *δ* ppm, *J* Hz): *δ* 7.986 (s, 1H, C4–H), *δ* 7.719–7.740 (d, 1H, *J* = 8.4, C5–H), 7.039–7.094 (m, 2H, Ar–H), *δ* 3.931 (s, 3H, –OCH_3_), *δ* 2.302 (s, 3H, –CH_3_), *δ* 10.424 (s, 1H, NH–C=S), *δ* 8.477 & 8.409 (2 br s, 2H, –NH_2_).

The very similar method was followed to synthesize the following compounds.

### Synthesis of ((1*E*)-1-(1-(7-methoxy-2-oxo-2*H*-chromen-3-yl)ethylidene) 4(*N*)-methyl thiosemicarbazone) [H_2_-7MAC-mtsc] (H_2_L^2^)

The ligand [H_2_-7MAC-mtsc] was prepared from 4-(*N*)-methylthiosemicarbazide (0.481 g, 4.58 mmol) and 3-acetyl-7-methoxy-2*H*-chromen-2-one (1 g, 4.58 mmol) in the presence of glacial acetic acid. Single crystals suitable for X-ray diffraction studies were obtained by recrystallisation of ligand H_2_L^2^ in methanol. Yield: 73%. Mp: 117 °C anal. calcd for C_14_H_15_N_3_O_3_S: C, 55.05; H, 4.96; N, 13.76; S, 10.49. Found: C, 55.03; H, 4.93; N, 13.71; S, 10.48%. FT-IR (*ν*, cm^−1^) in KBr: *ν*(C=O lactone) 1714, *ν*(C=N) 1608, *ν*(terminal –NH) 3262, *ν*(–NH) 3210, *ν*(C=S) 834. UV-Vis (DMSO), *λ*_max_ (*ε*): 275 (24 094) nm (dm^3^ mol^−1^ cm^−1^); 352 (33 647) nm (dm^3^ mol^−1^ cm^−1^). ^1^H NMR (400 MHz, DMSO-d_6_, *δ* ppm, *J* Hz): *δ* 8.314 (s, 1H, C4–H), *δ* 7.684–7.705 (d, *J* = 8.4, 1H, C5–H), 6.985–7.029 (m, 2H, Ar–H), *δ* 3.879 (s, 3H, –OCH_3_), *δ* 2.244 (s, 3H, –CH_3_), *δ* 10.377 (s, 1H, NH–C=S), *δ* 8.465–8.490 (q, 1H, terminal –NH), *δ* 3.027–3.037 (d, *J* = 4, 1H, terminal –NH–CH_3_).

### Synthesis of ((1*E*)-1-(1-(7-methoxy-2-oxo-2*H*-chromen-3-yl)ethylidene) 4(*N*)-ethyl thiosemicarbazone) [H_2_-7MAC-etsc] (H_2_L^3^)

The ligand [H_2_-7MAC-etsc] was prepared from 4-(*N*)-ethylthiosemicarbazide (0.546 g, 4.58 mmol) and 3-acetyl-7-methoxy-2*H*-chromen-2-one (1 g, 4.58 mmol) in the presence of glacial acetic acid. The compound was recrystallised by using methanol to yield suitable yellow crystals for X-ray analysis. Yield: 76%. Mp: 174 °C. Anal. calcd for C_15_H_17_N_3_O_3_S: C, 56.40; H, 5.37; N, 13.15; S, 10.05. Found: C, 56.37; H, 5.34; N, 13.11; S, 10.01%. FT-IR (*ν*, cm^−1^) in KBr: *ν*(C=O lactone) 1725, *ν*(C=N) 1606, *ν*(terminal –NH) 3241, *ν*(–NH) 3155, *ν*(C=S) 835. UV-Vis (DMSO), *λ*_max_ (*ε*): 276 (14 245) nm (dm^3^ mol^−1^ cm^−1^); 353 (21 388) nm (dm^3^ mol^−1^ cm^−1^). ^1^H NMR (400 MHz, DMSO-d_6_, *δ* ppm, *J* Hz): *δ* 8.291 (s, 1H, C4–H), *δ* 7.694–7.715 (d, *J* = 8.4, 1H, C5–H), *δ* 7.679–7.034 (m, 2H, Ar–H), *δ* 3.881 (s, 3H, –OCH_3_), *δ* 2.247 (s, 3H, –CH_3_), *δ* 10.269 (s, 1H, NH–C=S), *δ* 8.472–8.499 (t, *J* = 5.6, 1H, terminal –NH), *δ* 3.593–3.622 (p, 2H, terminal –NH–CH_2_), *δ* 1.136–1.172 (t, *J* = 7.2, 3H, –CH_3_).

### Synthesis of ((1*E*)-1-(1-(7-methoxy-2-oxo-2*H*-chromen-3-yl)ethylidene) 4(*N*)-phenyl thiosemicarbazone) [H_2_-7MAC-ptsc] (H_2_L^4^)

The ligand [H_2_-7MAC-ptsc] was prepared from 4-(*N*)-phenylthiosemicarbazide (0.766 g, 4.58 mmol) and 3-acetyl-7-methoxy-2*H*-chromen-2-one (1 g, 4.58 mmol) in the presence of glacial acetic acid. The compound was recrystallised by using methanol. Yield: 77%. Mp: 186 °C. Anal. calcd for C_19_H_17_N_3_O_3_S: C, 62.10; H, 4.67; N, 11.43; S, 8.72. Found: C, 62.07; H, 4.64; N, 11.4; S, 8.69%. FT-IR (*ν*, cm^−1^) in KBr: *ν*(C=O lactone) 1700, *ν*(C=N) 1600, *ν*(terminal –NH) 3241, *ν*(–NH) 3114, *ν*(C=S) 826. UV-Vis (DMSO), *λ*_max_ (*ε*): 282 (27 646) nm (dm^3^ mol^−1^ cm^−1^); 356 (41 701) nm (dm^3^ mol^−1^ cm^−1^). ^1^H NMR (400 MHz, DMSO-d_6_, *δ* ppm, *J* Hz): *δ* 8.458 (s, 1H, C4–H), *δ* 6.691–7.524 (m, 8H, Ar–H), *δ* 3.885 (s, 3H, –OCH_3_), *δ* 2.332 (s, 3H, –CH_3_), *δ* 10.777 (s, 1H, NH–C=S), *δ* 10.126 (s, 1H, terminal –NH).

### Synthesis of new ruthenium(ii) complexes

#### Synthesis of [Ru(7MAC-tsc)(CO)(PPh_3_)_2_] (1)

A solution of [H_2_-7MAC-tsc] (0.021 g; 0.105 mmol) in 10 cm^3^ of benzene was added dropwise to a boiling solution of [RuHCl(CO)(PPh_3_)_3_] (0.100 g, 0.105 mmol) in benzene and refluxed for 7 h and allowed to stand for 4 days at room temperature. Reddish orange solid formed was filtered, washed with petroleum ether (60–80 °C) and crystallized from dichloromethane and methanol mixture (1 : 1 v/v) to yield red transparent needle like crystals suitable for X-ray analysis. Yield: 66%. Mp: 247 °C. Anal. calcd for C_50_H_41_N_3_O_4_P_2_RuS: C, 63.67; H, 4.39; N, 4.45; S, 3.39. Found: C, 63.64; H, 4.37; N, 4.42; S, 3.37%. FT-IR (*ν*, cm^−1^) in KBr: *ν*(C=O lactone) 1685, *ν*(C=N) 1607, *ν*(C–S) 745, *ν*(C≡O) 1918, 1436, 1090, 696 (for PPh_3_). UV-Vis (DMSO), *λ*_max_ (*ε*): 337 (33 654) nm (dm^3^ mol^−1^ cm^−1^) (LMCT s → d). ^1^H NMR (400 MHz, DMSO-d_6_, *δ* ppm, *J* Hz): *δ* 7.150–7.547 (m, 31H, Ar–H), *δ* 6.290–6.444 (m, 2H, C6–H and C8–H), *δ* 3.842 (s, 3H, –OCH_3_), *δ* 1.977 (s, 3H, –CH_3_), *δ* 5.651 (br s, 2H, –NH_2_).

A similar method was followed to synthesize other ruthenium(ii) complexes.

#### Synthesis of [Ru(7MAC-mtsc)(CO)(PPh_3_)_2_] (2)

Complex 2 was prepared by the same procedure as described for 1, with H_2_L^2^ (0.105 mmol) as a ligand. Needle shaped, transparent pink colour crystals were obtained on slow evaporation of the reaction mixture. Yield: 62%. Mp >300 °C. Anal. calcd for C_51_H_43_N_3_O_4_P_2_RuS: C, 64.00; H, 4.45; N, 4.39; S, 3.34. Found: C, 63.97; H, 4.43; N, 4.36; S, 3.30%. FT-IR (*ν*, cm^−1^) in KBr: *ν*(C=O lactone) 1680, *ν*(C=N) 1606, *ν*(C–S) 744, *ν*(C≡O) 1925, 1404, 1087, 695 (for PPh_3_). UV-Vis (DMSO), *λ*_max_ (*ε*): 268 (45 067) nm (dm^3^ mol^−1^ cm^−1^) (intraligand transition); 363 (20 485) nm (dm^3^ mol^−1^ cm^−1^) (LMCT s → d). ^1^H NMR (400 MHz, DMSO-d_6_, *δ* ppm, *J* Hz): *δ* 7.139–7.637 (m, 31H, Ar–H), *δ* 6.62–6.63 (m, 1H, C8–H), *δ* 6.87–6.90 (dd, 1H, *J* = 2.4, 8.8, C6–H), *δ* 3.842 (s, 3H, –OCH_3_), *δ* 2.098 (s, 3H, –CH_3_), *δ* 5.972–6.098 (q, 1H, terminal –NH), *δ* 1.221 (s, 3H, –NH–CH_3_).

#### Synthesis of [Ru(7MAC-etsc)(CO)(PPh_3_)_2_] (3)

Complex 3 was prepared by the same procedure as described for 1 with H_2_L^3^ (0.105 mmol) as a ligand. Red solid formed was filtered, washed with petroleum ether (60–80 °C) and crystallized from dichloromethane and methanol to yield orange crystals. Yield: 62%. Mp 237 °C. Anal. calcd for C_52_H_45_N_3_O_4_P_2_RuS: C, 64.31; H, 4.68; N, 4.32; S, 3.30. Found: C, 63.29; H, 4.64; N, 4.29; S, 3.28%. FT-IR (*ν*, cm^−1^) in KBr: *ν*(C=O lactone) 1681, *ν*(C=N) 1606, *ν*(C–S) 745, *ν*(C≡O) 1923, 1432, 1087, 695 (for PPh_3_). UV-Vis (DMSO), *λ*_max_ (*ε*): 268 (16 367) nm (dm^3^ mol^−1^ cm^−1^) (intraligand transition); 357 (38 703) nm (dm^3^ mol^−1^ cm^−1^) (LMCT s → d). ^1^H NMR (400 MHz, DMSO-d_6_, *δ* ppm, *J* Hz): *δ* 7.216–7.634 (m, 31H, Ar–H), *δ* 6.644–6.921 (m, 2H, C6–H and C8–H), *δ* 3.774 (s, 3H, –OCH_3_), *δ* 1.887 (s, 3H, –CH_3_), *δ* 6.18–6.23 (t, 1H, terminal –NH), *δ* 1.126–1.288 (m, 2H, *J* = 4.8, –NH–CH_2_), *δ* 0.701–0.737 (t, 3H, *J* = 7.2, –CH_2_–CH_3_).

#### Synthesis of [Ru(7MAC-ptsc)(CO)(PPh_3_)_2_] (4)

Complex 4 was prepared by the same procedure as described for 1 with H_2_L^4^ (0.105 mmol) as a ligand and [RuHCl(CO)(PPh_3_)_3_] (0.105 mmol). Red solid formed was filtered, washed with petroleum ether (60–80 °C) and crystallized from dichloromethane and methanol to yield pink crystals. Yield: 60%. Mp 230 °C. Anal. calcd for C_56_H_45_N_3_O_4_P_2_RuS: C, 65.99; H, 4.45; N, 4.12; S, 3.14. Found: C, 65.96; H, 4.42; N, 4.09; S, 3.12%. FT-IR (*ν*, cm^−1^) in KBr: *ν*(C=O lactone) 1681, *ν*(C=N) 1606, *ν*(C–S) 744, *ν*(C≡O) 1920, 1431, 1089, 694 (for PPh_3_). UV-Vis (DMSO), *λ*_max_ (*ε*): 278 (19 763) nm (dm^3^ mol^−1^ cm^−1^) (intraligand transition); 365 (10 531) nm (dm^3^ mol^−1^ cm^−1^) (LMCT s → d), 380 (10 424) nm (dm^3^ mol^−1^ cm^−1^) (LMCT s → d). ^1^H NMR (400 MHz, DMSO-d_6_, *δ* ppm, *J* Hz): *δ* 6.909–7.324 (m, 36H, Ar–H), *δ* 6.635–6.757 (m, 2H, C6–H & C8–H), *δ* 3.793 (s, 3H, –OCH_3_), *δ* 1.957 (s, 3H, –CH_3_), *δ* 8.488 (s, 1H, terminal –NH).

### Biomolecular interaction studies

The stability of the compounds was performed in 1% aqueous DMSO and phosphate buffer : DMSO (99 : 1). The stability was analyzed by monitoring the electronic spectra for the period of 24 h at room temperature on a JASCO 4100 spectrophotometer. DNA binding studies, EB-displacement assays, DNA viscosity studies, DNA cleavage experiments and protein binding studies have been done according to the reported methods.^30,31,35,44,73^ The detailed procedures for these experiments are provided in the ESI.†

### *In vitro* antioxidant assays

The DPPH radical scavenging activity of the compounds have been carried out according to the reported method.^74^ In this study, various concentrations of the experimental standard ascorbic acid, [RuHCl(CO)(PPh_3_)_3_], ligands (20–100 μM) and complexes (2–10 μM) in methanol were taken. Total antioxidant activity of the compounds was determined by the phosphomolybdate method.^75^

### *In vitro* antimicrobial assay

Antimicrobial activities of [RuHClCO(PPh_3_)_3_], ligands H_2_L^1–4^ and new organometallic Ru(ii) complexes (1–4) were evaluated by agar well diffusion method^76^ as reported, by taking *Staphylococcus aureus*, *Streptococcus pneumonie*, *Pseudomonas aeruginosa*, *Salmonella paratyphi* and fungus such as *Candida albicans*, *Trichophyton rubrum*, *Aspergillus niger*, *Aspergillus fumigatus* and *Candida tropicalis*. The above said all test organisms were obtained from the MTCC, Chandigarh, India and Microbiological laboratory, Coimbatore, Tamil Nadu, India. The antimicrobial activity of the test compounds was checked with various concentrations (25 μg ml^−1^, 50 μg ml^−1^ and 100 μg ml^−1^) against all the test pathogens. Gentamicin and ketoconazole were used as positive controls to study the antibacterial and antifungal activities respectively. Each experiment was performed in triplicate and the results are represented as average zone of inhibition and minimum inhibitory concentration of all the test pathogens.

### Cytotoxicity studies

Cytotoxic activity of the compounds was tested with human lung cancer cell lines A549, human breast cancer cell lines MCF-7 and human normal keratinocyte cells (HaCaT) by using MTT assay, which was done according to the earlier literature methods^77^ and IC_50_ values obtained from nonlinear regression using GraphPad Prism 5.^78^ The LDH release^79^ and NO release^80^ assays of the compounds was evaluated by the earlier reported methods.

## Conflicts of interest

There are no conflicts to declare.

## Supplementary Material

RA-008-C7RA12104K-s001

RA-008-C7RA12104K-s002
