# Supplementary material for: An investigation on 3-acetyl-7-methoxy-coumarin Schiff bases and their Ru(ii) metallates with potent antiproliferative activity and enhanced LDH and NO release
Source: RSC Adv. 2018 Jan 4;8(3):1539–61. doi: 10.1039/c7ra12104k (PMC9077138; doi:10.1039/c7ra12104k)
Supplement: RA-008-C7RA12104K-s001 [file RA-008-C7RA12104K-s001.pdf]

Electronic Supplementary Material (ESI) for RSC Advances.
This journal is © The Royal Society of Chemistry 2017

Supplementary Information

Experimental procedure

DNA binding study

All the experiments involving CT-DNA binding were carried out in deionised water with tris(hydroxymethyl)-aminomethane (Tris, 5 mM) and sodium chloride (50 mM) and adjusted to pH 7.2 with hydrochloric acid at room temperature. The concentration of CT-DNA was determined by UV absorbance at 260 nm. Solutions of CT-DNA in Tris-HCl buffer gave a ratio of UV absorbance at 260 and 280 nm, A_{260}/A_{280}, of approximately 1.9, indicating that the DNA was sufficiently free of protein. The molar absorption coefficient, ε_{260}, was taken as 6600 M^{-1} cm^{-1}.Various concentrations of CT-DNA (2-2.5 μM) was added to the complexes (25 μM dissolved in DMSO/Tris-HCl buffer, 1 % DMSO in the final solution). While measuring the absorption spectra, an equal amount of DNA was added to both the test and reference solutions to eliminate the absorbance of DNA itself. Control experiments with DMSO were performed and no change in the spectra of CT-DNA was observed. Absorption spectra were recorded after equilibrium at 20 °C for 10 min. The intrinsic binding constant K_b was determined by using following equation(1)

$$[DNA]/[\varepsilon_a-\varepsilon_f]) = [DNA]/[\varepsilon_b-\varepsilon_f]+1/K_b[\varepsilon_b-\varepsilon_f] \qquad (S1)$$

The absorption coefficients ε_a, ε_f and ε_b correspond to A_{obsd}/[DNA], the extinction coefficient for the free compound and for the compound in the fully bound form respectively. The slope and the intercept of the linear fit of the plot of $[DNA]/[\varepsilon_a - \varepsilon_f]$ versus [DNA] give $1/[\varepsilon_a - \varepsilon_f]$ and $1/K_b[\varepsilon_b - \varepsilon_f]$ respectively. The intrinsic binding constant K_b can be obtained from the ratio of the slope to the intercept. In order to find out the mode of attachment of CT DNA to the compounds, fluorescence quenching experiments of EB-DNA were carried out by adding our complexes to the Tris-HCl buffer of EB-DNA. The change in the fluorescence intensity was recorded. Before measurements, the system was shaken well and incubated at room temperature for 5 min. The emission was recorded at 530–750 nm.

Viscosity studies

Viscosity experiments were carried out using a semi-microviscometer maintained at 27 °C in a thermostatic water bath. The DNA concentration was maintained at 100μM, while the

compound concentration was varied from 0 to 100 μM. For each sample, the flow time was measured as triplicate and the average flow time was calculated. The values of relative specific viscosity $(\eta/\eta_0)^{1/3}$ were plotted against 1/R (1/R = [compound]/[DNA]), where η and η_0 correspond to the relative viscosity of DNA in the presence of the complex and the relative viscosity of DNA alone. The relative viscosity (η_0) values were calculated from the observed flow time of the DNA solution (t) corrected for the flow time of the buffer alone (t_0), using the expression $\eta_0 = (t-t_0)/t_0$.

DNA Cleavage Experiment

The cleavage of DNA was monitored using agarose gel electrophoresis. Supercoiled pBR322 DNA (100 ng) in 5 % DMSO and 95 % Tris buffer (5 mM, pH 7.2) with 50 mM NaCl was incubated at 37 °C in the absence and presence of compounds. The DNA, compound and sufficient buffer were premixed in a vial, and the reaction was allowed to proceed for 2 h at 37 °C. The samples were then analyzed by 1.5 % agarose gel electrophoresis in Tris−acetic acid−ethylenediamine tetraacetic acid buffer. The gel was stained with 0.5 μg cm^{-3} ethidium bromide before migration. After electrophoresis at 50 V for 3 h, the gel was illuminated and the digital images were analyzed by gel documentation system (SYNGEN USA).

Serum albumin binding study

Bovine Serum Albumin (BSA) and human serum albumin were purchased from Hi Media, The protein binding study was performed by tryptophan fluorescence quenching experiments using bovine serum albumin (BSA, 10 μM) or human serum albumin (HSA, 10 μM) as the substrate in phosphate buffer (pH= 7.2). Quenching of the emission intensity of tryptophan residues of BSA at 346 nm (excitation wavelength at 280 nm)/ HSA at 345 nm (excitation wavelength at 290 nm) was monitored using ligands and complexes as quenchers with increasing concentration (10-100μM). Synchronous fluorescence spectra of BSA or HSA with various concentrations of the complexes were obtained from 300 to 400 nm when $\Delta\lambda$ = 60 nm and from 290 to 500 nm when $\Delta\lambda$ = 15 nm. For synchronous fluorescence spectra, the same concentrations of serum albumins and the compounds were also used and the spectra were measured at two different $\Delta\lambda$ values (difference between the excitation and emission wavelengths of BSA), such as 15 and 60 nm. Fluorescence and synchronous measurements were performed using a 1 cm quartz cell on a JASCO FP 6600 spectrofluorimeter.

The quenching data can be analyzed according to the Stern-Volmer equation,

$$I_o/I_{corr} = K_{SV}[Q]+1 \qquad \text{Eq (S2)}$$

where I_o is the emission intensity in the absence of compound, I_{corr} is the corrected emission intensity in the presence of compound, K_{SV} is the quenching constant and [Q] is the concentration of the compound.

In order to correct the inner filter effect, the following equation used

$$Icorr = Iobs*10^{(Aexc+Aem)/2}$$

where I_{corr} is the corrected fluorescence value, I_{obs} the measured fluorescence value, A_{exc} is the absorption value at the excitation wavelength, and A_{em} the absorption value at the emission wavelength.

The equilibrium binding constant and the number of binding sites can be analyzed by using Scatchard equation

$$\log [(F_o-F)/F]= \log K_b + n \log [Q] \qquad \text{Eq (S3)}$$

where, F_o and F are the corrected emission intensities of serum albumins in the absence and presence of the compounds, where n is the binding site per albumin and K_b is the binding constant.

Table S1. Selected bond lengths (Å) and bond angles (°) of the ligands $\mathbf{H_2L^{1\text{-}3}}$

BOND LENGTHS					
$\mathbf{H_2L^1}$		$\mathbf{H_2L^2}$		$\mathbf{H_2L^3}$	
S1 C1	1.696(3)	S1 C1	1.677(3)	S1 C1	1.671(4)
O1 C5	1.380(3)	O1 C6	1.377(3)	O1 C7	1.384(5)
O1 C6	1.371(3)	O1 C7	1.374(2)	O1 C8	1.366(4)
O2 C5	1.203(3)	O2 C6	1.210(2)	O2 C7	1.197(5)
O3 C8	1.358(5)	O3 C9	1.351(4)	O3 C10	1.358(6)
O3 C13	1.433(5)	O3 C14	1.436(4)	O3 C15	1.440(7)
N1 H1	0.810(4)	N1 H1	0.840(3)	N1 H1	0.960(6)
N1 C1	1.320(5)	N1 C1	1.321(3)	N1 C1	1.324(6)
N2 N3	1.375(4)	N2 N3	1.374(3)	N2 N3	1.364(5)
N2 C1	1.346(4)	N2 C1	1.357(3)	N2 C1	1.367(5)
N2 H2	0.840(4)	N2 H2	0.850(2)	N2 H2	0.860(6)
N3 C2	1.289(3)	N3 C3	1.285(3)	N3 C4	1.273(5)
C2 C3	1.501(4)	C3 C4	1.488(3)	C4 C5	1.499(6)
C2 C4	1.475(4)	C3 C5	1.477(4)	C4 C6	1.481(6)
C4 C5	1.456(3)	C5 C6	1.463(3)	C6 C7	1.481(6)
C3 H3	0.960(4)	C4 H4	0.960(3)	C5 H5	0.960(6)
C4 C9	1.3907(7)	C5 C13	1.356(3)	C6 C14	1.353(5)
C4 C12	1.363(3)	C7 C12	1.386(3)	C13 C14	1.432(6)
C4 C12	1.363(3)	C10 H10	0.930(2)	C9 H9	0.929(4)
C10 H10	0.930(3)	C7 C8	1.379(4)	C8 C9	1.383(6)
C6 C7	1.378(4)	C8 C9	1.381(3)	C8 C13	1.390(6)
C7 C8	1.395(4)	C8 H8	0.930(2)	C14 H14	0.929(4)
C9 H9	0.930(3)	C9 C10	1.401(4)	C9 C10	1.383(5)
C9 C10	1.370(6)	C10 C11	1.363(4)	C10 C11	1.403(7)
C10 C11	1.408(4)	C11 C12	1.363(4)	C11 C12	1.362(7)
C11 C12	1.412(4)	C11 H11	1.408(3)	C11 H11	0.930(4)
C7 H7	0.930(2)	C13H13	0.930(2)	C12H12	0.931(4)
C12H12	0.930(2)	C12 C13	1.414(4)	C12 C13	1.398(5)
C8 C9	1.390(4)	N1 C2	1.455(3)	N1 C2	1.461(5)
				C2 C3	1.497(8)
**BOND ANGLES**					
$\mathbf{H_2L^1}$		$\mathbf{H_2L^2}$		$\mathbf{H_2L^4}$	
C5 O1 C6	122.8(2)	C6 O1 C7	122.8(2)	C7 O1 C8	123.7(3)
C8 O3 C13	118.3(3)	C9 O3 C14	118.1(2)	C10 O3 C15	117.5(4)
C1 N1 N2	117.4(3)	C1 N1 N2	115.7(2)	C1 N1 H1	111.0(3)
C1 N1 H1B	116.0(3)	C1 N1 H1	116.0(2)	C1 N1 N2	114.7(4)
C1 N1 H1A	122.0(3)	C1 N1 C2	124.3(2)	C1 N1 C2	124.2(4)
N2 N3 H2	125.0(2)	N2 N3 H2	121.0(2)	N2 N3 H2	129.0(4)
N2 C1 H2	116.0(2)	N2 C1 H2	118.0(2)	N2 C1 H2	115.0(4)
O1 C5 O2	115.5(2)	O1 C6 O2	115.5(2)	O1 C7 O2	114.7(4)
O1 C5 C4	117.1(2)	O1 C5 C6	117.5(2)	O1 C7 C6	116.6(4)
O2 C5 C4	127.5(3)	O2 C6 C5	127.0(2)	O2 C7 C6	128.7(4)
C5 C4 C12	118.7(2)	C6 C5 C13	118.0(2)	C7 C6 C14	118.4(4)

C4 C12 C11	122.1(2)	C5 C13 C12	122.7(2)	C6 C13 C14	123.3(4)
C5 C4 C2	119.8(2)	C6 C5 C3	119.6(2)	C4 C6 C7	119.9(4)
C6 C11 C0	116.8(3)	C7 C12 C11	116.5(2)	C8 C12 C13	117.6(4)
C6 C11 C12	118.4(2)	C7 C12 C13	118.4(2)	C8 C13 C14	117.2(4)
C11 C10 C9	121.1(3)	C10 C11 C12	121.2(2)	C13 C11 C12	121.8(4)
C8 C9 C10	120.1(3)	C9 C10 C11	120.5(2)	C12 C10 C11	119.3(4)
C7 C8 C9	120.8(3)	C8 C9 C10	119.9(2)	C11 C9 C10	120.4(4)
O3 C8 C7	123.5(3)	O3 C9 C8	124.4(2)	O3 C9 C10	124.8(4)
O3 C8 C9	115.6(3)	O3 C9 C10	115.7(2)	O3 C10 C11	114.8(4)
C7 C8 C6	117.6(3)	C7 C8 C9	118.3(2)	C10 C8 C9	118.7(4)
O1 C6 C11	119.8(2)	O1 C7 C12	119.8(2)	O1 C8 C13	120.8(4)
O1 C6 C7	116.7(2)	O1 C7 C8	116.6(2)	O1 C9 C8	117.0(4)
C7 C6 C11	123.5(2)	C8 C7 C12	123.6(2)	C9 C8 C13	122.2(4)
N3 C2 C4	114.6(2)	N3 C3 C5	115.6(2)	N3 C4 C6	115.5(4)
N3 C2 C3	124.2(3)	N3 C3 C4	123.3(2)	N3 C4 C5	125.3(4)
C2 C3 C4	121.2(2)	C5 C3 C4	121.0(2)	C5 C6 C4	119.3(4)
N1 C1 N2	117.4(3)	N1 C1 N2	115.7(2)	N1 C1 N2	115.7(2)

Table S2. Hydrogen bonds for ligands **H_2L^1**, **H_2L^2, H_2L^3** and Complexes **2** and **4** [Å and °]

D–H···A	d(D–H)	d(H···A)	d(D···A)	<(DHA)
[**H_2-7MAC-tsc**] (**H_2L^1**)				
O(1)…H(1)-N(1)	0.812	2.628	3.070	50.42
N(1)-H(1)...O(1)	0.812	2.628	3.070	50.42
Symmetry operation: (**x, y, z); (-x, -y, -z);**				
[**H_2-7MAC-mtsc**] (**H_2L^2**)				
O(1) …N(3)			3.013	
N(3) …O(1)			3.013	
Symmetry operation: (**x, y, z)**; (**-x, x-y, z**); (**-x+y, -x, z**); (**-x, -y, -z)**; (**y, -x+y, -z**);(**x-y,x,-z**);				
[**H_2-7MAC-ptsc**] (**H_2L^3**)				
O(2) …N(3)			2.653	
Symmetry operation: (**x, y, z); (-x, -y, -z);**				
Complex **2**				
O(1) …O(1)			2.954	
Symmetry operation: (**x, y, z); (-x, -y, -z);**				
Complex **4**				
O(1) …O(1)			3.004	
Symmetry operation: (**x, y, z); (-x, -y, -z);**				

Table S3. Selected bond lengths (Å) and bond angles (°) of the complexes (**1, 2** and **4**)

**BOND LENGTHS**					
**1**		**2**		**4**	
Ru1 C5	2.072(2)	Ru1 C5	2.077(4)	Ru1 C13	2.062(2)
Ru1 C1	1.846(2)	Ru1 C1	1.853(4)	Ru1 C1	1.848(2)
Ru1 N1	2.0970(2)	Ru1 N1	2.083(4)	Ru1 N1	2.077(2)
Ru1 P1	2.3794(8)	Ru1 P1	2.365(1)	Ru1 P1	2.3768(8)
Ru1 P2	2.3765(8)	Ru1 P2	2.383(1)	Ru1 P2	2.3762(9)
Ru1 S1	2.4580(8)	Ru1 S1	2.447(1)	Ru1 S1	2.4422(8)
**BOND ANGLES**					
**1**		**2**		**4**	
S1 Ru1 C1	102.54(8)	S1 Ru1 C1	103.2(1)	S1 Ru1 C1	102.20(8)
S1 Ru1 C5	156.97(7)	S1 Ru1 C5	157.0(1)	S1 Ru1 C13	157.56(7)
S1 Ru1 N1	78.39(6)	S1 Ru1 N1	78.9(1)	S1 Ru1 N1	79.38(6)
S1 Ru1 P1	88.05(2)	S1 Ru1 P1	85.02(4)	S1 Ru1 P1	87.05(2)
S1 Ru1 P2	89.09(2)	S1 Ru1 P2	88.34(4)	S1 Ru1 P2	87.97(2)
P1 Ru1 C1	89.41(8)	P1 Ru1 C1	88.3(1)	P1 Ru1 C1	90.17(8)
P1 Ru1 N1	90.25(6)	P1 Ru1 N1	93.3(1)	P1 Ru1 N1	90.94(6)
P1 Ru1 P2	174.83(3)	P1 Ru1 P2	172.32(4)	P1 Ru1 P2	174.70(3)
P1 Ru1 C5	92.42(7)	P1 Ru1 C5	97.1(1)	P1 Ru1 C13	93.42(7)
P2 Ru1 C1	87.00(8)	P2 Ru1 C5	90.5(1)	P2 Ru1 C13	91.88(7)
P2 Ru1 C5	91.90(7)	P2 Ru1 C1	89.5(1)	P2 Ru1 C1	89.10(8)
P2 Ru1 N1	93.40(6)	P2 Ru1 N1	89.2(1)	P2 Ru1 N1	89.94(6)
N1 Ru1 C1	179.0(1)	N1 Ru1 C1	177.5(2)	N1 Ru1 C1	178.1(1)
N1 Ru1 C5	78.58(8)	N1 Ru1 C5	78.1(2)	N1 Ru1 C13	78.18(9)
C1 Ru1 C5	100.5(1)	C1 Ru1 C5	99.8(2)	C1 Ru1 C13	100.2(1)
C2 S1 Ru1	96.03(9)	C2 S1 Ru1	95.9(2)	C2 S1 Ru1	95.12(9)
C15 P1 Ru1	116.98(9)	C16 P1 Ru1	113.4(2)	C33 P1 Ru1	114.17(9)
C21 P1 Ru1	118.37(9)	C22 P1 Ru1	116.3(2)	C21 P1 Ru1	112.76(9)
C27 P1 Ru1	111.21(9)	C28 P1 Ru1	114.9(2)	C27 P1 Ru1	116.68(9)
C33 P2 Ru1	112.79(9)	C34 P2 Ru1	112.6(2)	C39 P2 Ru1	116.68(9)
C39 P2 Ru1	106.54(9)	C40 P2 Ru1	117.8(2)	C45 P2 Ru1	112.12(9)
C45 P2 Ru1	115.52(9)	C46 P2 Ru1	115.9(2)	C51 P2 Ru1	117.1(9)
N2 N1 Ru1	125.6(2)	N2 N1 Ru1	125.6(3)	N2 N1 Ru1	124.8(2)
C3 N1 Ru1	117.8(2)	C3 N1 Ru1	118.4(3)	C3 N1 Ru1	118.7(2)
C4 C5 Ru1	112.1(2)	C5 C6 Ru1	132.2(3)	C5 C13 Ru1	112.7(2)
C5 C6 Ru1	132.3(2)	C4 C5 Ru1	112.6(3)	C12 C13 Ru1	131.8(2)
O1 C1 Ru1	177.7(2)	O1 C1 Ru1	178.7(4)	O1 C1 Ru1	177.4(2)

Table S4. Antibacterial results of Schiff base ligands $\mathbf{H_2L^{1-4}}$, $[RuHClCO(PPh_3)_3]$ and new Ru(II) complexes (**1-4**)

Compounds	Concentration (µg/ml)	Zone of inhibition (mm) against bacteria			
		S. aureus	*S. pneumonie*	*P. aeruginosa*	*S. paratyphi*
$\mathbf{H_2L^1}$	25	-	-	-	-
	50	13.01±0.23	12.38±0.12	13.22±0.41	13.25±0.77
	100	17.19±0.16	16.41±0.31	18.15±0.24	16.54±0.46
$\mathbf{H_2L^2}$	25	-	-	-	-
	50	12.11±0.25	12.14±0.33	12.56±0.37	13.43±0.12
	100	17.52±0.28	17.31±0.41	17.66±0.45	16.24±0.57
$\mathbf{H_2L^3}$	25	-	-	-	-
	50	12.31±0.41	11.21±0.15	12.51±0.34	12.22±0.25
	100	15.61±0.65	17.21±0.21	16.41±0.23	17.15±0.32
$\mathbf{H_2L^4}$	25	-	-	-	-
	50	15.41±0.52	12.22±0.43	12.13±0.32	12.23±0.51
	100	18.46±0.73	18.14±0.22	17.46±0.65	16.44±0.51
Complex **1**	25	-	-	-	-
	50	12.16±0.11	12.41±0.36	13.23±0.32	12.55±0.04
	100	18.09±0.15	18.51±0.94	16.66±0.44	18.95±0.31
Complex **2**	25	-	-	-	-
	50	13.12±0.41	13.21±0.13	12.12±0.13	13.41±0.01
	100	18.65±0.13	17.14±0.12	17.95±0.21	18.45±0.51
Complex **3**	25	-	-	-	-
	50	12.04±0.60	11.11±0.22	13.23±0.52	12.53±0.14
	100	17.14±0.12	17.46±0.41	17.34±0.45	17.44±0.27
Complex **4**	25	-	-	-	-
	50	12.21±0.42	12.31±0.48	12.33±0.53	12.31±0.38
	100	18.11±0.11	16.54±0.12	16.85±0.62	17.55±0.54
Metal precursor	25	-	-	-	-
	50	15.19±0.08	14.39±0.34	15.78±0.44	13.29±0.65
	100	17.67±0.78	16.44±0.11	17.89±0.42	17.44±0.55
Gentamicin	25	20.32±0.43	20.32±0.39	20.22±0.22	20.39±0.44

Table S5. Antifungal results of Schiff base ligands $\mathbf{H_2L^{1\text{-}4}}$, $[RuHClCO(PPh_3)_3]$ and Ru(II) complexes (**1-4**)

Compounds	Concentration (µg/ml)	Zone of inhibition (mm) against fungus				
		Trichophyton rubrum	*Aspergillus niger*	*Aspergillus fumigatus*	*Candida tropicalis*	*C. albicans*
$\mathbf{H_2L^1}$	25		-			
	50	13.15±0.16	13.13±0.33	11.23±0.73	11.93±0.01	11.24±0.14
	100	17.34±0.51	17.41±0.14	17.43±0.22	17.43±0.51	17.02±0.91
$\mathbf{H_2L^2}$	25					
	50	11.34±0.17	12.45±0.33	11.81±0.41	11.53±0.75	13.38±0.12
	100	17.43±0.51	16.61±0.21	18.32±0.22	17.14±0.46	18.17±0.15
$\mathbf{H_2L^3}$	25	-	-			
	50	13.56±0.05	12.55±0.31	12.24±0.51	12.35±0.51	11.29±0.12
	100	19.13±0.21	17.62±0.12	18.23±0.24	17.24±0.51	18.19±0.22
$\mathbf{H_2L^4}$	25					
	50	12.36±0.32	12.51±0.83	11.33±0.33	12.24±0.23	12.29±0.21
	100	16.35±0.66	17.31±0.21	18.22±0.14	19.22±0.32	17.19±0.15
Complex **1**	25	-	-		-	-
	50	11.57±0.22	11.74±0.26	12.31±0.62	12.82±0.34	12.22±0.41
	100	17.56±0.13	17.52±0.41	19.64±0.65	18.51±0.54	18.91±0.41
Complex **2**	25	-			-	
	50	12.16±0.15	11.56±0.24	12.61±0.71	12.64±0.15	12.45±0.31
	100	17.45±0.35	19.63±0.12	17.36±0.45	19.52±0.27	16.28±0.10
Complex **3**	25	-	-			
	50	13.25±0.52	12.73±0.55	13.53±0.13	13.55±0.34	12.29±0.15
		17.44±0.61	18.43±0.12	18.31±0.21	17.91±0.31	17.73±0.21
Complex **4**	25	-	-			
	50	12.41±0.56	11.55±0.26	12.36±0.52	12.43±0.12	12.49±0.14
	100	18.42±0.18	17.66±0.13	18.15±0.45	17.13±0.57	17.19±0.66
Metal precursor	25	-	-	-	-	-
	50	14.18±0.54	13.19±0.34	15.58±0.65	14.78±0.43	13.29±0.45
	100	17.91±0.24	15.14±0.31	18.38±0.65	18.59±0.41	16.67±0.11
Ketaconazole	25	24.12±0.16	21.89±0.11	23.01±0.25	19.48±0.23	20.34±0.20

Table S6. Minimum inhibitory concentration (MIC) in μM of the antibacterial studies

COMPOUNDS	IC_{50} VALUES (μM)			
	S. aureus	*S. pneumonie*	*P. aeruginosa*	*S. paratyphi*
Gentamicin	10.00±0.03	7.01±0.09	6.27±0.06	7.00±0.08
H_2L^1	55.29±0.69	48.77±0.09	51.96±0.67	49.46±0.67
H_2L^2	44.89±0.78	50.95±0.09	47.58±0.51	43.26±0.56
H_2L^3	41.07±0.56	46.54±0.09	44.82±0.53	44.60±0.59
H_2L^4	42.78±0.49	39.48±0.09	41.54±0.49	41.34±0.63
$[RuHClCO(PPh_3)_3]$	35.84±0.37	33.05±0.28	30.19±0.53	27.85±0.38
Complex 1	16.35±0.21	15.86±0.07	16.37±0.19	13.71±0.19
Complex 2	15.90±0.16	16.43±0.09	14.81±0.19	14.94±0.21
Complex 3	13.14±0.12	14.85±0.09	14.46±0.18	14.43±0.25
Complex 4	18.25±0.11	15.89±0.12	15.66±0.16	14.21±0.23

Table S7: Minimum inhibitory concentration (MIC) in (μM) of the antifungal studies

COMPOUNDS	IC_{50} VALUES (μM)				
	Trichophyton rubrum	*Aspergillus niger*	*Aspergillus fumigatus*	*Candida tropicalis*	*C. albicans*
Ketaconazole	10.03±0.09	9.05±0.08	7.56±0.08	7.70±0.10	7.30±0.09
H_2L^1	53.58±0.89	53.40±0.67	48.42±0.59	45.10±0.73	47.15±0.62
H_2L^2	46.66±0.91	44.76±0.72	43.35±0.39	46.53±0.64	44.07±0.56
H_2L^3	44.44±0.63	49.26±0.63	42.06±0.37	41.15±0.52	41.72±0.62
H_2L^4	40.97±0.57	39.28±0.54	38.47±0.43	36.02±0.43	35.97±0.47
$[RuHClCO(PPh_3)_3]$	31.76±0.42	31.63±0.59	29.75±0.54	28.79±0.41	26.09±0.48
Complex 1	15.29±0.29	15.89±0.18	15.56±0.11	15.15±0.11	15.19±0.15
Complex 2	15.14±0.24	14.67±0.31	14.70±0.1	13.46±0.15	13.59±0.15
Complex 3	14.97±0.31	14.44±0.21	14.44±0.27	13.37±0.16	13.24±0.16
Complex 4	15.52±0.27	14.80±0.25	14.45±0.13	13.54±0.21	14.65±0.13

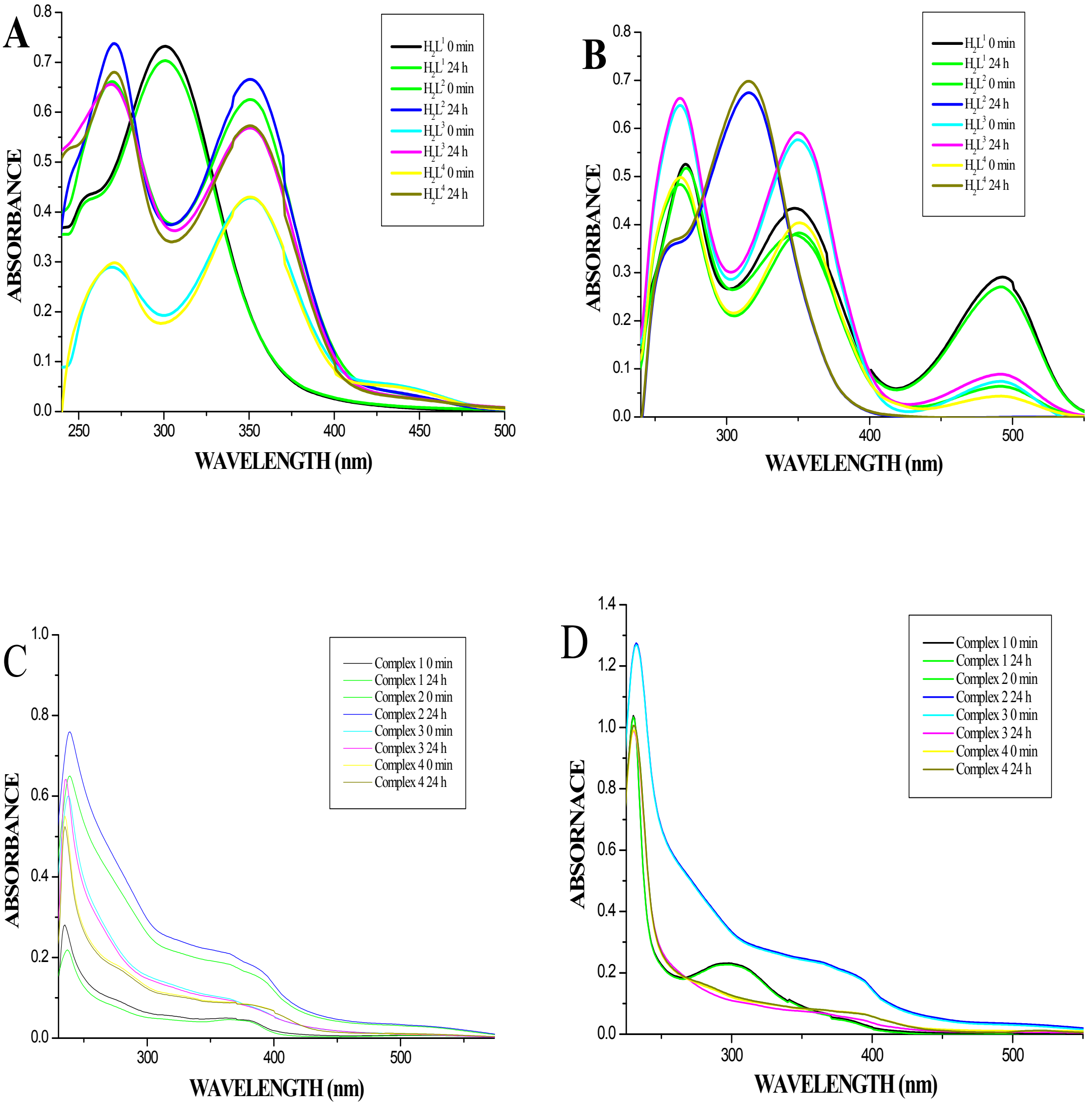

Fig. S1. Stability studies of the complexes using UV-Vis absorption spectroscopic technique. A) Absorption spectra ligands in 1% aqueous DMSO; B) absorption spectra ligands in 99: 1 phosphate buffer: DMSO; C) absorption spectra complexes in 1% aqueous DMSO; D) absorption spectra complexes in 99:1 phosphate buffer: DMSO

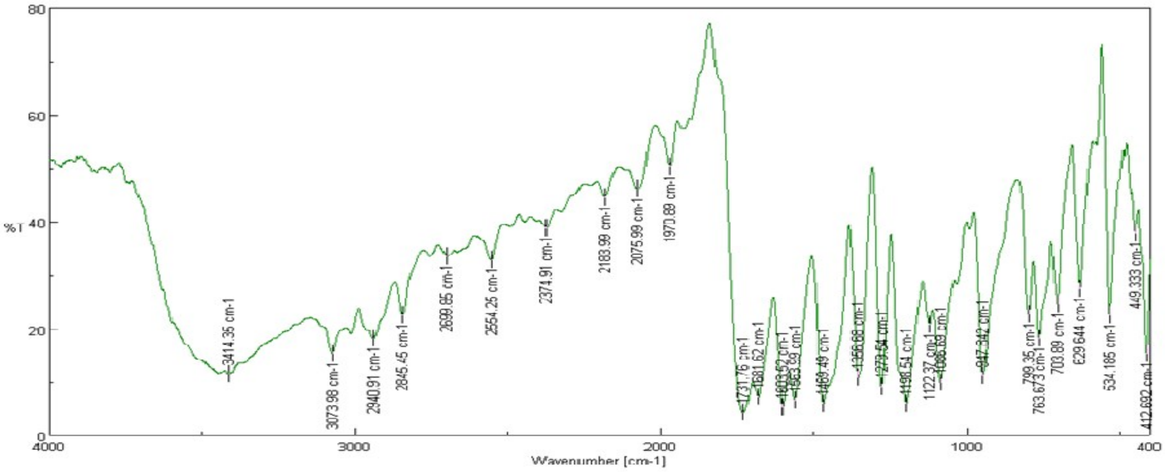

Fig. S2. IR spectrum of 3-acetyl-7-methoxy coumarin

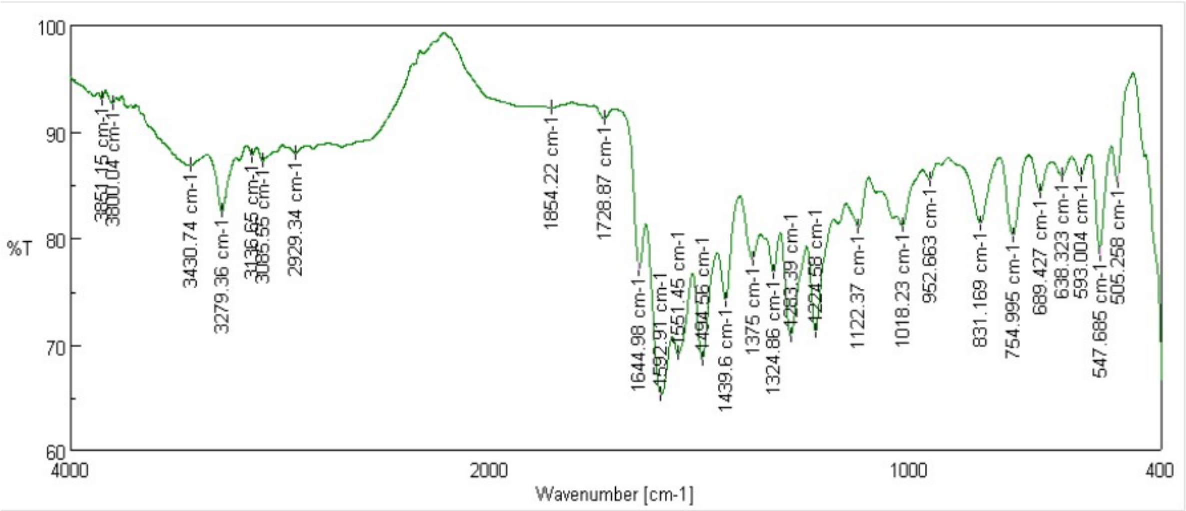

Fig. S3. IR spectrum of **[H_2-7MAC-tsc]** (**H_2L^1**)

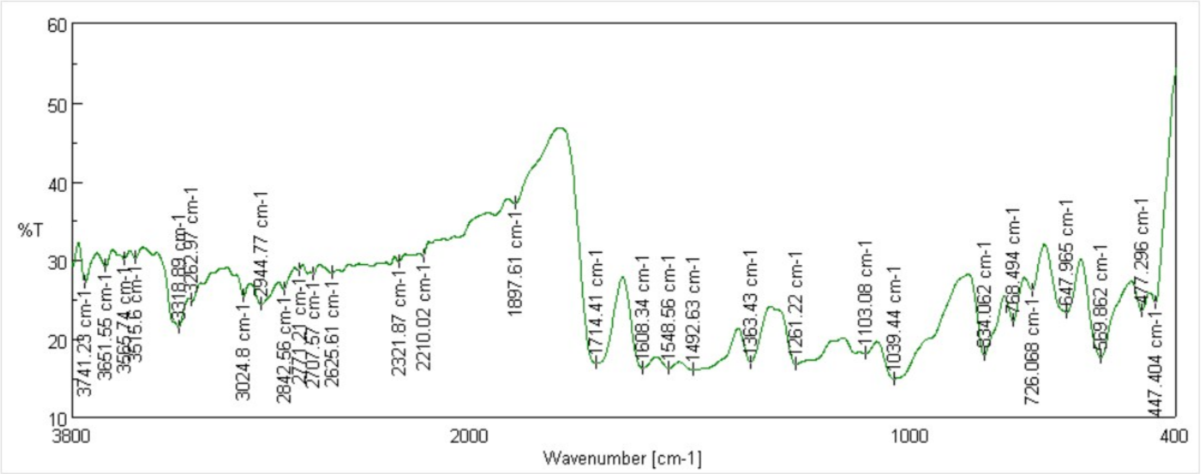

Fig. S4. IR spectrum of **[H_2-7MAC-mtsc]** (**H_2L^2**)

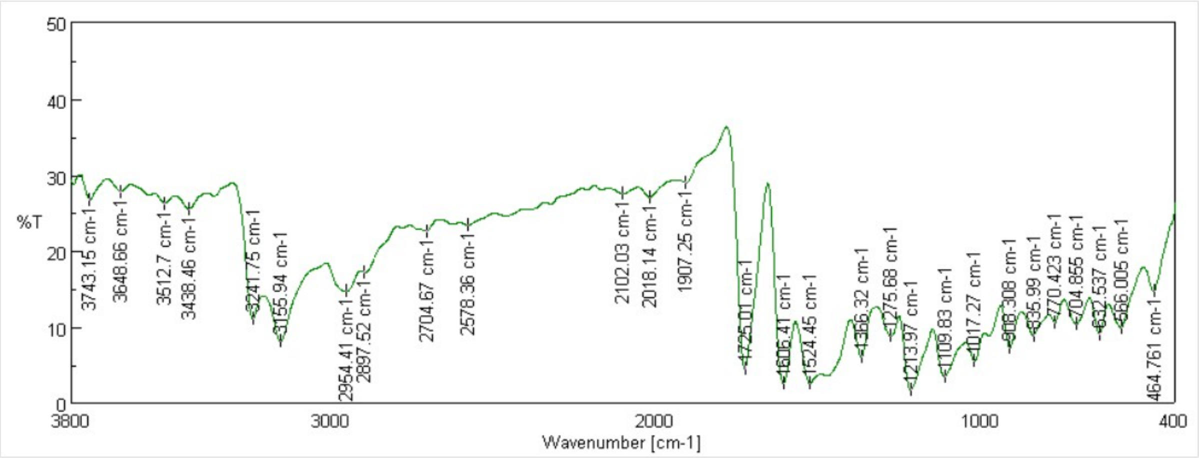

Fig. S5. IR spectrum of **[H_2-7MAC-etsc]** (**H_2L^3**)

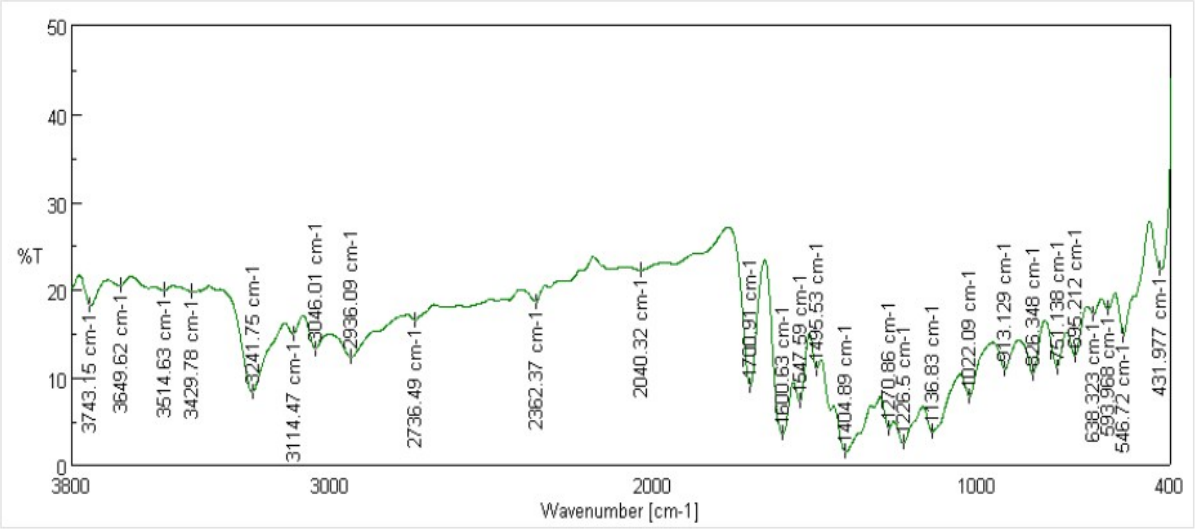

Fig. S6. IR spectrum of **[H_2-7MAC-ptsc]** (**H_2L^4**)

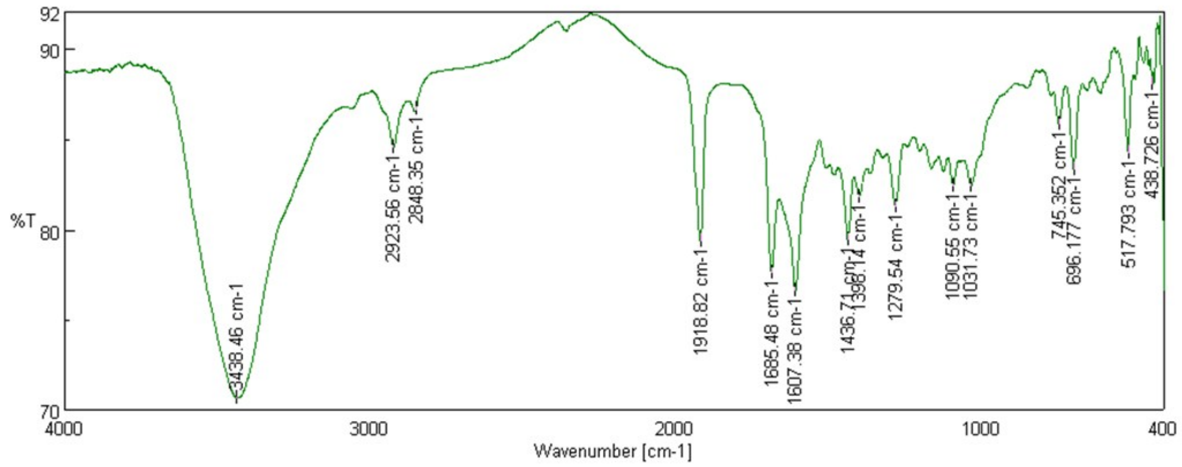

Fig. S7. IR spectrum of **[Ru(7MAC-tsc)CO(PPh_3)$_2$]** (**1**)

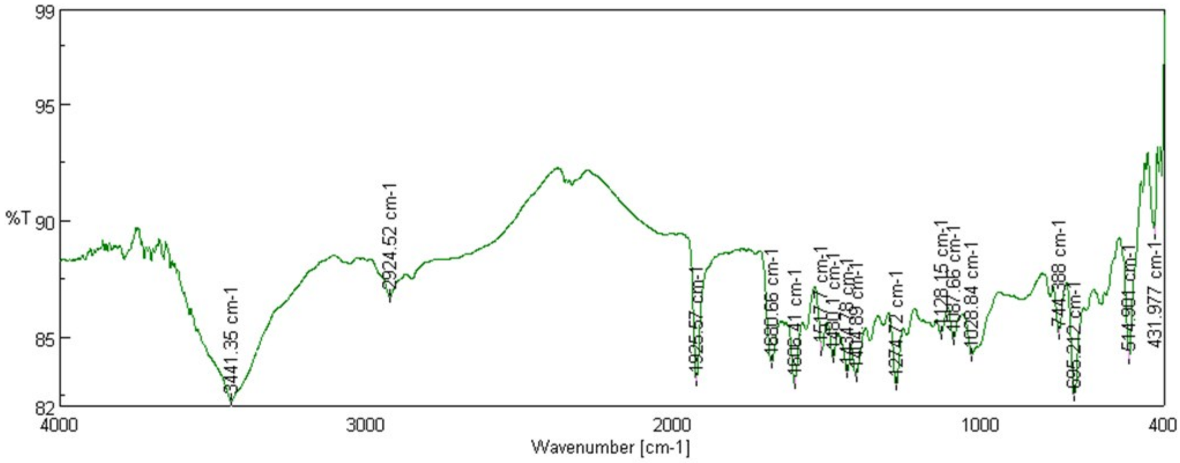

Fig. S8. IR spectrum of **[Ru(7MAC-mtsc)CO(PPh$_3$)$_2$]** (**2**)

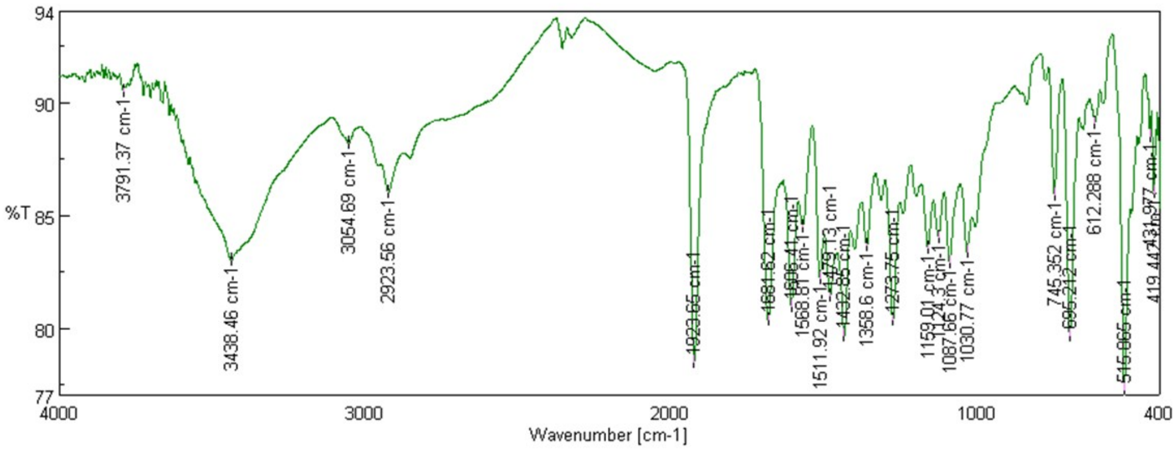

Fig. S9. IR spectrum of **[Ru(7MAC-etsc)CO(PPh$_3$)$_2$]** (**3**)

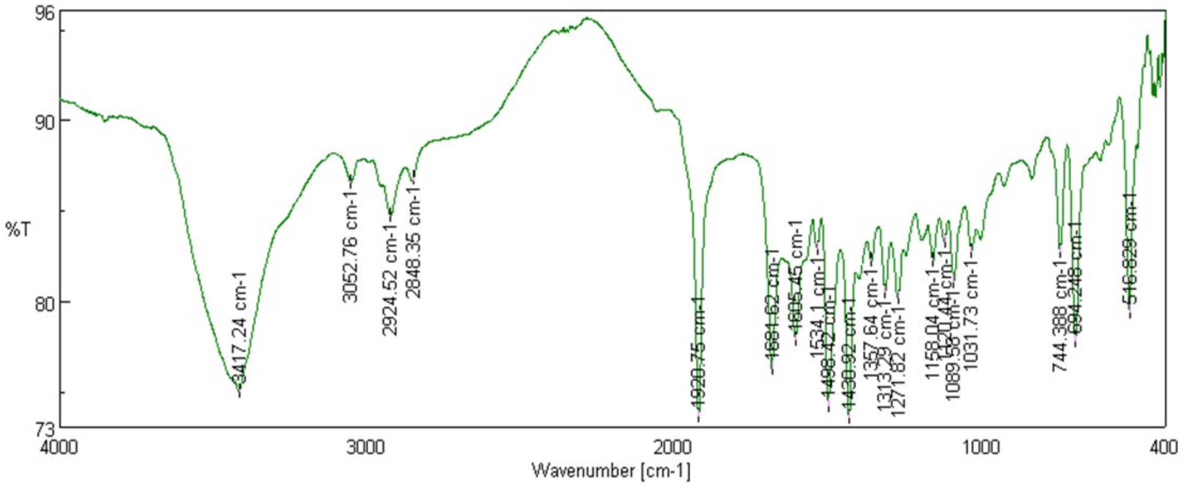

Fig. S10. IR spectrum of **[Ru(7MAC-ptsc)CO(PPh$_3$)$_2$]** (**4**)

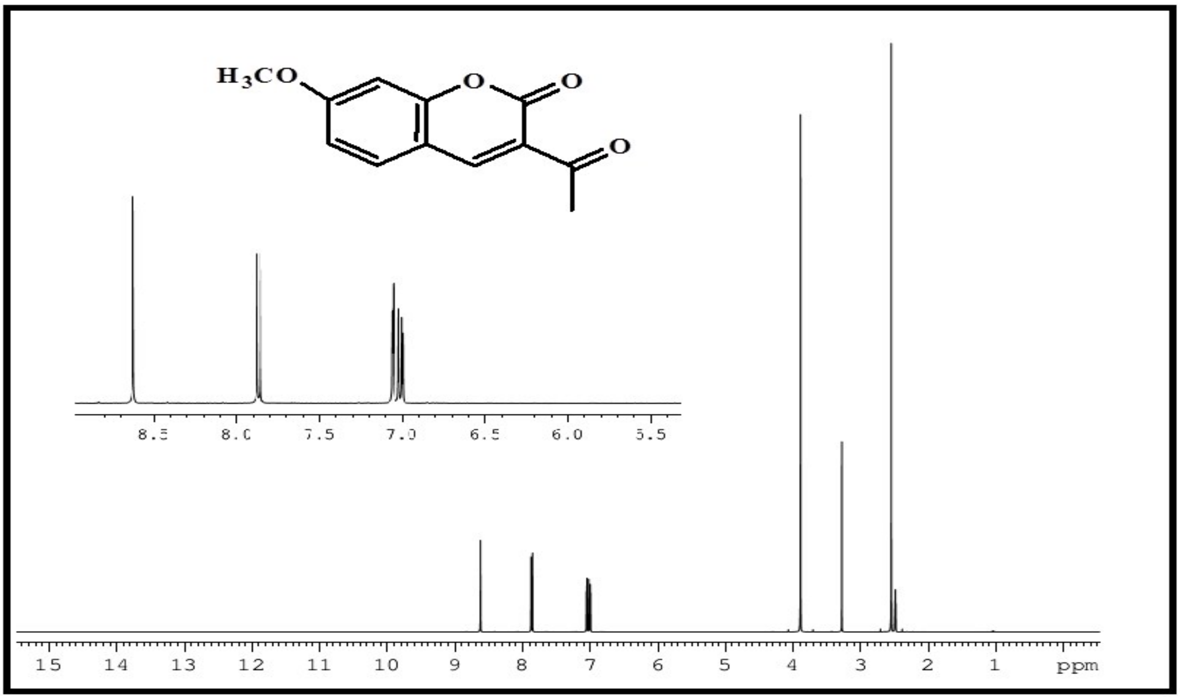

Fig. S11. ^{1}H NMR spectrum of 3-acetyl-7-methoxy coumarin

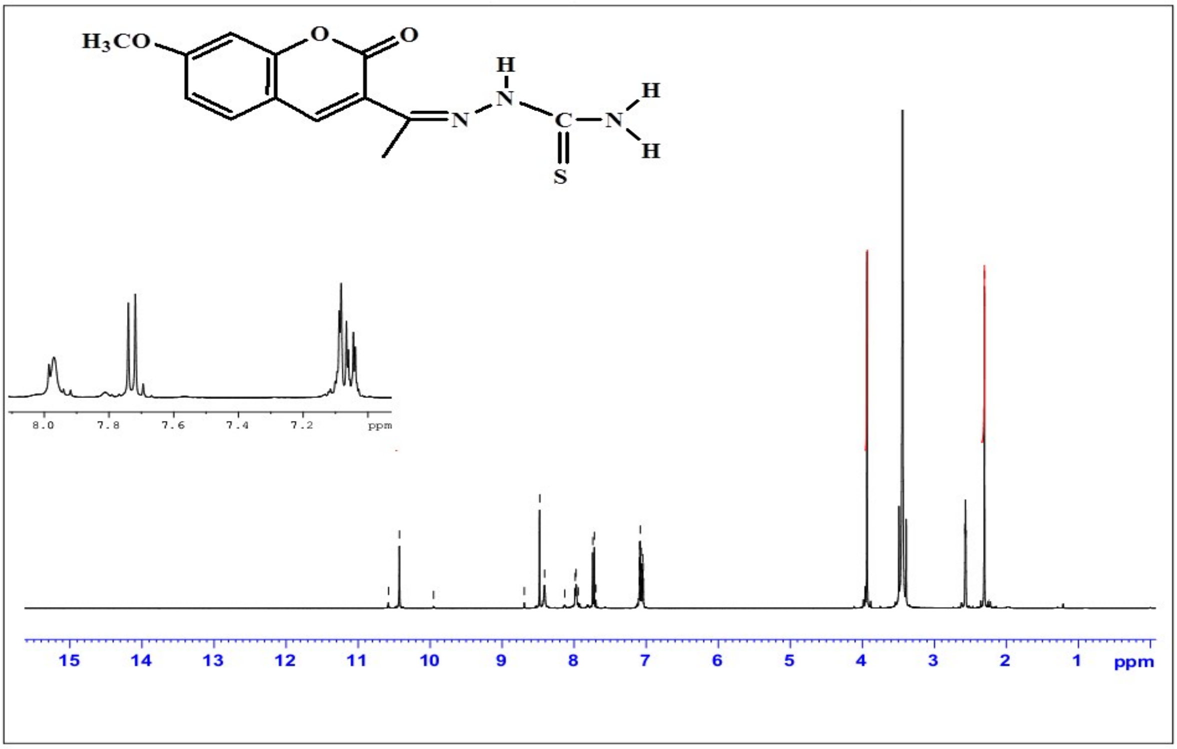

Fig. S12. ^{1}H NMR spectrum of **[H$_2$-7MAC-tsc]** (**H$_2$L^1**)

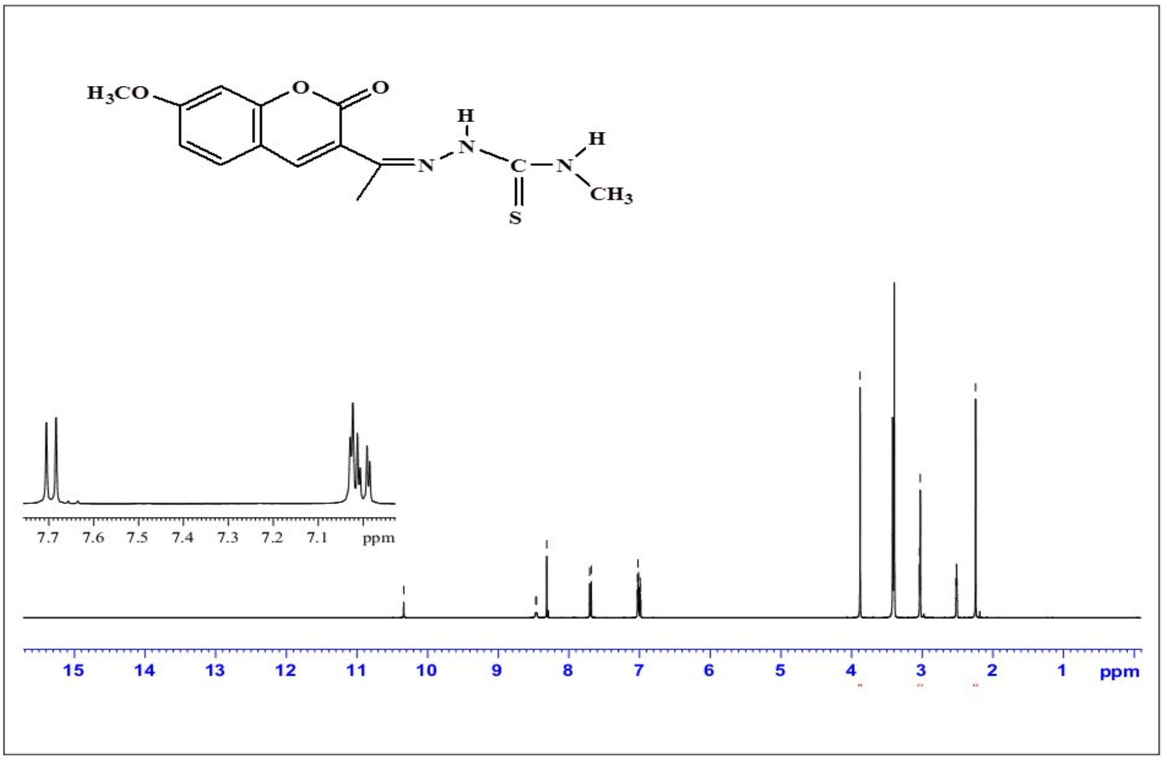

Fig. S13. ^{1}H NMR spectrum of **[H_2-7MAC-mtsc]** (**H_2L^2**)

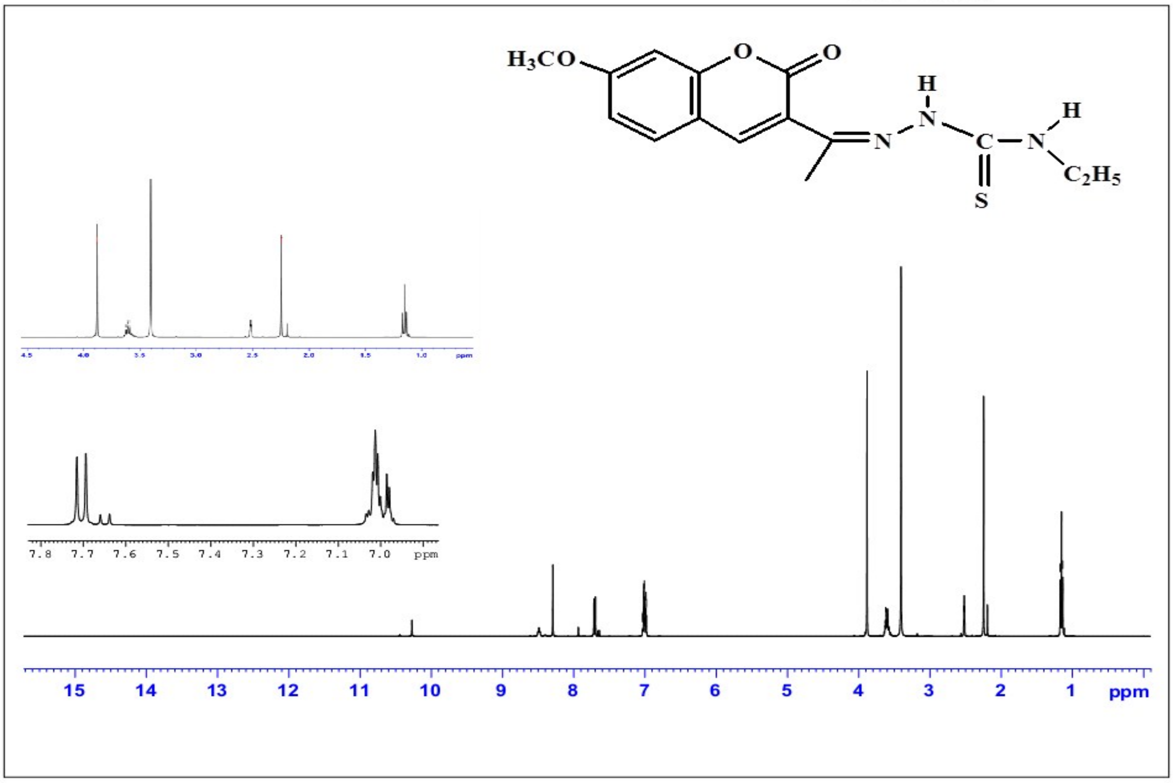

Fig. S14. ^{1}H NMR spectrum of **[H_2-7MAC-etsc]** (**H_2L^3**)

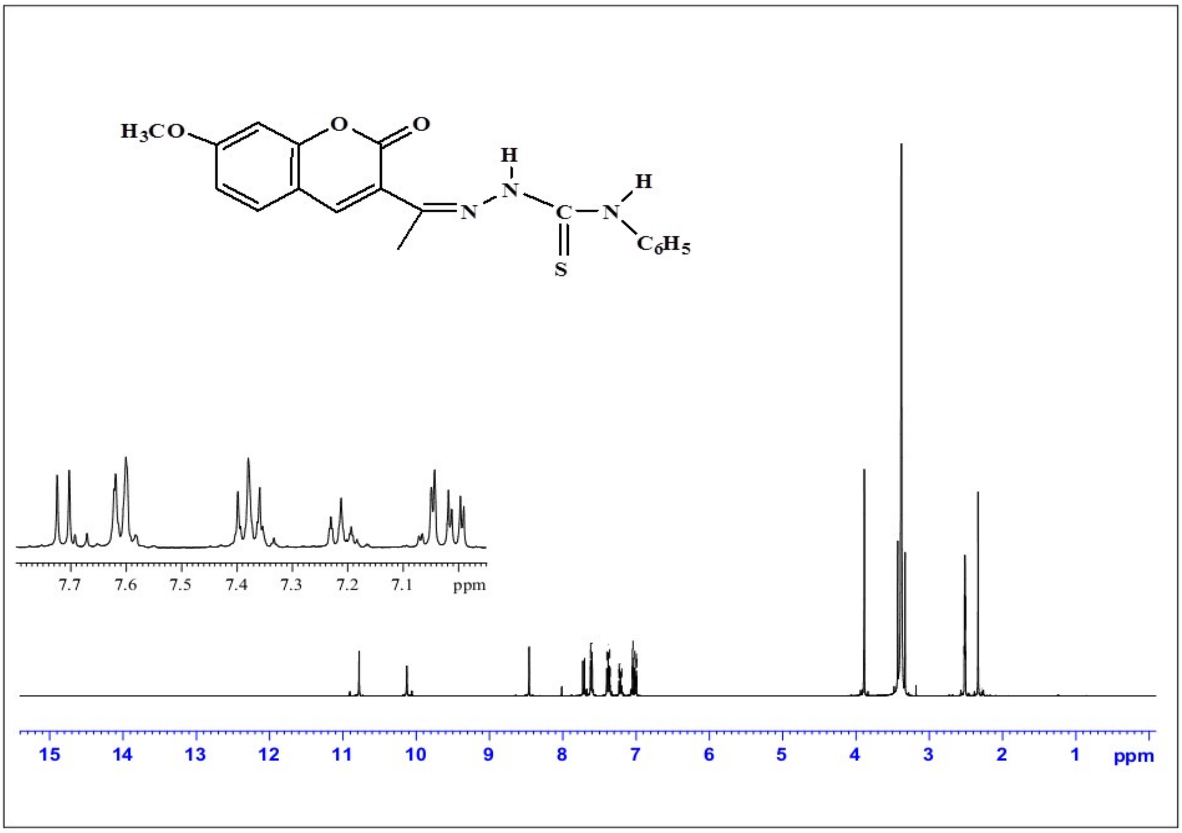

Fig. S15. ^{1}H NMR spectrum of **[H$_2$-7MAC-ptsc]** (**H$_2$L^4**)

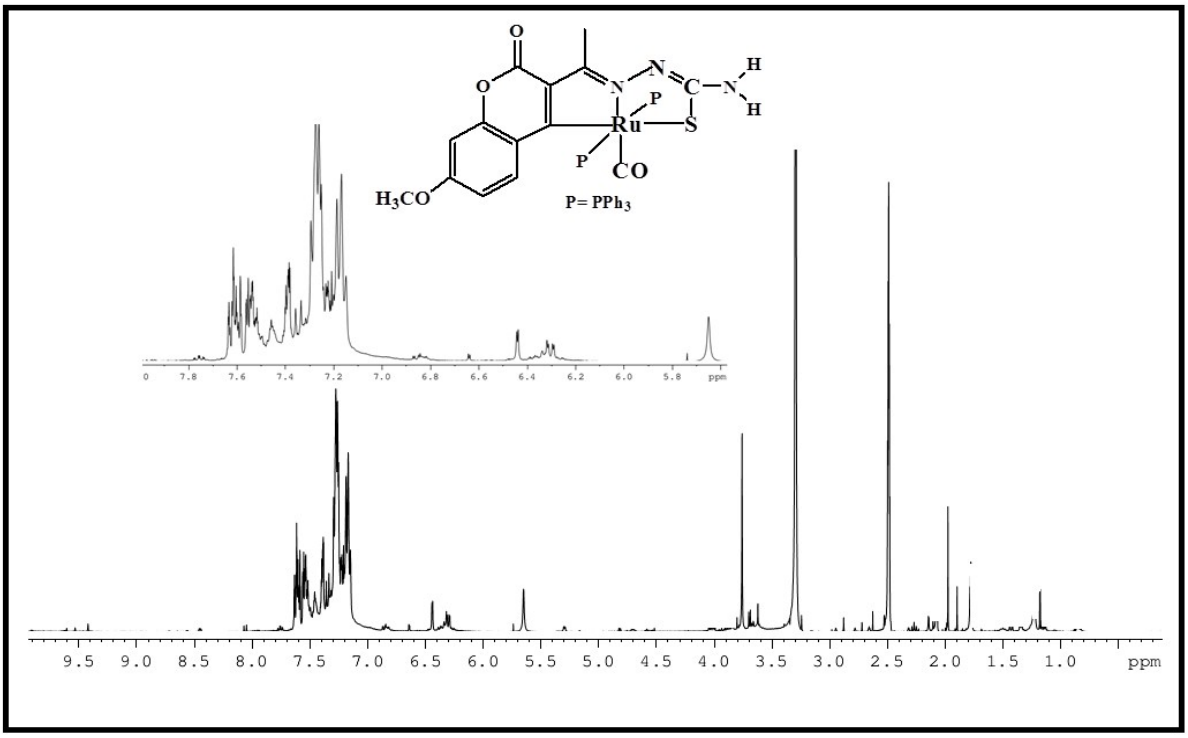

Fig. S16. ^{1}H NMR spectrum of **[Ru(7MAC-tsc)CO(PPh$_3$)$_2$]** (**1**)

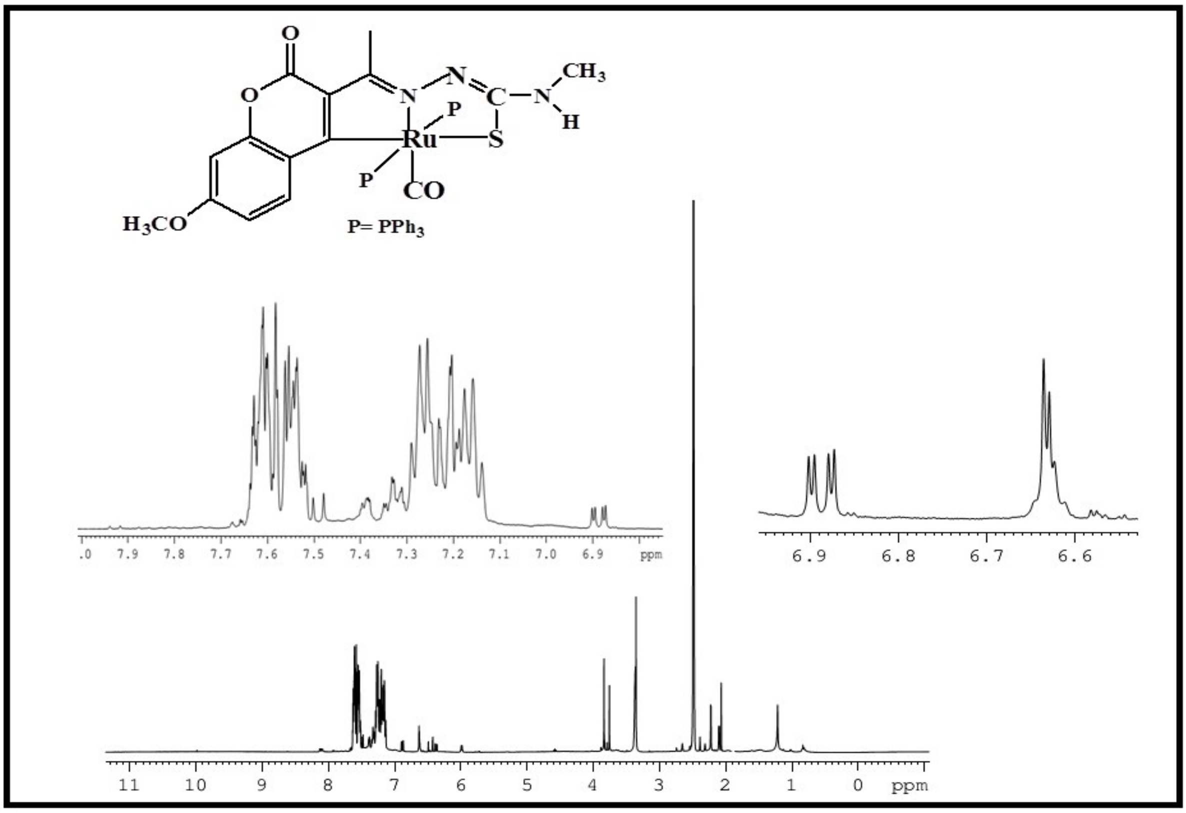

Fig. S17. ^{1}H NMR spectrum of **[Ru(7MAC-mtsc)CO(PPh$_3$)$_2$]** (**2**)

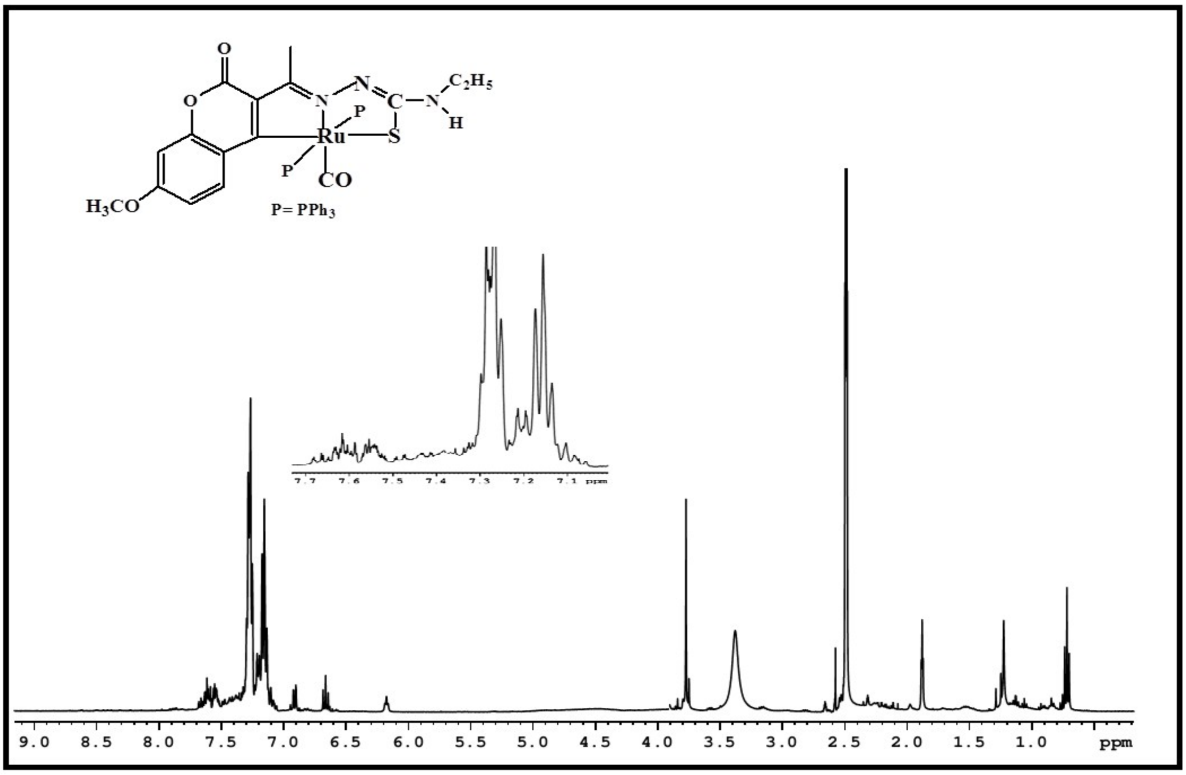

Fig. S18. ^{1}H NMR spectrum of **[Ru(7MAC-etsc)CO(PPh$_3$)$_2$]** (**3**)

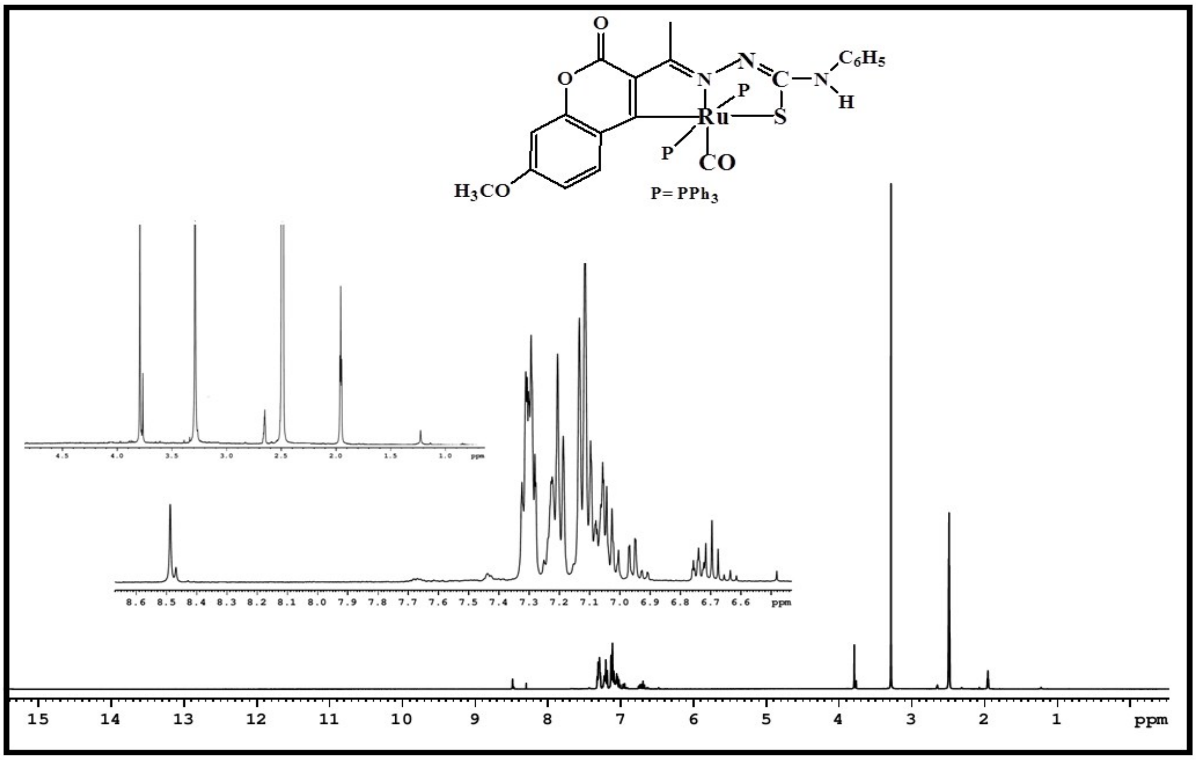

Fig. S19. ^{1}H NMR spectrum of **[Ru(7MAC-ptsc)CO(PPh$_3$)$_2$]** (**4**)

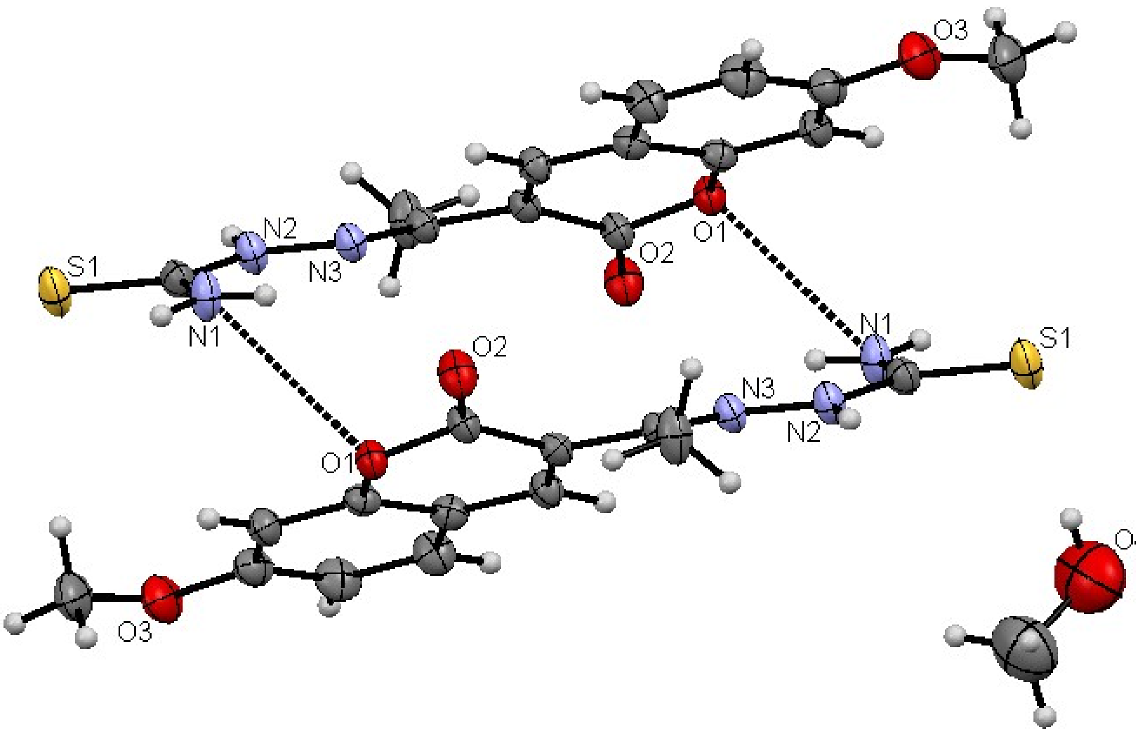

Fig. S20. ORTEP diagram of **[H$_2$-7MAC-tsc]** (**H$_2$L^1**) with hydrogen bonding

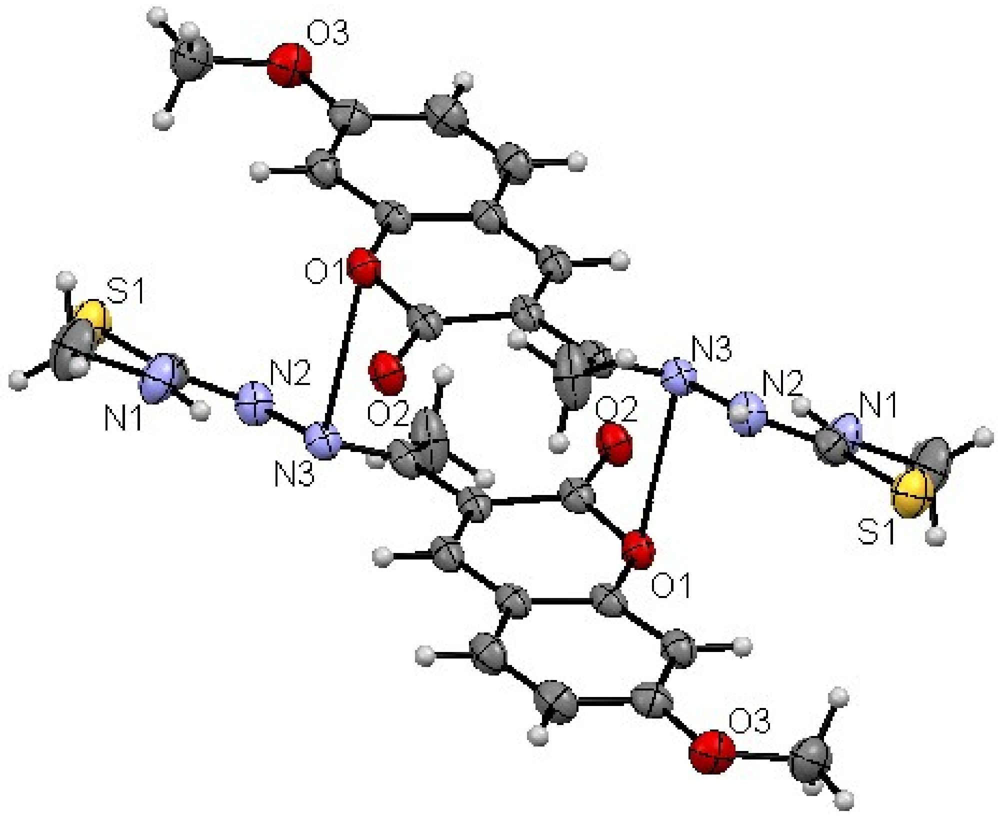

Fig. S21. ORTEP diagram of **[H_2-7MAC-mtsc]** (**H_2L^2**) with hydrogen bonding

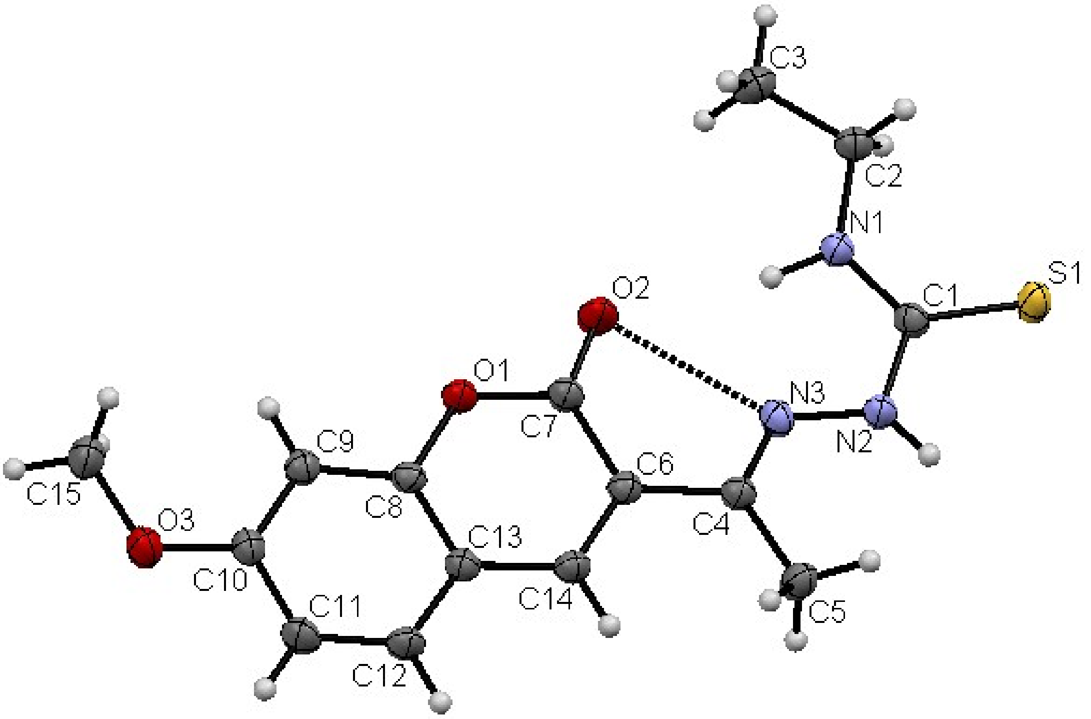

Fig. S22. ORTEP diagram of **[H_2-7MAC-etsc]** (**H_2L^3**) with hydrogen bonding

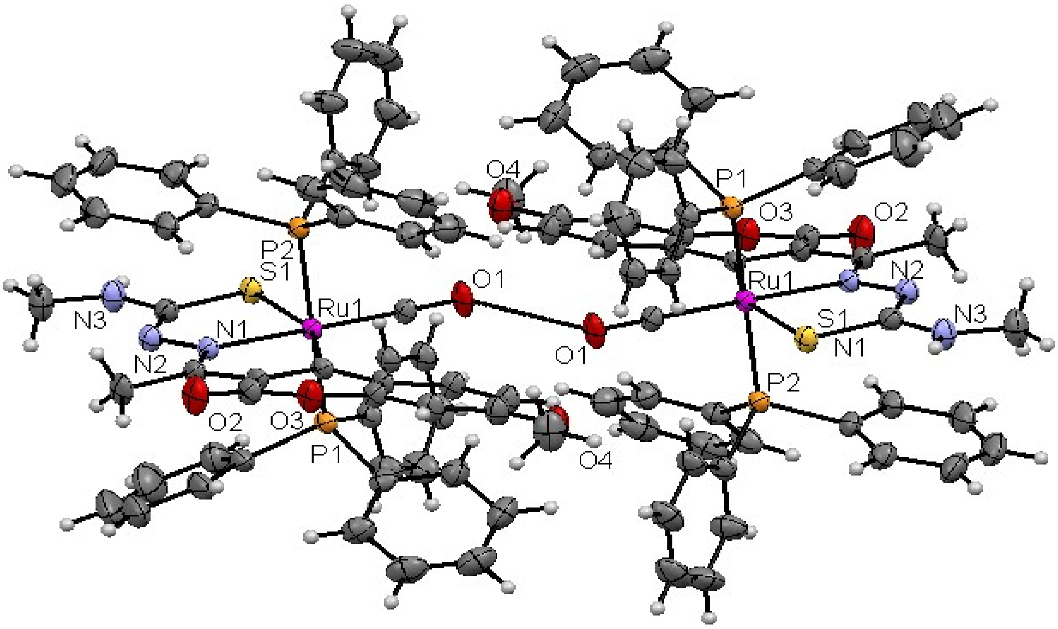

Fig. S23. ORTEP diagram of **[Ru(7MAC-mtsc)CO(PPh$_3$)$_2$]** (**2**) with hydrogen bonding

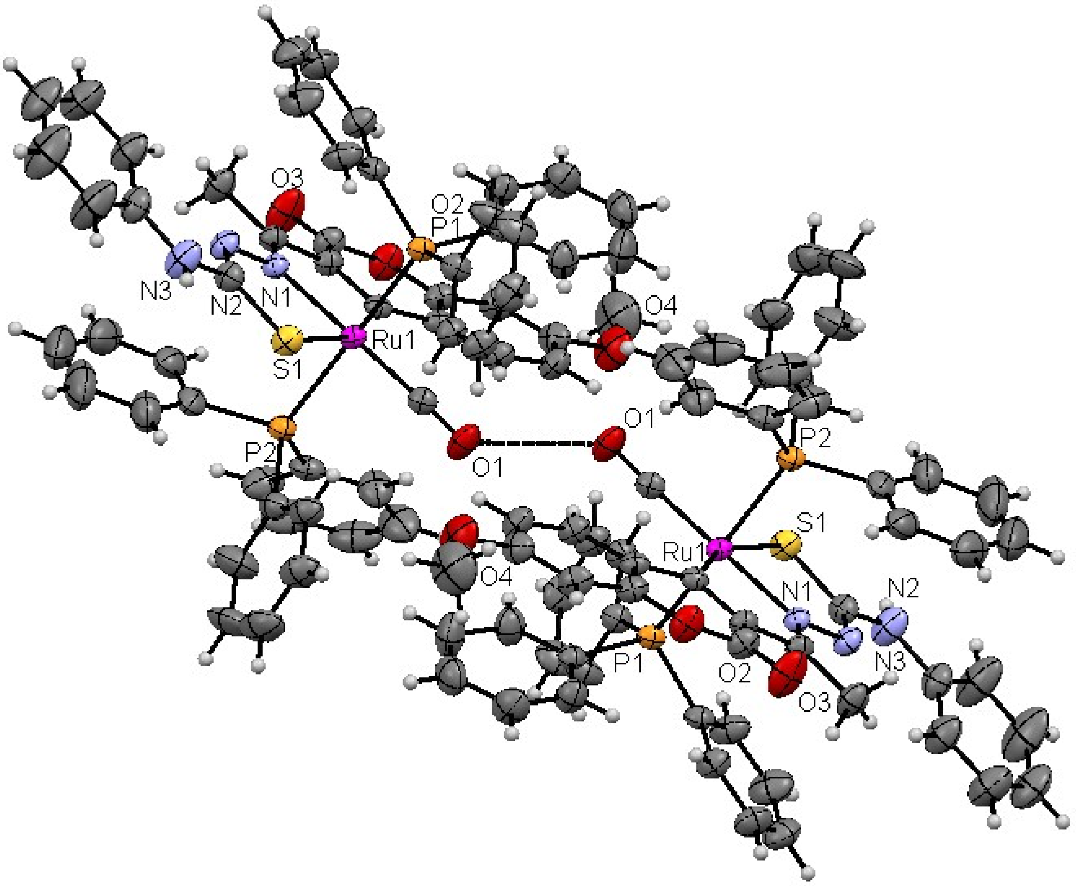

Fig. S24. ORTEP diagram of **[Ru(7MAC-mtsc)CO(PPh$_3$)$_2$]** (**2**) with hydrogen bonding

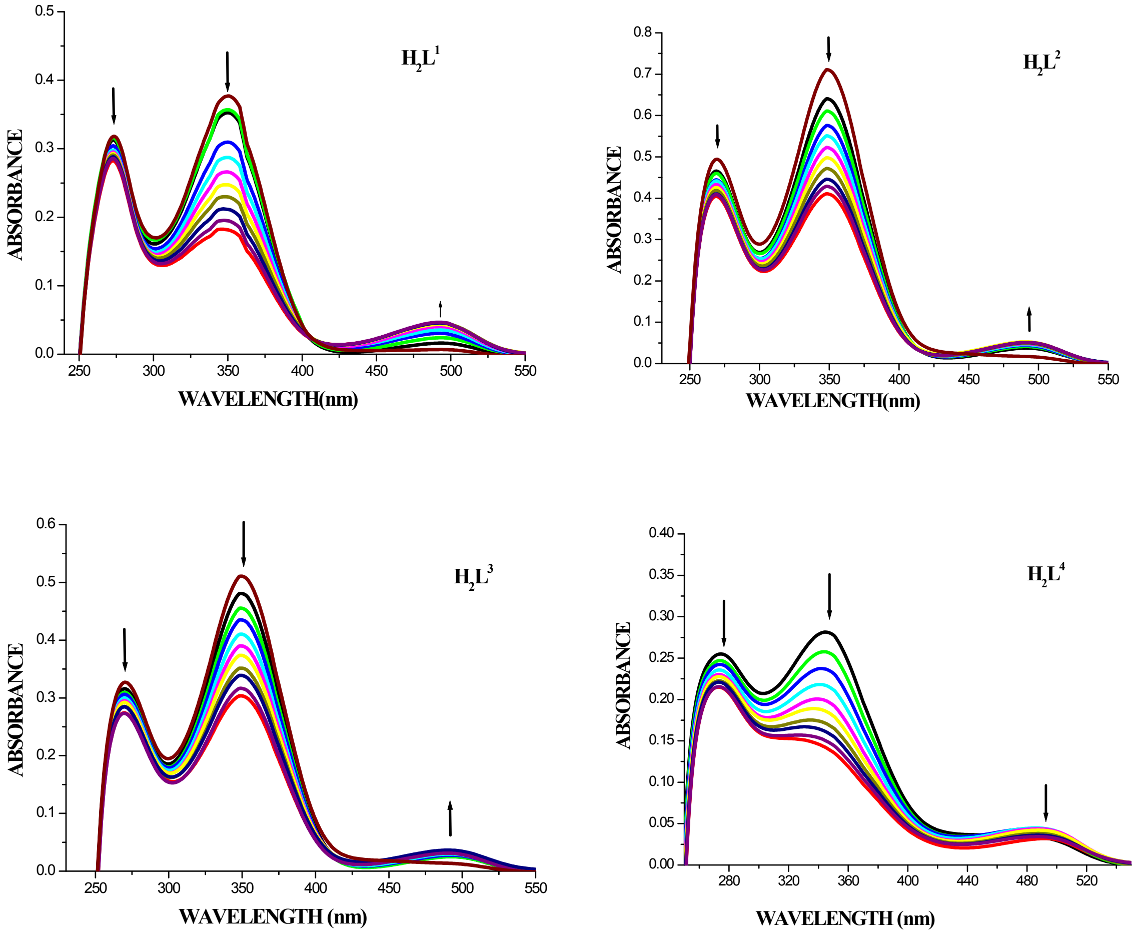

Fig. S25. Absorption titration spectra of ligands (**H_2L^{1-4}**) with increasing concentrations (2.5-25 μM) of CT-DNA (tris HCl buffer, pH 7.2)

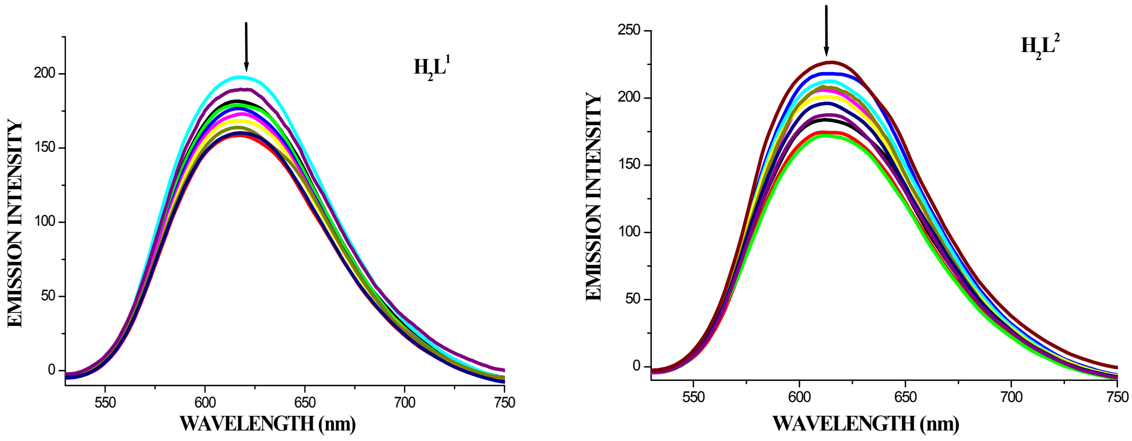

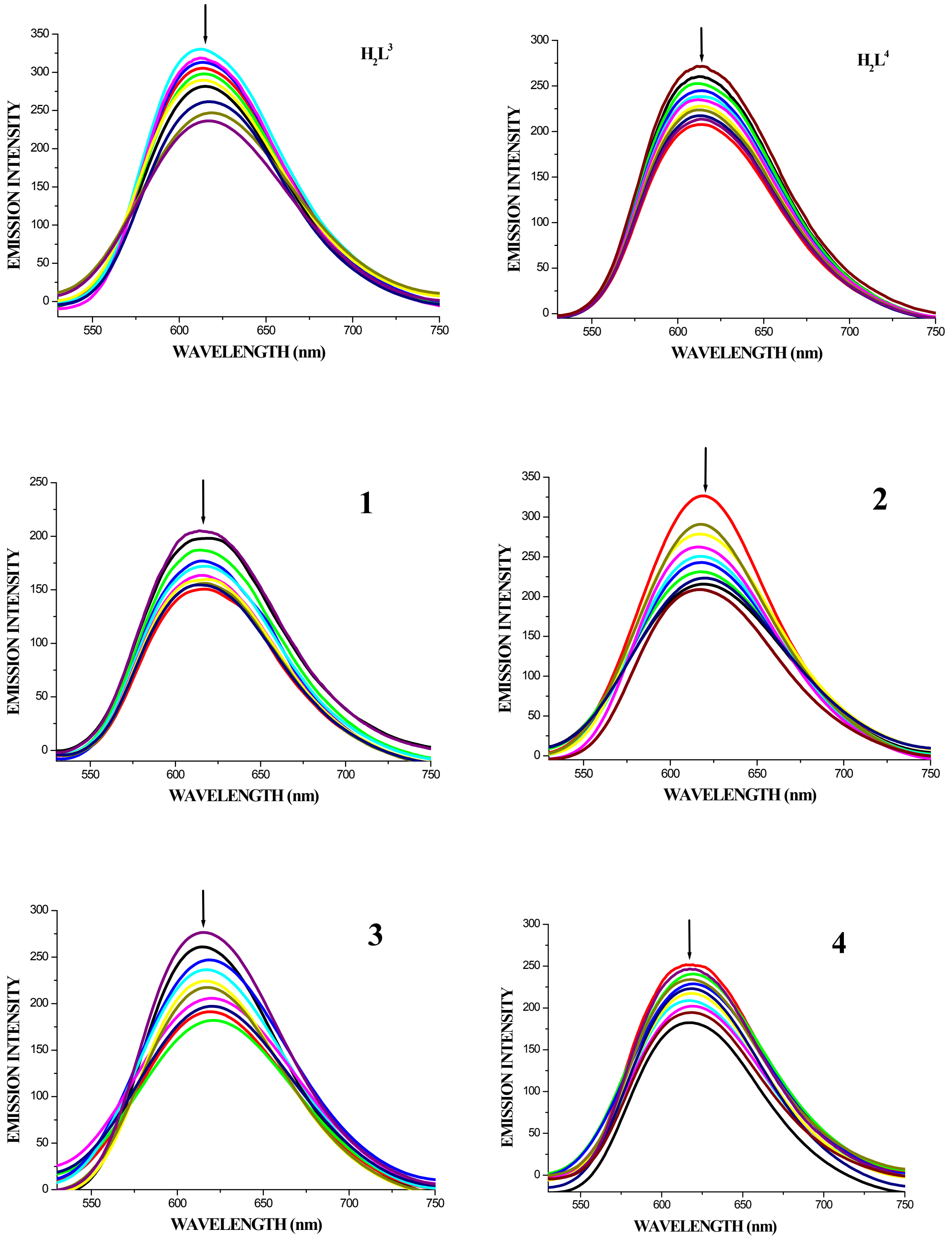

Fig. S26. The emission spectra of the DNA–EB system (λ_{exc}= 515 nm, λ_{em}= 530–750 nm), in the presence of ligands $\mathbf{H_2L^{1\text{-}4}}$ and complexs **1-4**. [DNA] = 10 μM, [Ligand] = 10–100 μM, [complex] = 10–100 μM, [EB] = 10 μM. The arrow shows the emission intensity changes upon increasing complex concentration

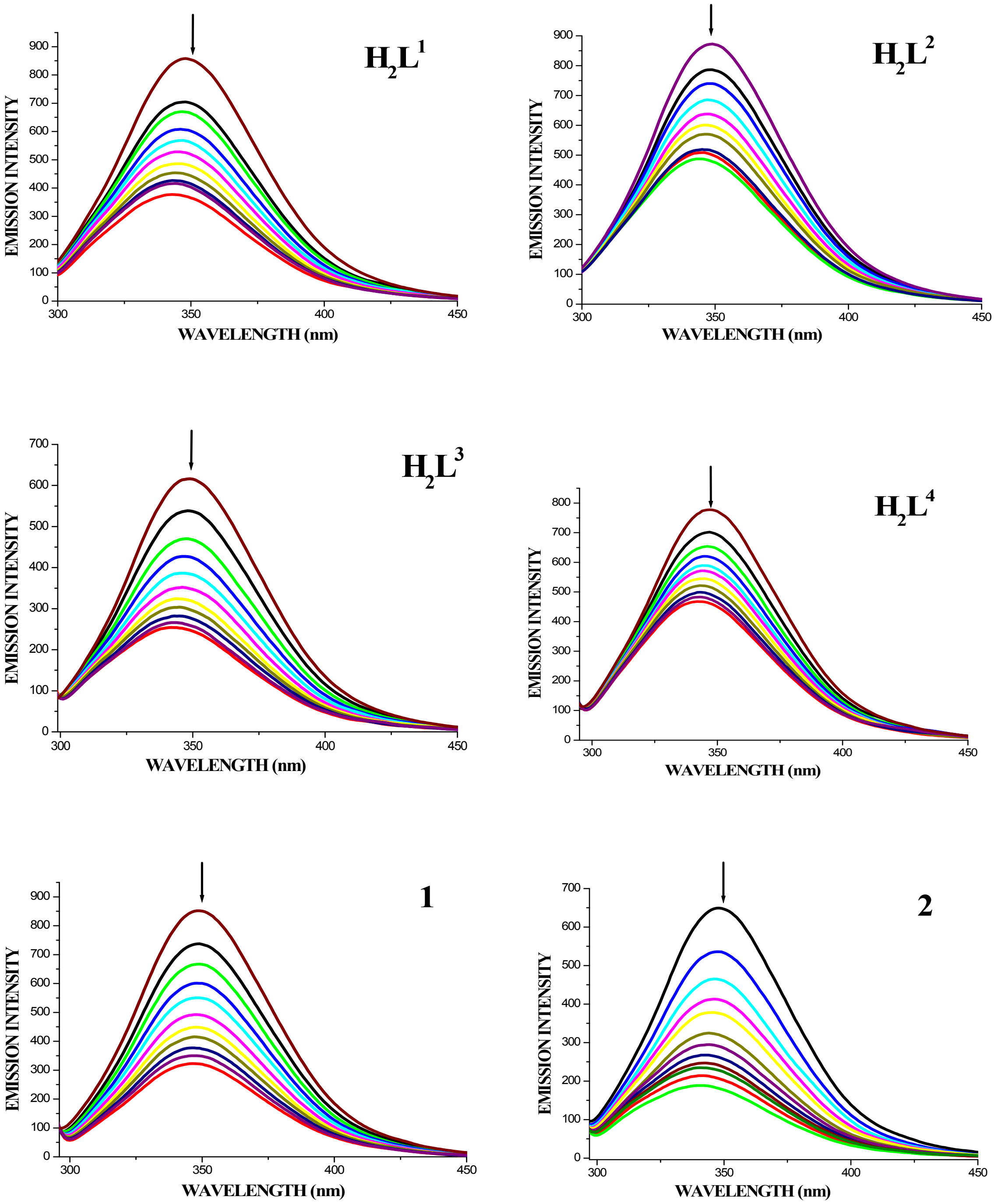

H2L1
H2L2
H2L3
H2L4
1
2
EMISSION INTENSITY
WAVELENGTH (nm)

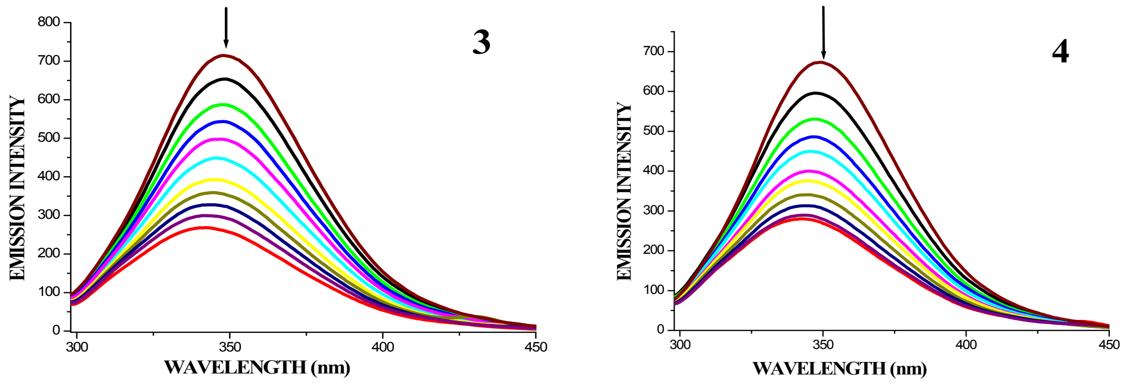

Fig. S27. The emission spectra of BSA (10 μM; λexc= 280 nm; λemi= 346 nm) in the presence of increasing amounts of ligands $\mathbf{H_2L^{1\text{-}4}}$ and complexes **1-4** (10–100 μM). The arrow shows the emission intensity changes upon increasing complex concentration

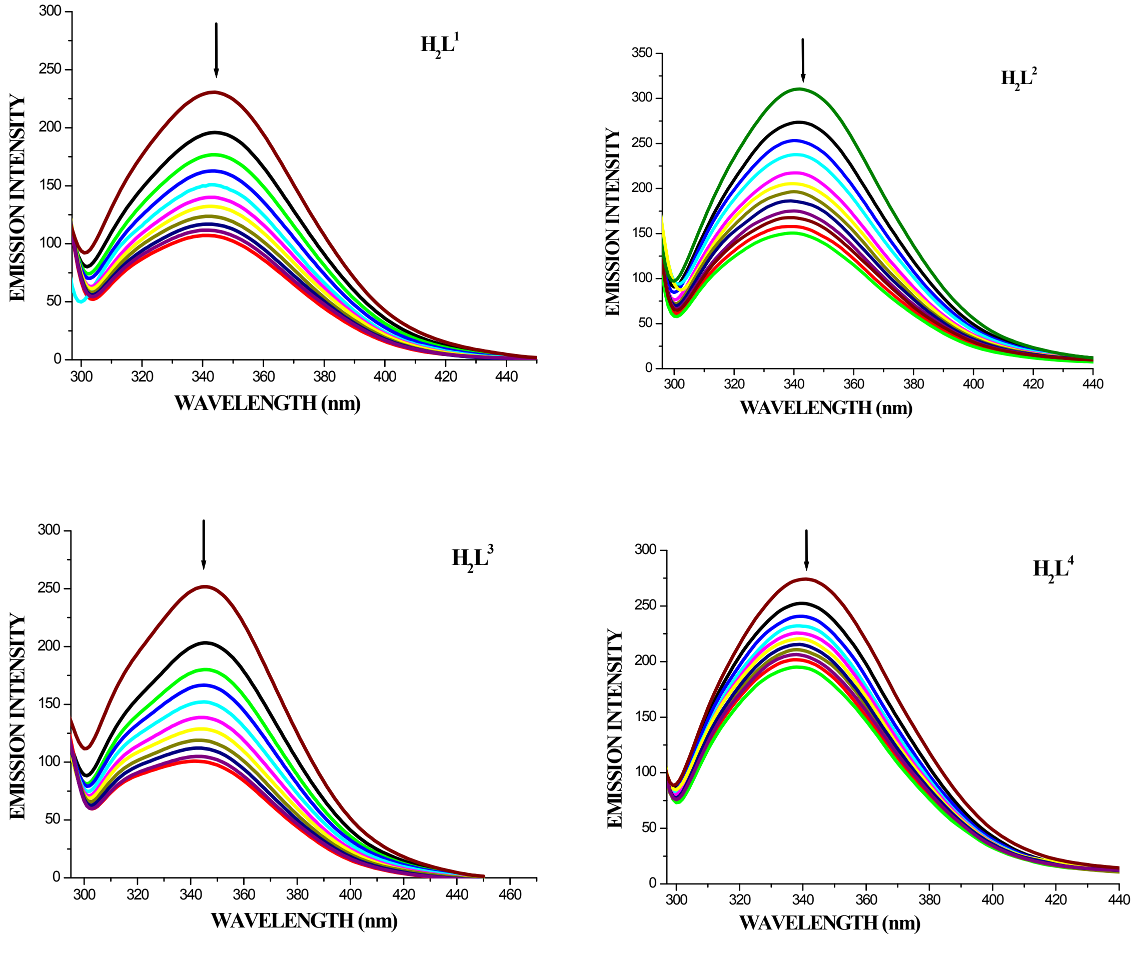

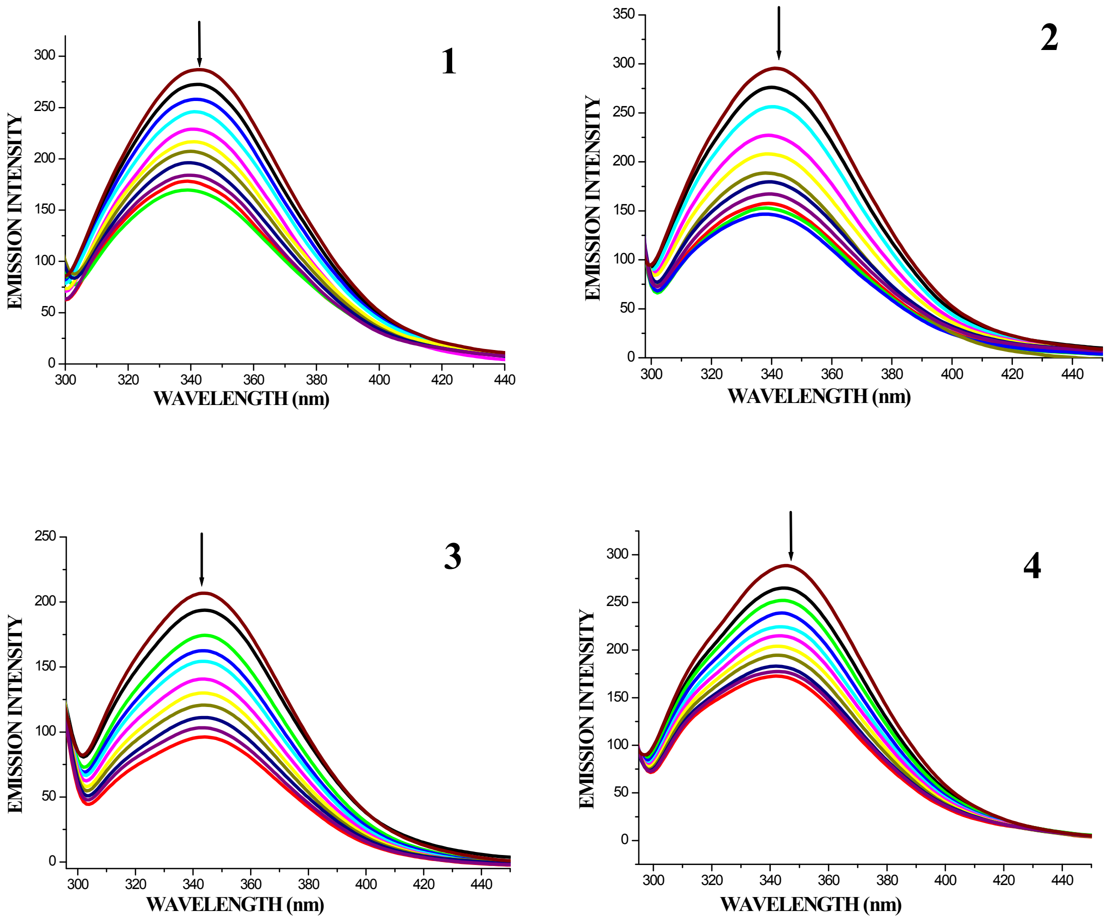

Fig. S28. The emission spectra of HSA (10 μM; λexc= 290 nm; λemi= 345 nm) in the presence of increasing amounts of ligands **H_2L^{1-4}** and complexes **1-4** (10–100 μM). The arrow shows the emission intensity changes upon increasing complex concentration

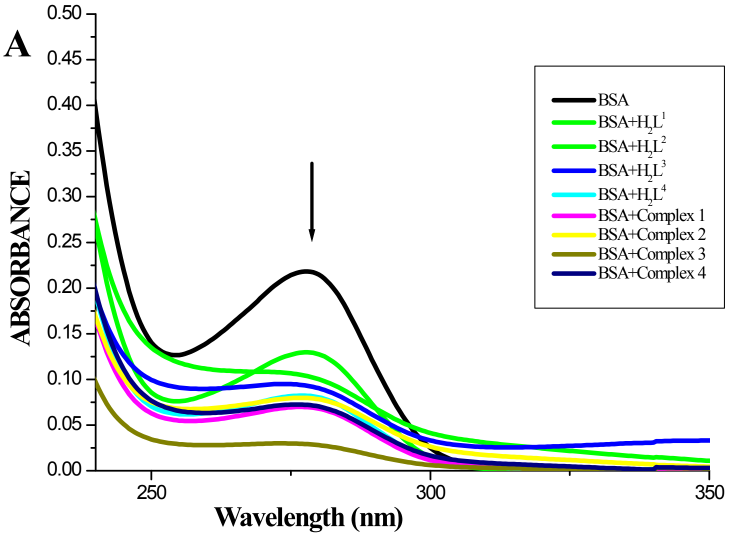

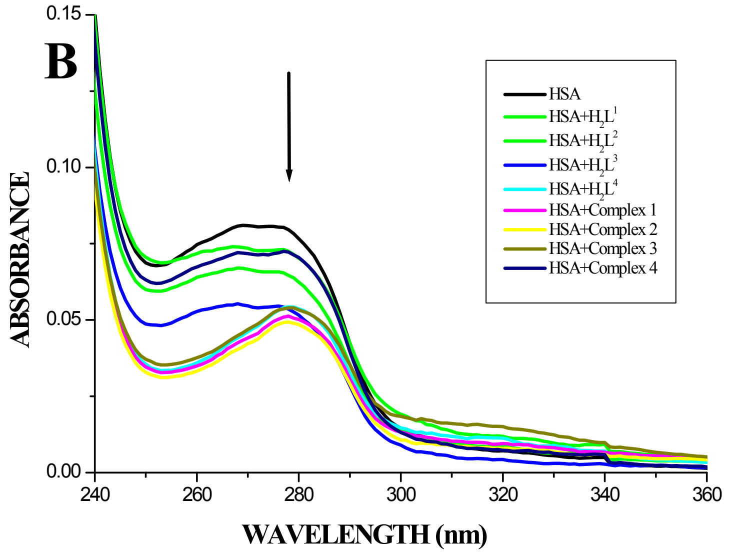

Fig. S29. A) Absorption spectra of absence and presence of ligands $\mathbf{H_2L^{1-4}}$ and complexes (**1-4**) with BSA (1×10^{-5}M) **B)** Absorption spectra of absence and presence of ligands $\mathbf{H_2L^{1-4}}$ and complexes (**1-4**) with HSA (1×10^{-5}M)

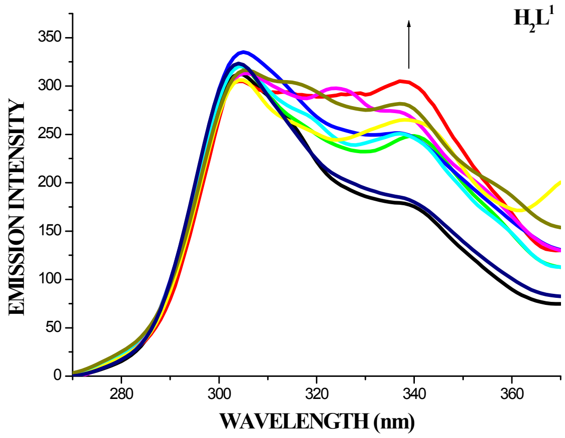

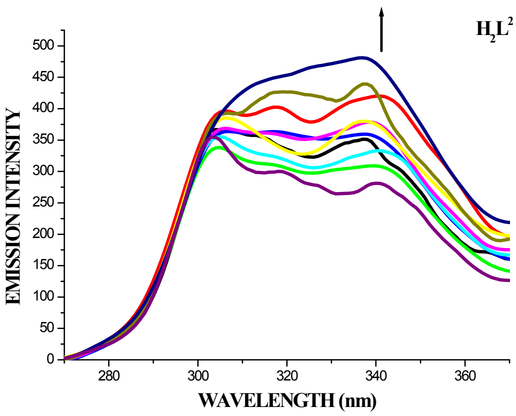

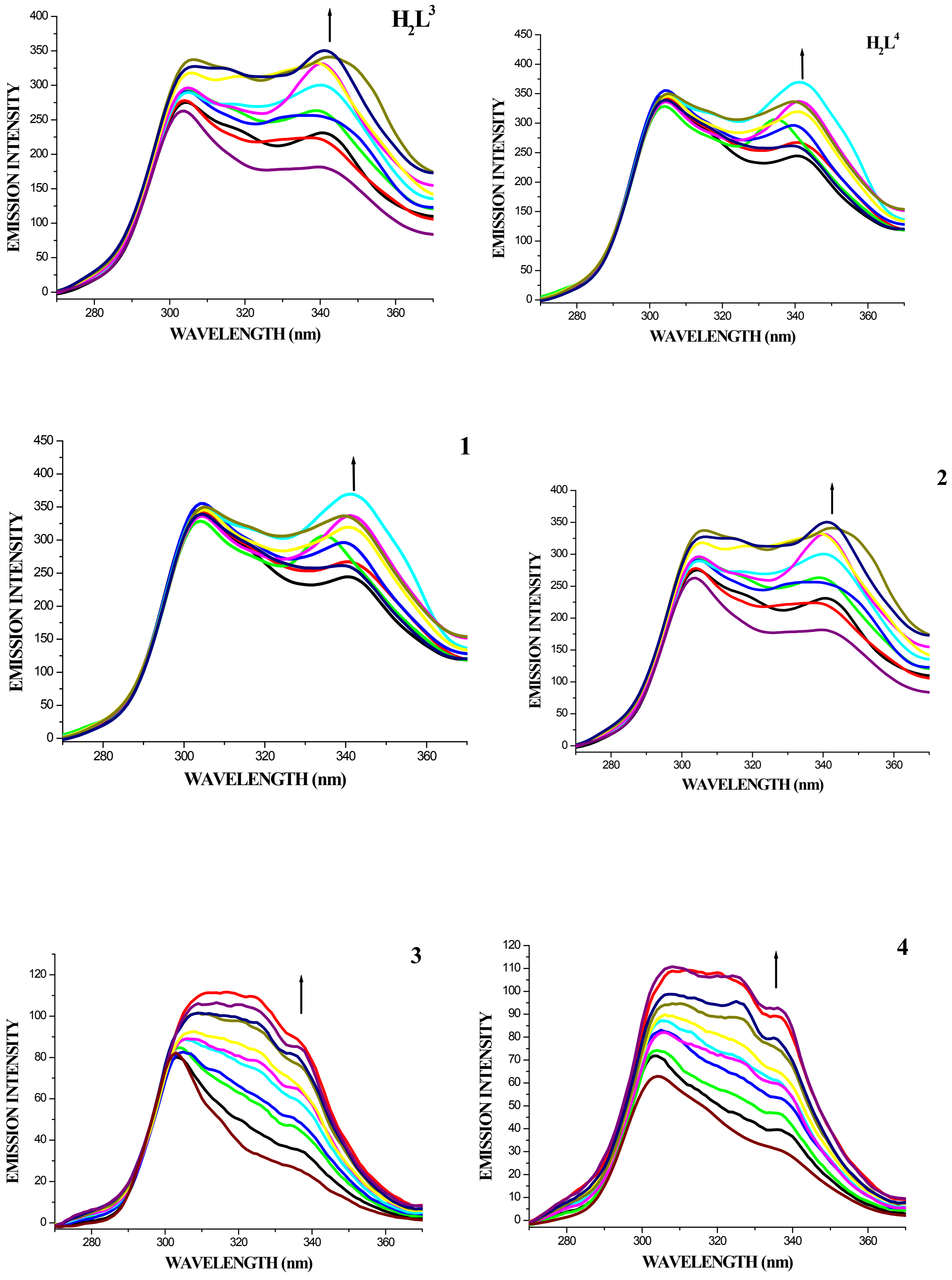

Fig. S30. Synchronous spectra of BSA (10 μM) in the presence of increasing amounts of ligands **H_2L^{1-4}** and complexes **1-4** (10–100 μM) for a wavelength difference of Δλ= 15 nm. The arrow shows the emission intensity changes upon increasing concentration of compounds

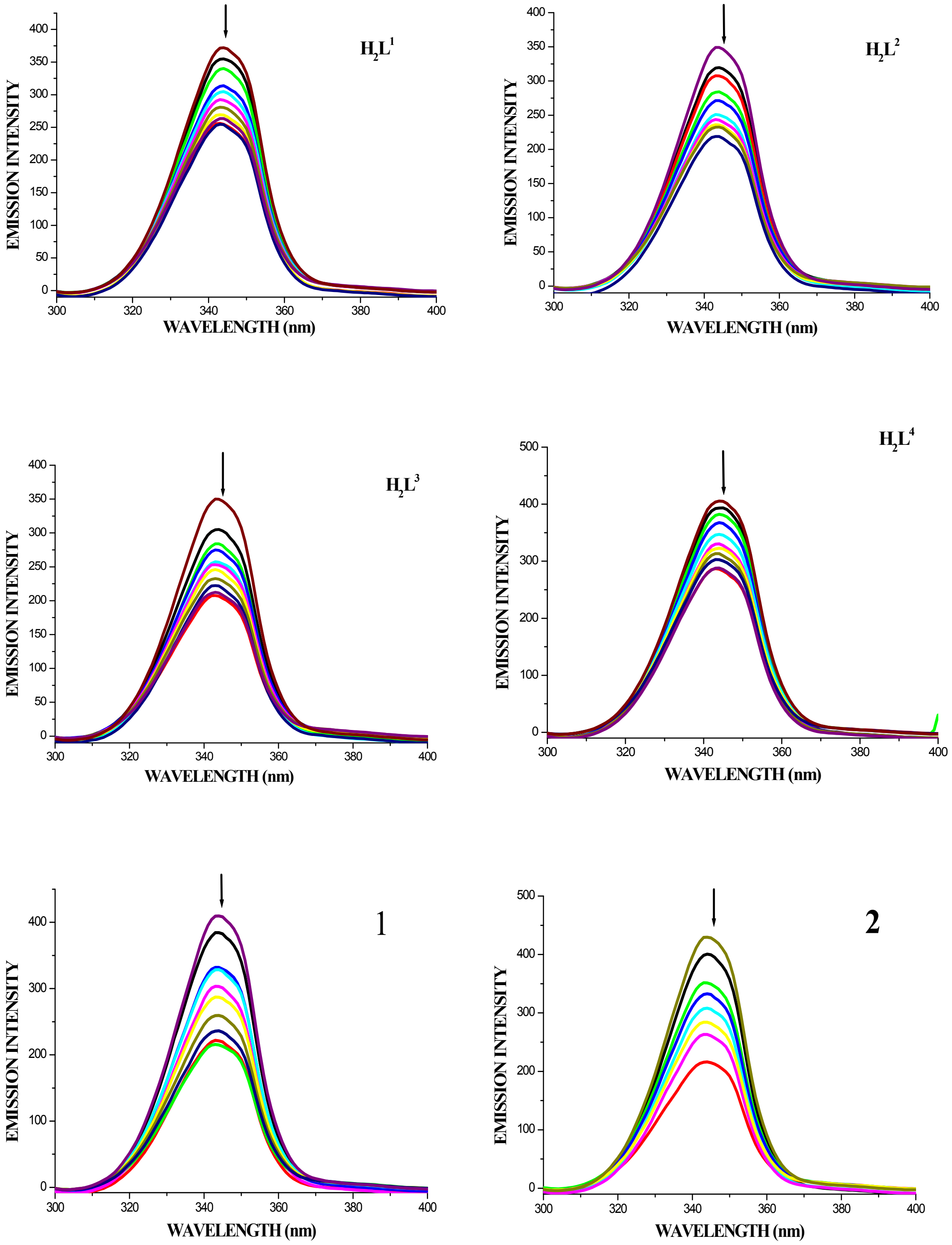
H2L1
H2L2
H2L3
H2L4
1
2
EMISSION INTENSITY
WAVELENGTH (nm)

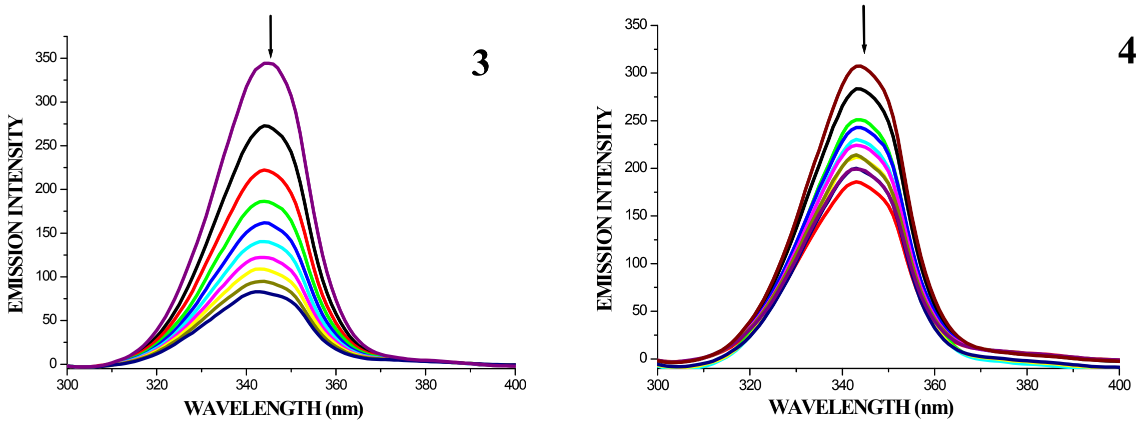

Fig. S31. Synchronous spectra of BSA (10 µM) in the presence of increasing amounts of ligands $\mathbf{H_2L^{1-4}}$ and complexes **1-4** (10–100 µM) for a wavelength difference of Δλ= 60 nm. The arrow shows the emission intensity changes upon increasing concentration of compounds

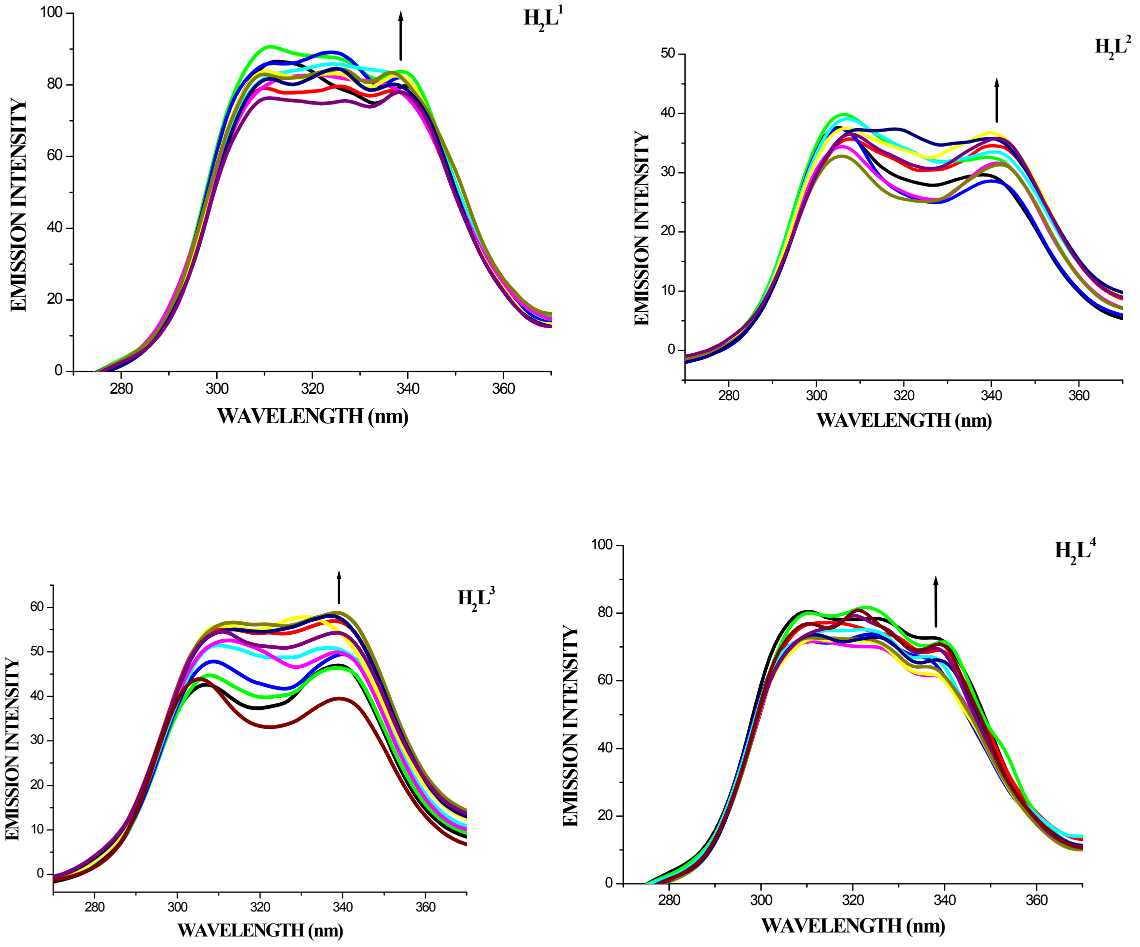

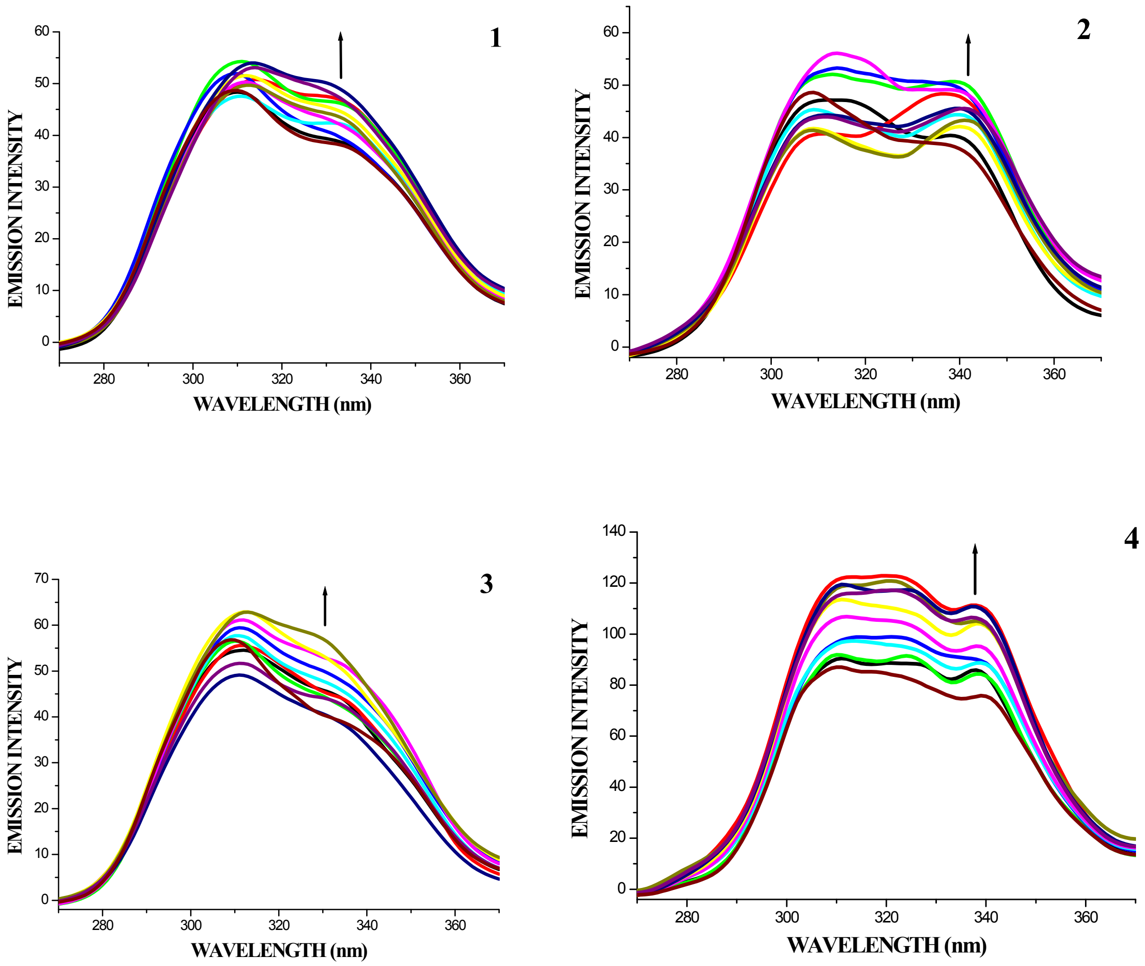

Fig. S32. Synchronous spectra of HSA (10 μM) in the presence of increasing amounts of ligands $\mathbf{H_2L^{1\text{-}4}}$ and complexes **1-4** (10–100 μM) for a wavelength difference of Δλ= 15 nm. The arrow shows the emission intensity changes upon increasing concentration of compounds

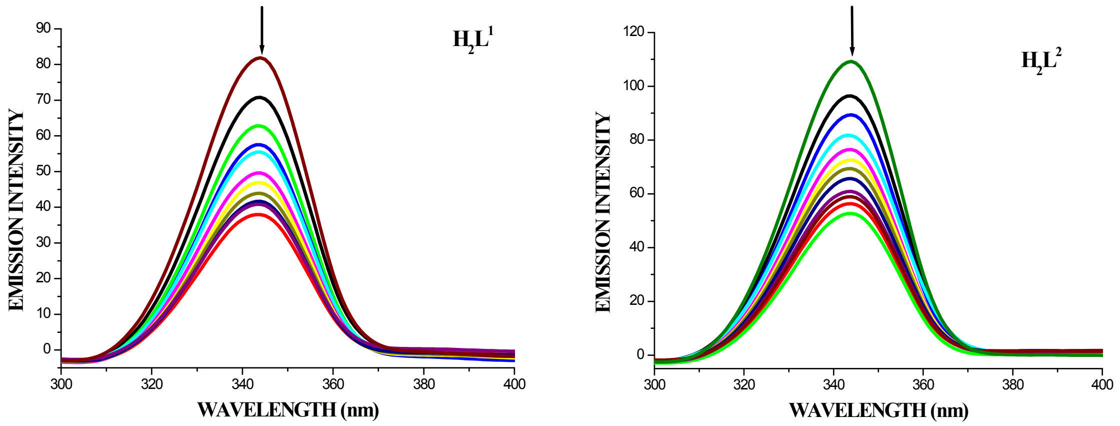

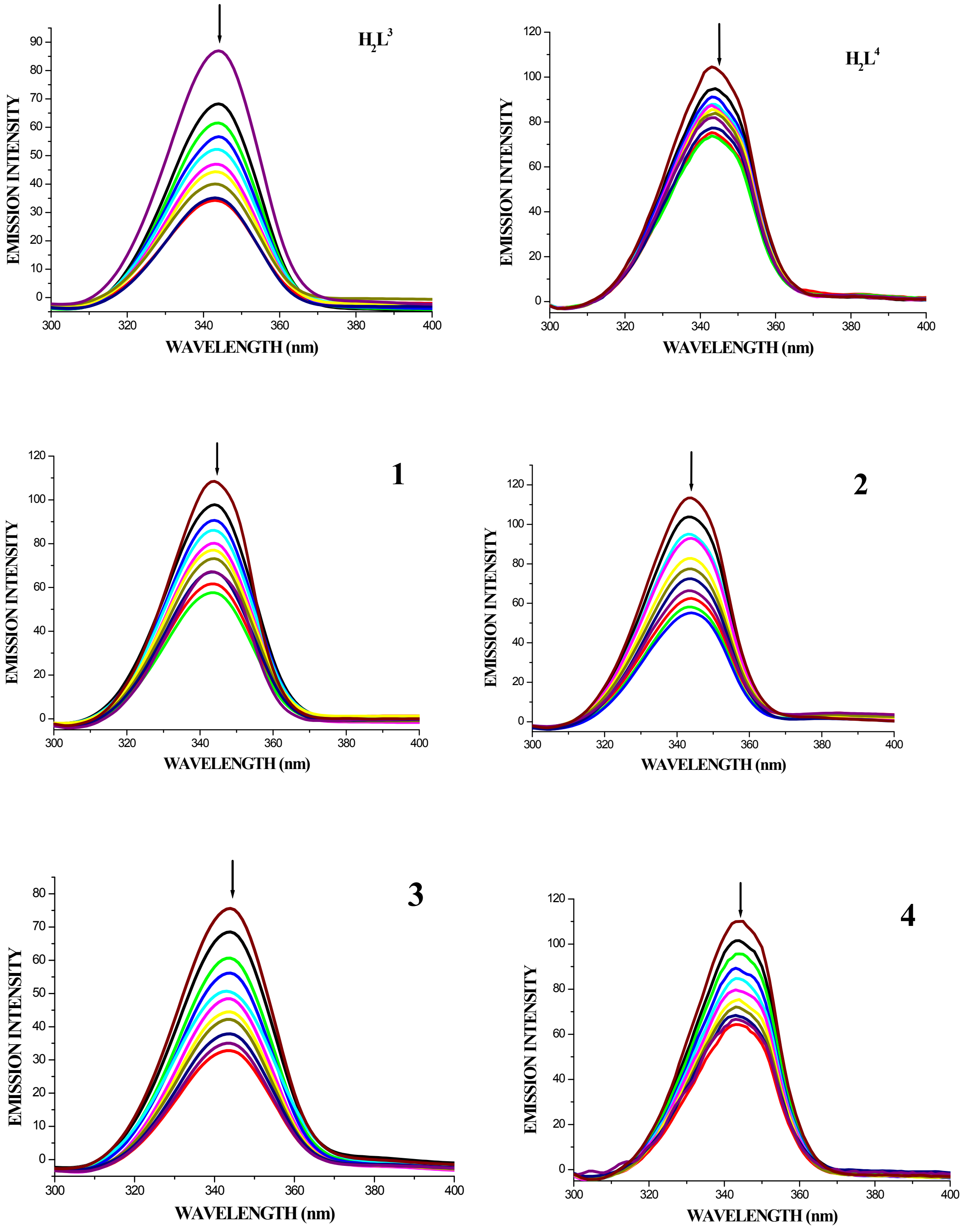

Fig. S33. Synchronous spectra of HSA (10 μM) in the presence of increasing amounts of ligands $\mathbf{H_2L^{1\text{-}4}}$ and complexes **1-4** (10–100 μM) for a wavelength difference of Δλ= 60 nm. The arrow shows the emission intensity changes upon increasing concentration of compounds

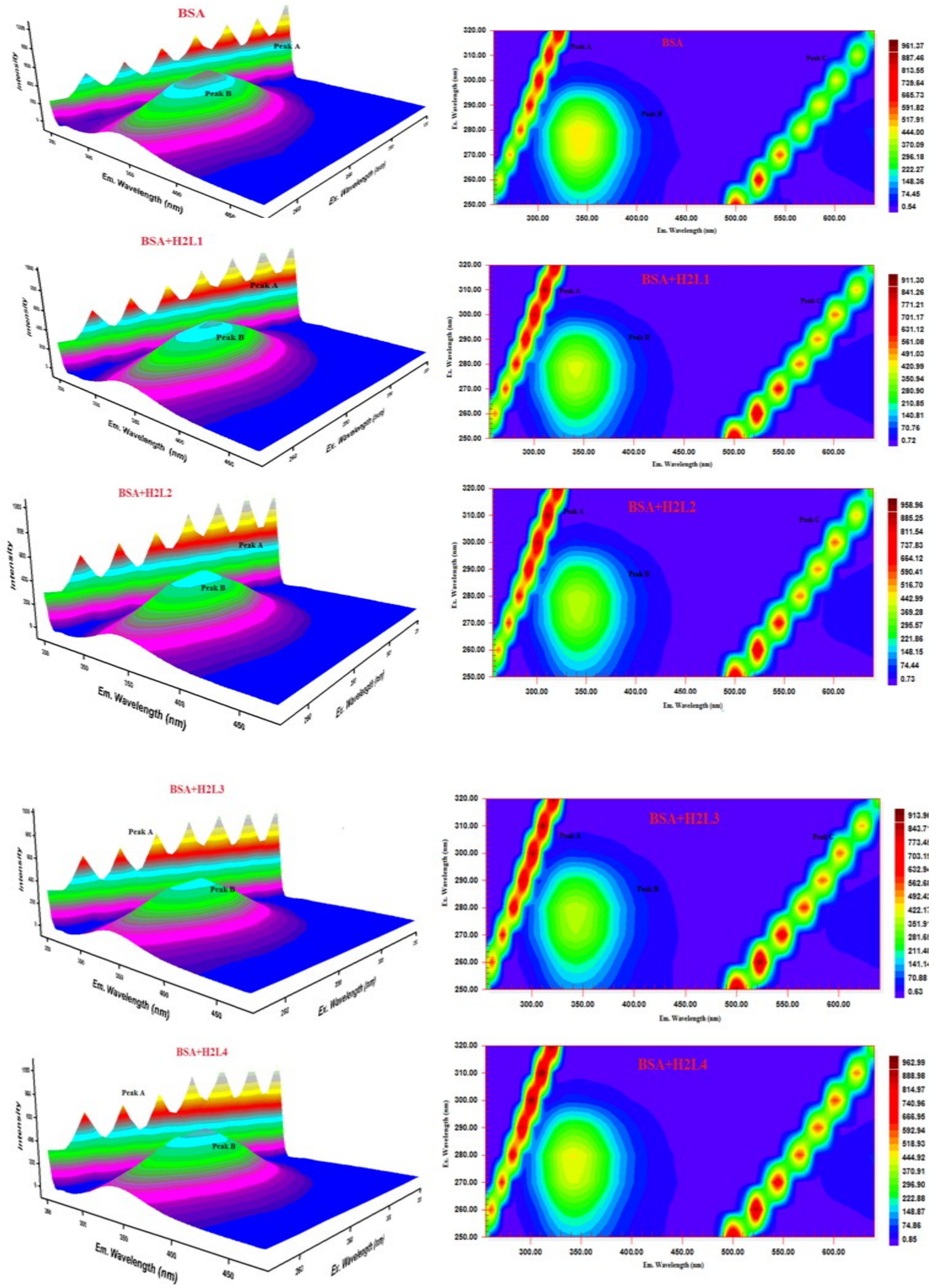

Fig. S34. Three-dimensional fluorescence spectra of BSA in the absence and presence of ligands $\mathbf{H_2L^{1\text{-}4}}$ (pH 7.4, 298 K, [HSA] =10 μM, [Ligand] =10 μM)

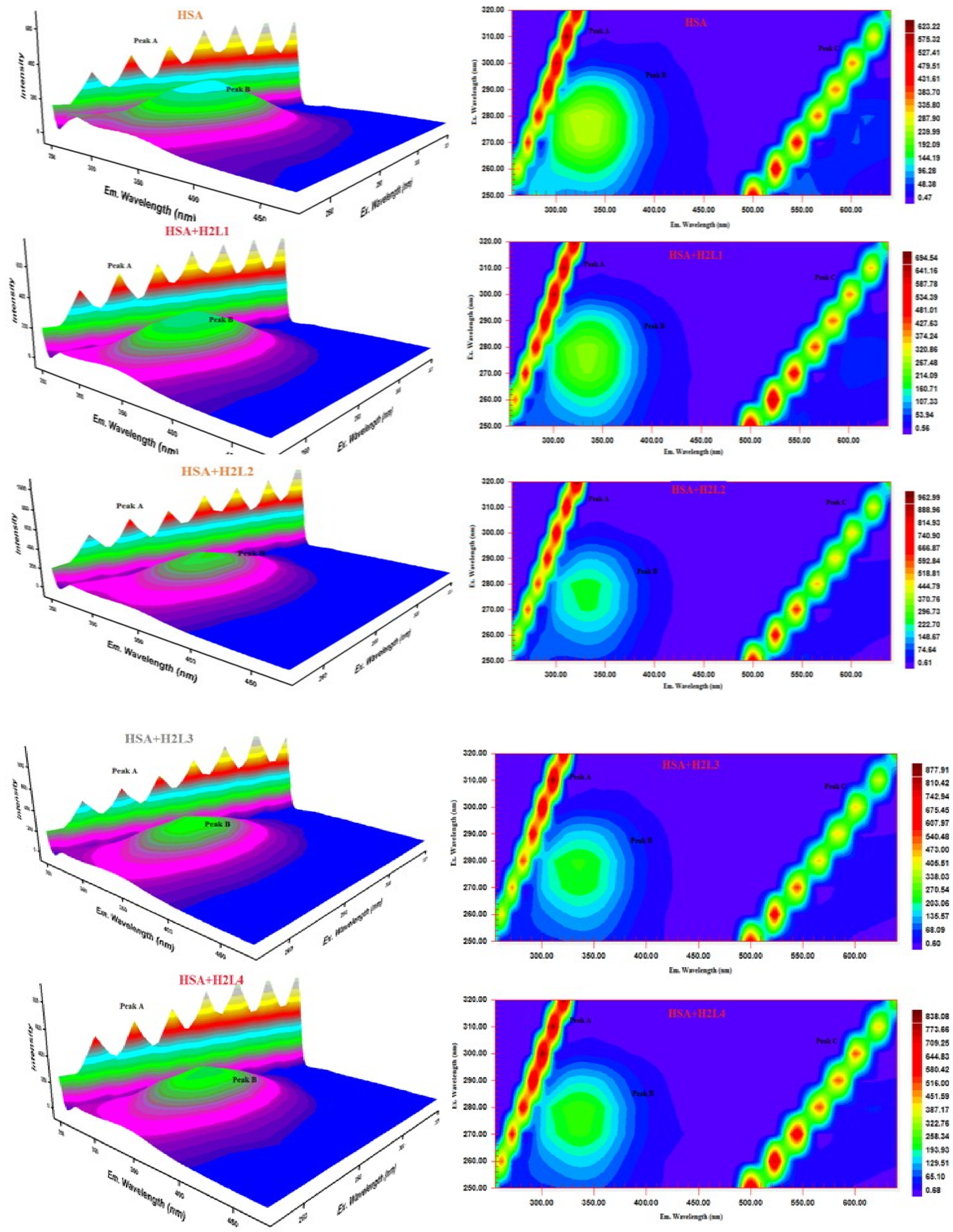

Fig. S35. Three-dimensional fluorescence spectra of HSA in the absence and presence of ligands $\mathbf{H_2L^{1\text{-}4}}$ (pH 7.4, 298 K, [HSA] =10 μM, [Ligand] =10 μM)

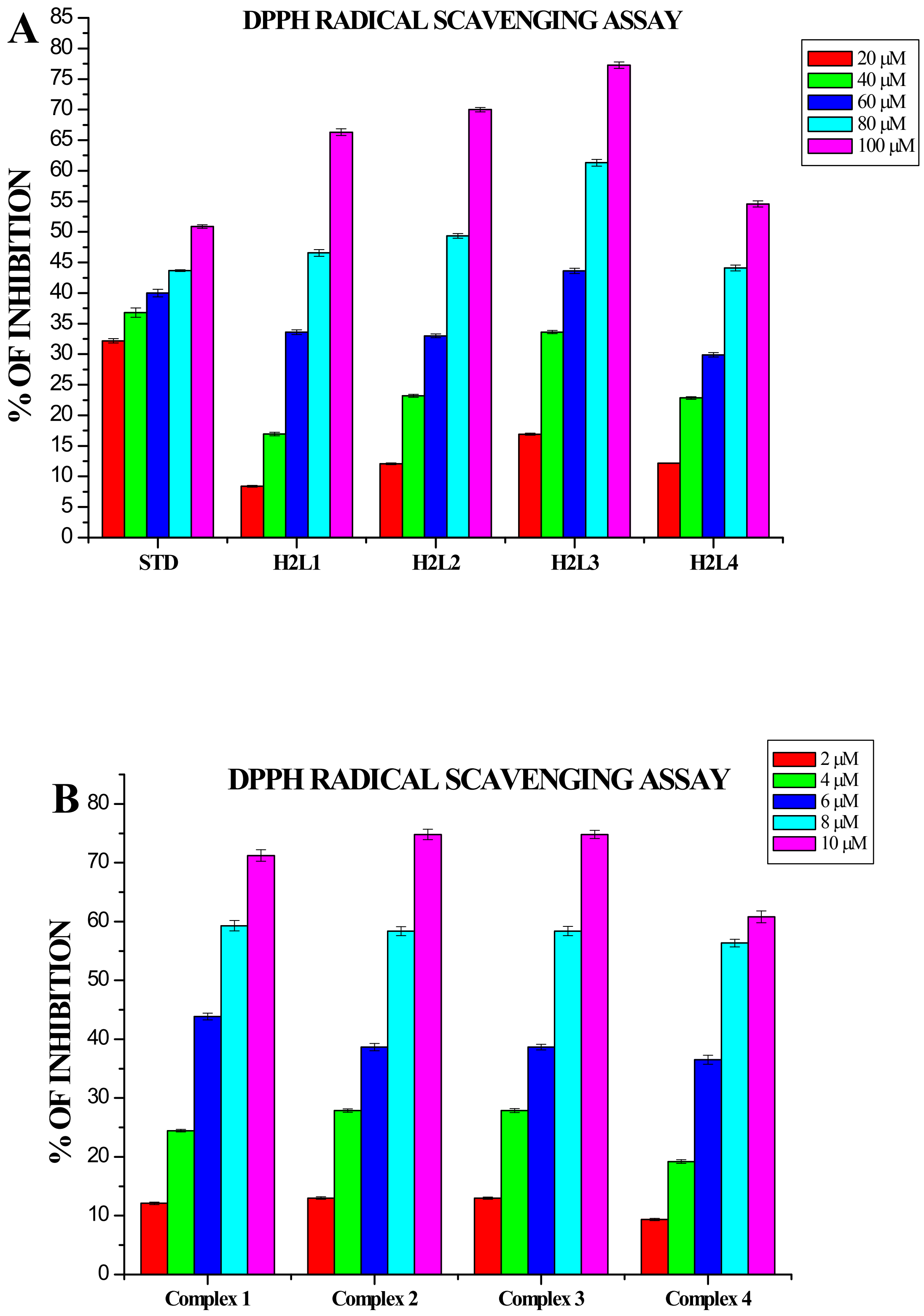

Fig. S36. DPPH scavenging activity of ligands $\mathbf{H_2L^{1\text{-}4}}$, [RuHClCO($PPh_3$)$_3$] and new Ru(II) complexes (**1-4**). Error bars represent the standard deviation of the mean (n=3)
